# Fe-catalyzed efficient synthesis of 2,4- and 4-substituted quinolines via C(sp^2^)–C(sp^2^) bond scission of styrenes

**DOI:** 10.3762/bjoc.21.142

**Published:** 2025-09-05

**Authors:** Prafull A Jagtap, Manish M Petkar, Vaishnavi R Sawant, Bhalchandra M Bhanage

**Affiliations:** 1 Department of Chemistry, Institute of Chemical Technology, Mumbai-400019, India

**Keywords:** C–C activation, C–H annulation, iron metal catalysis, quinolines, styrene

## Abstract

Herein, we report a highly efficient, environmentally benign protocol for the domino synthesis of 2,4-disubstituted and 4-substituted quinoline molecules. The developed strategy involves an earth-abundant Fe-catalyzed C(sp^2^)–C(sp^2^) bond cleavage of styrene, followed by the hydroamination of the cleaved synthons with arylamines and subsequent C–H annulation to yield two valuable quinoline derivatives. Key features of this protocol include the use of O_2_ as an ideal, green oxidant, operational simplicity and scalability, high atom- and step-economy, and cost-effectiveness, collectively enabling the single-step synthesis of two medicinally relevant N-heterocycles in excellent combined yields.

## Introduction

**Figure 1:**
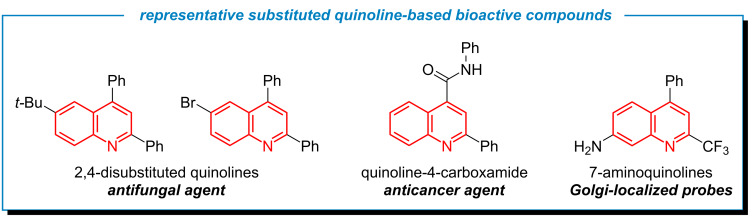
Representative examples of bioactive quinolines.

Quinolines are one of the essential heteroaromatic motifs that play a crucial role across diverse scientific fields due to their wide range of applications. In contemporary medicine, quinoline derivatives frequently appear in active pharmaceutical ingredients, therapeutic agents, and agrochemical formulations [1–9]. Around 60% of recently FDA-approved drugs contain heterocyclic compounds, with quinoline recognized as a key structural motif due to its significant anticancer, antifungal, antibacterial, and anti-inflammatory activities [10–13]. In the field of optoelectronics, especially with 2,4-diarylquinoline derivatives, extensive studies have highlighted their applicability in organic light-emitting diode (OLED) systems as functional materials [14–15] and cutting-edge fluorescent probes for sensing and bioimaging applications (Figure 1) [16–17]. Quinoline-derived metal complexes have also demonstrated broad utility, functioning as effective catalysts in organic synthesis and finding applications across medicinal chemistry, materials science, photovoltaics, and chemical sensing [18].

Due to its wide range of applications, several methods for synthesizing substituted quinoline derivatives have been developed in recent decades, based on mechanisms such as Conrad–Limpach–Knorr [19], Friedländer [20], Doebner−Miller [21], Pfitzinger [22], Skraup [23], Povarov [24], and Combes [25]. However, these methods often require multiple synthetic steps and demanding conditions such as elevated temperatures, strong acidic or basic environments, and the use of expensive metal catalysts, which limit their broader applicability. To overcome these limitations, numerous catalytic strategies have been explored in recent decades for the synthesis of structurally diverse quinolines. Among them, transition-metal-catalyzed multicomponent reactions (MCRs) have emerged as particularly effective for constructing complex quinoline-based heterocycles [26–28]. Catalytic pathways such as cycloaddition, tandem annulation, intramolecular cyclization, and cross-coupling reactions are commonly employed under thermal conditions, utilizing metal catalysts based on Pd, Ru, Au, Cu, and Fe to access a wide array of substituted quinoline frameworks [29–38].

Conversely, in light of climate change and the ongoing energy crisis, there is an urgent need to reform energy and chemical production by prioritizing environmentally sustainable methods that are both practical and broadly implementable. Styrenes are industrially important bulk chemicals [39], with an estimated global production of approximately 30 million tons annually [40]. Their low cost and widespread availability make them highly valuable as fundamental building blocks in organic synthesis. Over the past few decades, the direct functionalization of styrenes has emerged as a prominent research area due to its promising industrial relevance. Oxidative cleavage of alkenes to yield carbonyl compounds is one of the key transformations in synthetic organic chemistry [41–42]. Over the past two decades, this field has witnessed significant advancements, primarily through the use of organic oxidants and transition-metal catalysts. One of the key transformations in organic synthesis is the selective oxidative cleavage of alkenes to yield ketones or aldehydes [43–47]. Traditionally, such transformations have been achieved using various oxidizing agents, transition-metal-based systems, organo- and biocatalysts, as well as enzymatic processes. Among these, molecular oxygen stands out as a greener and more sustainable oxidant due to its natural abundance, low cost, and environmentally friendly properties, making it an appealing option for both academic research and industrial applications. Recently, some advanced strategies have been developed for cleavage of alkenes [48–51].

**Scheme 1:**
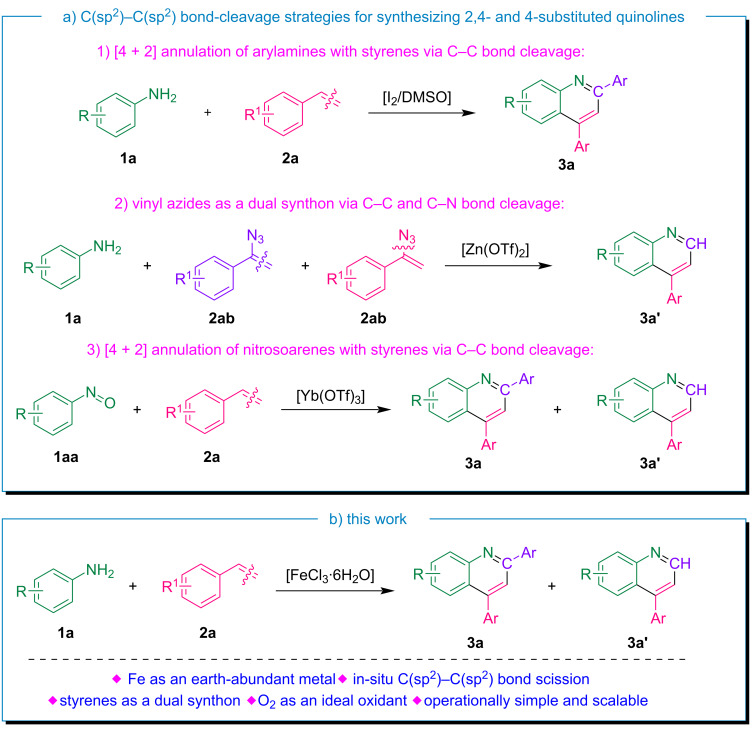
C(sp^2^)–C(sp^2^) bond-cleavage strategies for quinoline synthesis.

Owing to the abundance and versatile applications of styrenes in diverse fields of organic chemistry, some strategies have recently been developed for synthesizing 2,4-disubstituted and 4-substituted quinoline derivatives via the C(sp^2^)–C(sp^2^) bond-scission approach. A summary of known procedures for synthesizing these derivatives via C(sp^2^)–C(sp^2^) bond cleavage is presented in Scheme 1a. In 2015, Shah et al. documented the first two-component, metal-free approach for accessing 2,4-disubstituted quinolines [52]. They employed an I_2_/DMSO-facilitated C–C bond-scission strategy of styrenes, followed by C–N bond formation and subsequent [4 + 2] annulation. Jiang and co-workers developed a method for synthesizing 4-substituted quinolines using vinyl azides as dual synthons, facilitating both the C–C and C–N bond cleavage [53]. In this work, the authors used a stoichiometric amount of Zn(OTf)_3_ as a Lewis acid catalyst and air as the oxidant for the reaction. Jana and colleagues demonstrated an atom-efficient pseudo-three-component C–H annulation reaction catalyzed by Yb and Cu, which involved nitrosoarenes and styrene or epoxystyrene as coupling partners to yield substituted aryl quinolines [54]. However, the methods discussed in the literature often have limitations, such as reliance on stoichiometric amounts of reagents, expensive metal triflates, poor atom economy, and long reaction times. Moreover, many existing methodologies suffer from poor atom utilization, leading to increased waste and reduced overall efficiency, which can be challenging to manage, especially when scaling up.

Building on previous studies, we envisioned a novel reaction system that facilitates the C(sp^2^)–C(sp^2^) bond cleavage of styrene, leading to the in situ generation of two valuable intermediates that act as dual synthons for the synthesis of 2,4- and 4-substituted quinolines. As part of our ongoing efforts to develop innovative C–C and C–H activation strategies for constructing nitrogen-containing heterocycles [55–56], we herein report the first example of the earth-abundant iron-catalyzed oxidative cleavage of C(sp^2^)–C(sp^2^) bonds of styrenes and further utilization of the intermediates as dual synthons for the synthesis of two essential quinoline moieties as shown in Scheme 1b.

## Results and Discussion

To validate our hypothesis, we commenced our investigation by employing arylamine **1a** and styrene (**2a**) as model substrates. The optimized reaction conditions are presented in Table 1. Building upon our previous studies, where we investigated how solvent selection can influence the selective formation of quinoline scaffolds, the present C–H annulation reaction of aniline (**1a**) with styrene (**2a**) was initially carried out in TFE (trifluoroethanol) as solvent in the presence of 25 mol % FeCl_3_·6H_2_O as a catalyst and 1.5 equiv of TFA (trifluoroacetic acid) as an additive (Table 1, entry 1). For this reaction, 12% of 2,4-disubstituted quinolone **3a** along with 36% of 4-substituted quinolone **3a′** were observed during GC and GC–MS analysis, and the structures of both quinoline moieties were identified by ^1^H and ^13^C NMR spectroscopy. Encouraged by this result, we next examined the feasibility of the reaction with methanol as solvent (Table 1, entry 2). We were delighted to observe that, in methanol, the reaction proceeded smoothly, affording 41% and 51% isolated yields of **3a** and **3a′**, respectively. We further explored the effect of other catalysts on the reaction. As listed in Table 1, entries 3–6, among the Lewis acids surveyed, FeCl_3_·6H_2_O provided the best results (Table 1, entry 2). A further reduction in catalyst loading from 25 mol % to 20 mol % and 10 mol % resulted in a noticeable decrease in both selectivity and conversion efficiency (Table 1, entries 7 and 8). This is likely due to insufficient C(sp^2^)–C(sp^2^) bond cleavage as well as inadequate Lewis acid activation of *N*-methylaniline (**1a′**), which hinders its further cyclization into the final product **3a′**.

**Table 1:** Optimization of reaction conditions.^a^

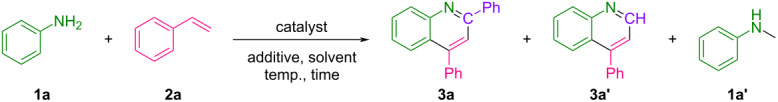								

Entry	Catalyst	Additive	Solvent	Conv. (%)^b^	Selectivity (%)^b^			

					**3a**	**3a′**	**1a′**	**1a′′**

1	FeCl_3_·6H_2_O	TFA	TFE	94	12	36	–	–
2	FeCl_3_·6H_2_O	TFA	MeOH	100	44 (41)^c^	56 (51)^c^	–	–
3	FeCl_3_	TFA	MeOH	96	46	48	–	6
4	FeBr_3_	TFA	MeOH	89	37	42	6	15
5	FeCl_2_·4H_2_O	TFA	MeOH	94	50	47	3	–
6	Fe(OAc)_2_	TFA	MeOH	82	34	45	4	17
7^d^	FeCl_3_·6H_2_O	TFA	MeOH	99	41	55	4	–
8^e^	FeCl_3_·6H_2_O	TFA	MeOH	78	45	50	5	–
9	–	TFA	MeOH	10	–	–	–	100
10	FeCl_3_·6H_2_O	AcOH	MeOH	92	37	58	5	–
11	FeCl_3_·6H_2_O	TsOH	MeOH	91	35	59	1	5
12^f^	FeCl_3_·6H_2_O	TFA	MeOH	96	40	56	4	–
13	FeCl_3_·6H_2_O	–	MeOH	56	40	47	8	5
14^g^	FeCl_3_·6H_2_O	TFA	MeOH	84	40	58	2	–
15^h^	FeCl_3_·6H_2_O	TFA	MeOH	75	32	36	10	22

^a^Reaction conditions: **1a** (0.5 mmol, 1 equiv), **2a** (2.2 equiv), catalyst (25 mol %), TFA (1.5 equiv), in solvent (2 mL), and allowed to stir under an O_2_ (≈1 atm). at 120 °C for 24 h; ^b^conversion and selectivity were determined through GC and GC–MS analysis; ^c^isolated yields after column chromatography; ^d^20 mol % FeCl_3_·6H_2_O; ^e^10 mol % FeCl_3_·6H_2_O; ^f^1 equiv TFA; ^g^100 °C; ^h^1 equiv K_2_S_2_O_8_ as oxidant instead of O_2_, (**1a′′**: other side products of **1a**).

The reaction, when conducted in the absence of a catalyst, failed to proceed, thereby highlighting the crucial role of catalytic activation in facilitating the transformation under the given reaction conditions (Table 1, entry 9). The effect of additives on this transformation was then investigated, and it was found that utilizing AcOH and TsOH additives instead of TFA failed to reach optimal yields (Table 1, entries 10 and 11). A noticeable decrease in yield was observed when the amount of trifluoroacetic acid (TFA) was reduced from 1.5 equiv to 1.0 equiv (Table 1, entry 12). Consequently, in the absence of the additive, the yield was significantly diminished due to the incomplete consumption of **1a** (Table 1, entry 13). Lowering the temperature to 100 °C considerably reduced both the conversion and selectivity (Table 1, entry 14). Furthermore, when K_2_S_2_O_8_ was used as the oxidizing agent instead of O_2_, a considerable drop in both conversion and selectivity was observed.

**Scheme 2:**
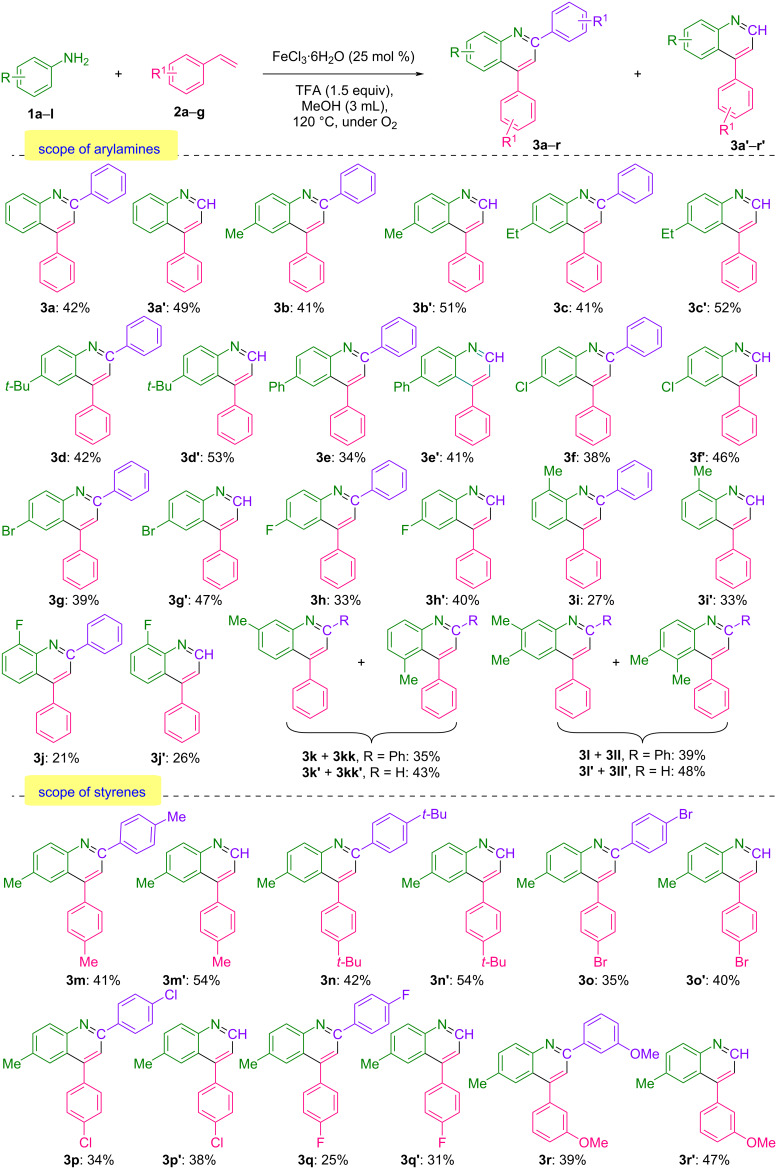
Substrate scope of various arylamines and styrenes.

Having identified satisfactory conditions, we sought to examine the scope and generality of this methodology. As summarized in Scheme 2, we initially investigated the scope and effect of arylamine functionalities on this transformation. Under optimized reaction conditions, arylamines bearing either electron-rich or electron-deficient substituents at the *para*-position demonstrated good tolerance, producing both mono- and disubstituted quinolines with high overall yields. Substrates bearing electron-rich groups at *para*-position such as -Me, -Et, and –*t*-Bu (**1b**–**d**) showed excellent compatibility, producing both 2,4-disubstituted quinolines **3b**–**d** and 4-substituted quinolines **3b′**–**d′** with consistently high combined yields, ranging from 92% to 95%. Probably due to steric hindrance, when the phenyl group was attached to the *para*-position of the aniline (**1e**), the corresponding products **3e** along with **3e′** could be obtained in 75% yields. Electron-deficient groups at the *para*-position, such as -Cl, -Br, and -F (**1f**–**h**), were next evaluated using the optimized conditions, resulting in the isolation of the corresponding quinolines **3f**–**h** and their 4-substituted analogs **3f′**–**h′** with satisfactory yields ranging from 73% to 86%. *ortho*-Substituted arylamines **1i** and **1j** showed sensitivity, likely due to steric hindrance, leading to a moderate combined yield. Moreover, when the *meta*-substituted arylamine **1k** was reacted under standard conditions, regioisomeric forms of quinolines were observed during GC and GC–MS analysis. Nearly identical results were obtained when 3,4-dimethylaniline (**1l**) was used.

An additional effort was made to broaden the scope of this transformation by exploring various functionalities on the styrene substrate as well. Styrene with an electron-rich group at *para*-position such as -Me and –*t*-Bu (**2b** and **2c**) also reacted smoothly to afford the corresponding quinolines **3m** and **3n** along with **3m′** and **3n′** in high yields. Slightly lower yields were observed when styrenes bearing electron-withdrawing substituents (**2d** and **2f**) were examined, giving the final products ranging from 56% to 75% isolated yields. Nevertheless, *meta*-substituted styrene (**2g**) was also evaluated in this reaction, resulting in the formation of both **3r** and **3r′**, which were obtained in 86% combined yield.

Gram-scale studies were conducted to further demonstrate the synthetic potential and practical utility of the developed methodology.

**Scheme 3:**
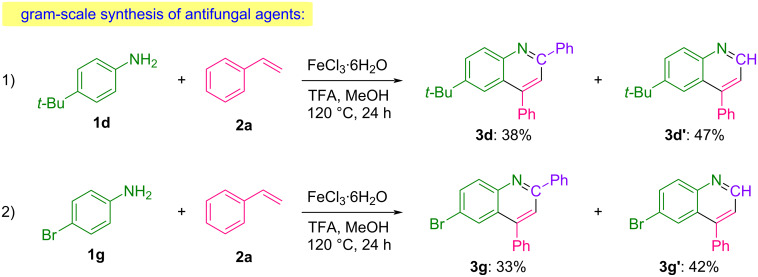
Scale-up studies for the synthesis of antifungal agents.

The biologically significant compounds 6-(*tert*-butyl)-2,4-diphenylquinoline (**3d**) and 6-bromo-2,4-diphenylquinoline (**3g**), both recognized for their antifungal activity [10], were successfully synthesized on a gram scale, showcasing the scalability and efficiency of the process (Scheme 3).

**Scheme 4:**
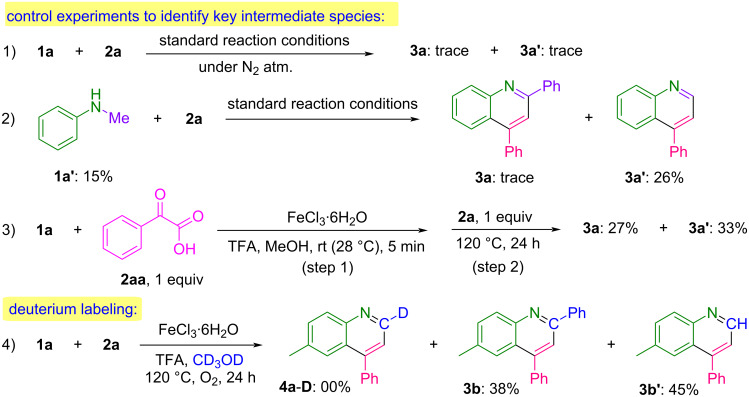
Mechanistic investigations.

After having demonstrated the broad substrate compatibility and the synthetic potential of this protocol, a set of control experiments was conducted to gain insight into the reaction mechanism, as depicted in Scheme 4.

To clarify the significance of oxidative conditions, the standard reaction was initially conducted under an inert atmosphere by replacing oxygen with nitrogen (reaction 1). Under these conditions, the reaction led to a complete suppression in the yield of **3a** and **3a′**. The above results reveal that O_2_ plays a remarkable role in this transformation. Subsequently, the reaction was performed using *N*-methylaniline (**1a′**) under the optimized conditions (reaction 2), which afforded product **3a′** in 26% yield. Then, in reaction 3, a one-pot, two-step reaction involving phenylglyoxalic acid (**2aa**) was carried out. In step 1, compounds **1a** and **2aa** were stirred at room temperature for 5 minutes in the presence of a catalyst and an additive. Subsequently, in step 2, styrene (**2a**) was added, and the reaction mixture was stirred at 120 °C for 24 hours, resulting in the formation of 27% of **3a** along with 33% of **3a′**. To further investigate the role of the solvent in the reaction mechanism, a deuterium-labeling experiment was performed by reacting **1a** with **2a** in deuterated methanol (CD_3_OD) under standard conditions. In this reaction, **4a-D** was not detected, indicating that methanol is not utilized as a C1 source.

**Scheme 5:**
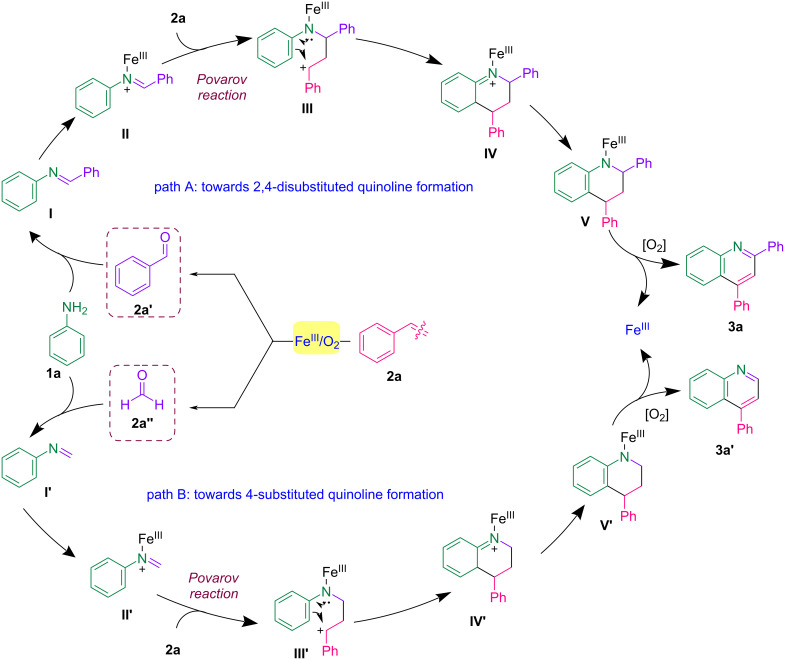
Plausible reaction mechanism.

Considering this experimental evidence and existing literature [57,54], a plausible mechanism is depicted in Scheme 5. The reaction likely proceeds through forming benzaldehyde (**2a′**) (detected during GC–MS analysis) and formaldehyde (**2a′′**) via C–C bond scission of styrene in the presence of Fe^III^/O_2_, possibly through a 1,2-addition of O_2_ to styrene [49,58–60]. This in-situ generated aldehyde species then undergoes condensation with the amine **1a**, leading to the formation of the corresponding imines **I** and **I′**, as supported by the detection of imine **I** during GC–MS analysis. Both imines coordinate with the Lewis acid Fe^III^ forming intermediates **II** and **II′** with enhanced electrophilicity, respectively. Another equivalent of styrene (**2a**) attacks the electrophilic carbon, leading to the formation of intermediates **III** and **III′**. Subsequent electrophilic cyclization/C–H annulation of the aromatic amine, followed by aromatization, afford intermediates **V** and **V′**. The oxidative dehydrogenation of intermediates **V** and **V′** then results in the formation of products **3a** and **3a′** and the regeneration of the Fe^III^ species. An alternative mechanism involving a concerted [4 + 2] cycloaddition between the aza-butadiene moiety in **II** and the alkene, leading to intermediate **IV**, cannot be ruled out.

## Conclusion

In summary, we have successfully developed a highly efficient method for the oxidative C–C bond cleavage of styrenes, catalyzed by earth-abundant iron, followed by the in-situ utilization of the resulting cleaved synthon in a domino process to synthesize highly substituted quinoline derivatives. We have demonstrated that this process can efficiently convert readily available feedstocks, including a broad range of styrenes and arylamines, into valuable quinolines with good to excellent yields under environmentally benign and mild reaction conditions. The successful execution of a scale-up reaction to synthesize single-step antifungal agents emphasizes the significant synthetic potential of this approach in chemical synthesis and drug discovery.

## Supporting Information

File 1 Experimental section, characterization of synthesized compounds, and copies of spectra.

## Data Availability

All data that supports the findings of this study is available in the published article and/or the supporting information of this article.
