# Supplementary material for: Fe-catalyzed efficient synthesis of 2,4- and 4-substituted quinolines via C(sp2)–C(sp2) bond scission of styrenes
Source: Beilstein J Org Chem. 2025 Sep 5;21:1799–807. doi: 10.3762/bjoc.21.142 (PMC12415914; doi:10.3762/bjoc.21.142)
Supplement: File 1 — Experimental section, characterization of synthesized compounds, and copies of spectra. [file Beilstein_J_Org_Chem-21-1799-s001.pdf]

BEILSTEIN JOURNAL OF ORGANIC CHEMISTRY

Supporting Information

for

Fe-catalyzed efficient synthesis of 2,4- and 4-substituted quinolines via C(sp^2)–C(sp^2) bond scission of styrenes

Prafull A. Jagtap, Manish M. Petkar, Vaishnavi R. Sawant and Bhalchandra M. Bhanage

Beilstein J. Org. Chem. **2025,** *21,* 1799–1807. doi:10.3762/bjoc.21.142

Experimental section, characterization of synthesized compounds, and copies of spectra

License and Terms: This is a supporting information file under the terms of the Creative Commons Attribution License (https://creativecommons.org/licenses/by/4.0). Please note that the reuse, redistribution and reproduction in particular requires that the author(s) and source are credited and that individual graphics may be subject to special legal provisions.
The license is subject to the *Beilstein Journal of Organic Chemistry* terms and conditions: (https://www.beilstein-journals.org/bjoc/terms)

Table of contents

Entry	Contents	Page
1	General information	S2
2	General experimental procedures	S2-S3
3	Characterization data of synthesized quinoline derivatives	S4-S18
4	^{1}H and ^{13}C NMR spectra of synthesized quinoline derivatives	S19-S47
5	GC–MS spectra of **3j**, **3j’**, **3k** + **3kk**, **3k’** + **3kk’**, **3l** + **3ll**, **3l’** + **3ll’**, **3q’**	S48-S51
6	References	S51-S52

General information

Unless otherwise noted, all reactions were performed in 25 mL oven-dried reaction tubes. Reagents and solvents were obtained from commercial suppliers and used without further purification. The progress of the reactions was monitored by thin-layer chromatography (TLC), gas chromatography (GC), and gas chromatography–mass spectrometry (GC–MS). TLC was carried out on silica gel 60-F254 plates. GC yields and selectivity were determined using a Thermo Fisher Scientific gas chromatograph. The GC analysis was conducted with a column-flow rate of 2 mL/min, and the oven temperature was programmed from 80 °C to 240 °C at a ramp rate of 10 or 12 °C/min. Mass spectra were obtained using a Shimadzu QP-2010 mass spectrometer. The final compounds were purified through column chromatography using 100–200 mesh silica gel and a mixture of petroleum ether/ethyl acetate as the mobile phase. ^{1}H and ^{13}C NMR spectra were recorded on an Agilent 500 MHz spectrometer using $CDCl_3$ as the solvent. Chemical shifts (δ) are given in parts per million (ppm) relative to tetramethylsilane (TMS), and coupling constants (*J*) are reported in hertz (Hz). The synthesized compounds were characterized by GC–MS, NMR, and high-resolution mass spectrometry (HRMS). HRMS data were acquired using a time-of-flight (TOF) mass spectrometer equipped with an electrospray ionization (ESI) source.

General experimental procedure

An oven-dried 25 mL reaction tube equipped with a Teflon-coated magnetic stirring bar was charged with arylamine **1** (0.5 mmol), alkyne **2** (2.2 equiv), $FeCl_3{\cdot}6H_2O$ (25 mol %). MeOH (2 mL) was added via syringe, followed by the addition of trifluoroacetic acid (TFA, 0.75 mmol). Oxygen gas (O_2) was bubbled through the reaction mixture, after which the tube was evacuated and flushed with O_2. The reaction vessel was then sealed with a Teflon screw cap and the mixture stirred at 120 °C in a preheated oil bath for 24 hours. Upon completion of the reaction, the mixture was allowed to cool and then diluted with 10 mL of ethyl acetate. Saturated aqueous sodium bicarbonate solution (10 mL) was added, and the aqueous layer was extracted with ethyl acetate (3 × 10 mL). The combined organic extracts were washed with brine, dried over anhydrous sodium sulfate (Na_2SO_4), and concentrated under reduced pressure using a rotary evaporator. The crude residue was purified by column chromatography on

silica gel using a petroleum ether/ethyl acetate eluent in a 100:4 ratio to afford the 2,4-disubstituted quinoline derivatives **3a**. In the case of 4-substituted quinoline derivatives **3a′**, purification was accomplished by further elution with a petroleum ether/ethyl acetate mixture in a 100:12 ratio.

General experimental procedure for scale-up studies

An oven-dried 25 mL reaction tube equipped with a Teflon-coated magnetic stirring bar was charged with arylamine **1** (3 mmol), alkyne **2** (2.2 equiv), and $FeCl_3{\cdot}6H_2O$ (25 mol %). MeOH (5 mL) was added via syringe, followed by the addition of trifluoroacetic acid (TFA, 4.5 mmol). Oxygen gas (O_2) was bubbled through the reaction mixture, after which the tube was evacuated and flushed with O_2. The reaction vessel was then sealed with a Teflon screw cap and the mixture stirred at 120 °C in a preheated oil bath for 24 hours. Upon completion of the reaction, the mixture was allowed to cool and then diluted with 20 mL of ethyl acetate. Saturated aqueous sodium bicarbonate solution (20 mL) was added, and the aqueous layer was extracted with ethyl acetate (3 × 10 mL). The combined organic extracts were washed with brine, dried over anhydrous sodium sulfate (Na_2SO_4), and concentrated under reduced pressure using a rotary evaporator. The crude residue was purified by column chromatography on silica gel using a petroleum ether/ethyl acetate eluent in a 100:4 ratio to afford the 2,4-disubstituted quinoline **3a**. In the case of 4-substituted quinoline **3a′**, purification was accomplished by further elution with a petroleum ether/ethyl acetate mixture in a 100:12 ratio.

Characterization data of 2,4- and 4-substituted quinoline derivatives

2,4-Diphenylquinoline (3a) and 4-phenylquinoline (3a'): According to the general procedure, by using petroleum ether/ethyl acetate 100:4 as an eluent, compound **3a** was isolated in 42% yield as a white solid and by using petroleum ether/ethyl acetate 100:12 as an eluent compound **3a'** was isolated in 49% yield as a yellow oil.

2,4-Diphenylquinoline (3a)[1]

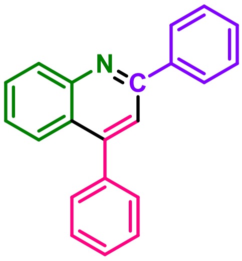

3a

^{1}**H NMR** (500 MHz, $CDCl_3$) δ 8.25 (d, *J* = 8.4 Hz, 1H), 8.19 (d, *J* = 7.4 Hz, 2H), 7.91 (d, *J* = 8.3 Hz, 1H), 7.82 (s, 1H), 7.73 (dd, *J* = 11.3, 4.0 Hz, 1H), 7.59 – 7.44 (m, 9H).

^{13}**C NMR** (126 MHz, $CDCl_3$) δ 156.9, 149.1, 148.8, 139.6, 138.4, 130.1, 129.56, 129.51, 129.3, 128.8, 128.6, 128.4, 127.6, 126.3, 125.7, 125.6, 119.4.

4-Phenylquinoline (3a')[2]

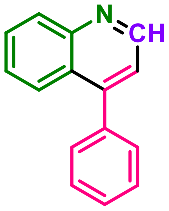

3a'

^{1}**H NMR** (500 MHz, $CDCl_3$) δ 8.95 (d, *J* = 4.2 Hz, 1H), 8.22 (d, *J* = 8.4 Hz, 1H), 7.94 (d, *J* = 8.4 Hz, 1H), 7.74 (t, *J* = 7.5 Hz, 1H), 7.57 – 7.47 (m, 6H), 7.36 (d, *J* = 4.3 Hz, 1H).

^{13}**C NMR** (126 MHz, $CDCl_3$) δ 149.5, 149.0, 148.1, 137.8, 129.6, 129.5, 129.4 128.6, 128.5, 126.79 (d, *J* = 4.1 Hz), 125.9, 121.3.

6-Methyl-2,4-diphenylquinoline (3b) and 6-methyl-4-phenylquinoline (3b'):

According to the general procedure, by using petroleum ether/ethyl acetate 100:4 as an eluent, compound **3b** was isolated in 41% yield as a white solid and by using petroleum ether/ethyl acetate 100:12 as an eluent compound **3b'** was isolated in 51% yield as a yellow oil.

6-Methyl-2,4-diphenylquinoline (3b)[1]

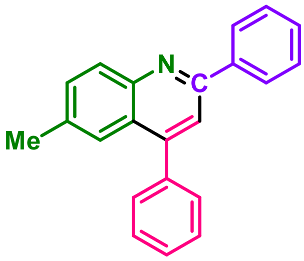

3b

^{1}H NMR (500 MHz, $CDCl_3$) δ 8.15 (dd, *J* = 14.6, 8.0 Hz, 3H), 7.77 (s, 1H), 7.65 (s, 1H), 7.58 – 7.49 (m, 8H), 7.45 (t, *J* = 7.3 Hz, 1H), 2.47 (s, 3H).

^{13}C NMR (126 MHz, $CDCl_3$) δ 156.6, 149.0, 147.9, 140.3, 139.2, 136.8, 132.3, 130.4, 130.1, 129.7, 129.4, 129.1, 128.8, 128.0, 126.2, 124.9, 120.0, 22.4.

6-Methyl-4-phenylquinoline (3b')[3]

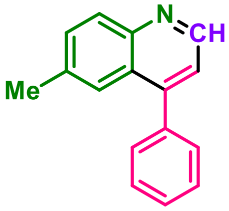

3b'

^{1}H NMR (500 MHz, $CDCl_3$) δ 8.86 (d, *J* = 4.4 Hz, 1H), 8.07 (d, *J* = 8.6 Hz, 1H), 7.66 (s, 1H), 7.58 – 7.48 (m, 6H), 7.29 (d, *J* = 4.4 Hz, 1H), 2.47 (s, 3H).

^{13}C NMR (126 MHz, $CDCl_3$) δ 148.99, 147.8, 147.2, 138.2, 136.5, 131.6, 129.47 (d, *J* = 6.6 Hz), 128.5, 128.3, 126.7, 124.5, 121.4, 21.8.

6-Ethyl-2,4-diphenylquinoline (3c) and 6-ethyl-4-phenylquinoline (3c'):

According to the general procedure, by using petroleum ether/ethyl acetate 100:4 as an eluent, compound **3c** was isolated in 41% yield as a white solid and by using petroleum ether/ethyl acetate 100:12 as an eluent compound **3c'** was isolated in 52% yield as a brown oil.

6-Ethyl-2,4-diphenylquinoline (3c)[4]

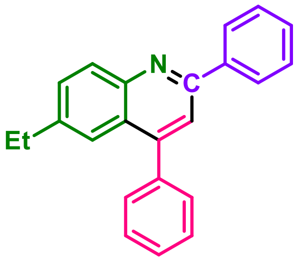

3c

^{1}H NMR (500 MHz, $CDCl_3$) δ 8.18 (d, *J* = 7.3 Hz, 3H), 7.79 (s, 1H), 7.68 (s, 1H), 7.62 (d, *J* = 8.7 Hz, 1H), 7.60 – 7.49 (m, 7H), 7.46 (t, *J* = 7.2 Hz, 1H), 2.78 (q, *J* = 7.5 Hz, 2H), 1.27 (t, *J* = 7.6 Hz, 3H).

^{13}C NMR (126 MHz, $CDCl_3$) δ 156.1, 148.6, 147.5, 142.6, 139.7, 138.6, 130.6, 129.9, 129.5, 129.1, 128.8, 128.6, 128.3, 127.5, 125.7, 123.2, 119.4, 29.1, 15.6.

6-Ethyl-4-phenylquinoline (3c')[5]

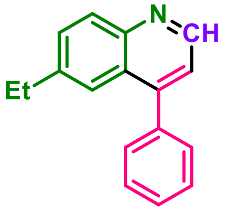

3c'

^{1}H NMR (500 MHz, $CDCl_3$) δ 8.87 (d, *J* = 4.4 Hz, 1H), 8.13 (d, *J* = 8.6 Hz, 1H), 7.69 (s, 1H), 7.61 (dd, *J* = 8.6, 1.6 Hz, 1H), 7.57 – 7.47 (m, 5H), 7.30 (d, *J* = 4.4 Hz, 1H), 2.76 (q, *J* = 7.6 Hz, 2H), 1.25 (t, *J* = 7.6 Hz, 3H).

^{13}C NMR (126 MHz, $CDCl_3$) δ 148.7, 148.3, 147.0, 142.97, 138.1, 130.6, 129.5, 129.3, 128.6, 128.4, 126.7, 123.4, 121.4, 29.1, 15.5.

6-(*tert*-Butyl)-2,4-diphenylquinoline (3d) and 6-(*tert*-butyl)-4-phenylquinoline (3d'):

According to the general procedure, by using petroleum ether/ethyl acetate 100:4 as an eluent, compound **3d** was isolated in 42% yield as a pale yellow semi-solid and by using petroleum ether/ethyl acetate 100:12 as an eluent compound **3d'** was isolated in 53% yield as a brown oil.

6-(*tert*-Butyl)-2,4-diphenylquinoline (3d)[1]

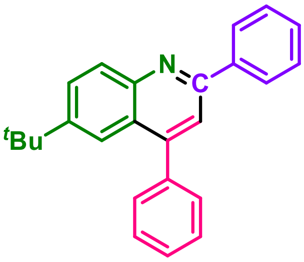

3d

^{1}H NMR (500 MHz, $CDCl_3$) δ 8.23 – 8.16 (m, 3H), 7.89 (d, J = 2.0 Hz, 1H), 7.85 (dd, J = 8.9, 2.1 Hz, 1H), 7.80 (s, 1H), 7.62 – 7.51 (m, 7H), 7.46 (t, J = 7.3 Hz, 1H), 1.37 (s, 9H).

^{13}C NMR (126 MHz, $CDCl_3$) δ 156.3, 149.1, 148.98, 147.3, 139.8, 138.6, 129.6, 129.5, 129.1, 128.8, 128.6, 128.4, 128.3, 127.5, 125.2, 120.5, 119.5, 35.1, 31.2.

6-(*tert*-Butyl)-4-phenylquinoline (3d')[6]

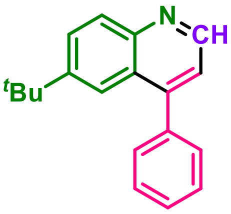

3d'

^{1}H NMR (500 MHz, $CDCl_3$) δ 8.89 (d, *J* = 4.4 Hz, 1H), 8.16 (d, *J* = 8.8 Hz, 1H), 7.89 (d, *J* = 2.0 Hz, 1H), 7.84 (dd, *J* = 8.9, 2.1 Hz, 1H), 7.58 – 7.48 (m, 5H), 7.34 (d, *J* = 4.4 Hz, 1H), 1.34 (s, 9H).

^{13}C NMR (126 MHz, $CDCl_3$) δ 149.7, 149.0, 148.7, 146.5, 138.0, 129.5, 128.7, 128.6, 128.5, 126.3, 121.4, 120.7, 35.1, 31.1.

2,4,6-Triphenylquinoline (3e) and 4,6-diphenylquinoline (3e'):

According to the general procedure, by using petroleum ether/ethyl acetate 100:4 as an eluent, compound **3e** was isolated in 34% yield as a pale yellow solid and by using petroleum ether/ethyl acetate 100:12 as an eluent compound **3e'** was isolated in 41% yield as a white solid.

2,4,6-Triphenylquinoline (3e)[1]

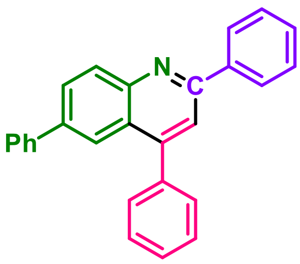

3e

^{1}H NMR (500 MHz, $CDCl_3$) δ 8.39 (d, *J* = 8.6 Hz, 1H), 8.23 (d, *J* = 7.5 Hz, 2H), 8.11 (d, *J* = 1.5 Hz, 1H), 8.03 (dd, *J* = 8.7, 1.7 Hz, 1H), 7.86 (s, 1H), 7.66 – 7.60 (m, 4H), 7.60 – 7.52 (m, 5H), 7.52 – 7.43 (m, 3H), 7.37 (t, *J* = 7.3 Hz, 1H).

^{13}C NMR (126 MHz, $CDCl_3$) δ 156.6, 149.7, 147.8, 140.5, 139.2, 138.2, 130.2, 129.5, 129.4 128.9, 128.7, 128.6, 127.7, 127.6, 127.4, 125.9, 123.4, 119.9.

4,6-Diphenylquinoline (3e')[7]

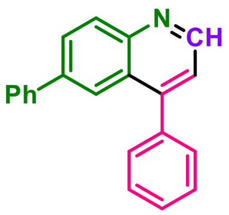

3e'

^{1}H NMR (500 MHz, $CDCl_3$) δ 8.94 (d, *J* = 4.3 Hz, 1H), 8.25 (d, *J* = 8.7 Hz, 1H), 8.11 (d, *J* = 1.1 Hz, 1H), 8.00 (dd, *J* = 8.7, 1.6 Hz, 1H), 7.61 (d, *J* = 7.5 Hz, 2H), 7.58 – 7.48 (m, 5H), 7.45 (t, *J* = 7.6 Hz, 2H), 7.39 – 7.34 (m, 2H).

^{13}C NMR (126 MHz, $CDCl_3$) δ 149.8, 148.7, 147.98, 140.5, 139.4, 137.9, 130.2, 129.5, 129.1, 128.9, 128.7, 128.5, 127.6, 127.5, 126.9 123.6, 121.8.

6-Chloro-2,4-diphenylquinoline (3f) and 6-chloro-4-phenylquinoline (3f'):

According to the general procedure, by using petroleum ether/ethyl acetate 100:5 as an eluent, compound **3f** was isolated in 38% yield as a beige solid and by using petroleum ether/ethyl acetate 100:14 as an eluent compound **3f'** was isolated in 46% yield as a white solid.

6-Chloro-2,4-diphenylquinoline (3f)[4]

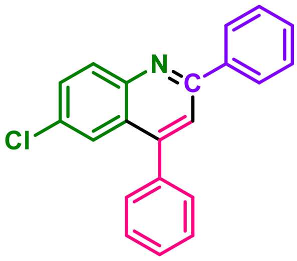

3f

^{1}H NMR (500 MHz, $CDCl_3$) δ 8.18 (dd, *J* = 8.2, 2.2 Hz, 3H), 7.87 (d, *J* = 2.1 Hz, 1H), 7.84 (s, 1H), 7.67 (dd, *J* = 8.9, 2.2 Hz, 1H), 7.60 – 7.46 (m, 8H).

^{13}C NMR (126 MHz, $CDCl_3$) δ 157.1, 148.4, 147.2, 139.2, 137.7, 132.2, 131.7, 130.4, 129.6, 129.4, 128.9, 128.8, 128.7, 127.5, 126.5, 124.5, 120.0.

6-Chloro-4-phenylquinoline (3f')[5]

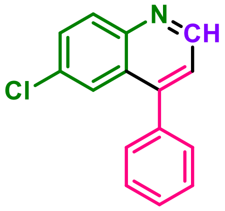

3f'

^{1}H NMR (500 MHz, $CDCl_3$) δ 8.91 (d, *J* = 4.4 Hz, 1H), 8.10 (d, *J* = 9.0 Hz, 1H), 7.88 (d, *J* = 2.0 Hz, 1H), 7.65 (dd, *J* = 9.0, 2.1 Hz, 1H), 7.56 – 7.46 (m, 5H), 7.35 (d, *J* = 4.4 Hz, 1H).

^{13}C NMR (126 MHz, $CDCl_3$) δ 150.0, 147.9, 146.9, 137.2, 132.6, 131.3, 130.3, 129.38 (d, *J* = 1.9 Hz), 128.77 (d, *J* = 5.7 Hz), 127.5, 124.7, 122.1, 121.8.

6-Bromo-2,4-diphenylquinoline (3g) and 6-bromo-4-phenylquinoline (3g'):

According to the general procedure, by using petroleum ether/ethyl acetate 100:5 as an eluent, compound **3g** was isolated in 39% yield as a pale yellow crystals and by using petroleum ether/ethyl acetate 100:14 as an eluent compound **3g'** was isolated in 47% yield as a pale yellow solid.

6-Bromo-2,4-diphenylquinoline (3g)[1]

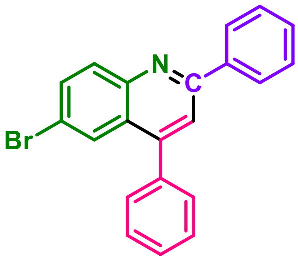

3g

^{1}H NMR (500 MHz, $CDCl_3$) δ 8.17 (d, *J* = 7.6 Hz, 2H), 8.10 (d, *J* = 8.9 Hz, 1H), 8.03 (d, *J* = 1.5 Hz, 1H), 7.82 (s, 1H), 7.79 (dd, *J* = 8.9, 1.5 Hz, 1H), 7.61 – 7.45 (m, 8H).

^{13}C NMR (126 MHz, $CDCl_3$) δ 157.2, 148.4, 147.4, 139.1, 137.7, 133.0, 131.8, 129.6, 129.4, 128.9, 128.8, 128.7, 127.8, 127.5, 126.97, 120.4, 120.0.

6-Bromo-4-phenylquinoline (3g')[7]

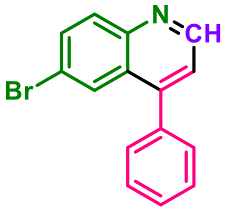

3g'

^{1}H NMR (500 MHz, $CDCl_3$) δ 8.94 (d, *J* = 4.4 Hz, 1H), 8.05 (t, *J* = 5.7 Hz, 2H), 7.79 (dd, *J* = 8.9, 2.1 Hz, 1H), 7.54 (ddd, *J* = 10.4, 7.7, 2.4 Hz, 3H), 7.50 – 7.46 (m, 2H), 7.35 (d, *J* = 4.4 Hz, 1H).

^{13}C NMR (126 MHz, $CDCl_3$) δ 150.2, 147.7, 147.2, 137.2, 132.8, 131.5, 129.4, 128.8, 128.7, 127.97 (d, *J* = 3.9 Hz), 122.0, 120.9.

6-Fluoro-2,4-diphenylquinoline (3h) and 6-fluoro-4-phenylquinoline (3h'):

According to the general procedure, by using petroleum ether/ethyl acetate 100:5 as an eluent, compound **3h** was isolated in 33% yield as a colourless crystals and by using petroleum ether/ethyl acetate 100:14 as an eluent compound **3h'** was isolated in 40% yield as a yellow oil.

6-Fluoro-2,4-diphenylquinoline (3h)[1]

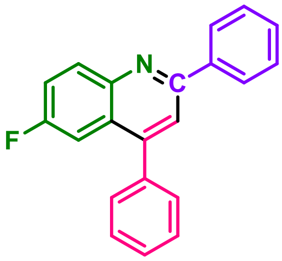

3h

^{1}H NMR (500 MHz, $CDCl_3$) δ 8.24 (dd, *J* = 9.0, 5.5 Hz, 1H), 8.20 – 8.15 (m, 2H), 7.84 (s, 1H), 7.59 – 7.45 (m, 10H).

^{13}C NMR (126 MHz, $CDCl_3$) δ 161.5, 159.6, 156.30 (d, *J* = 2.8 Hz), 148.71 (d, *J* = 5.7 Hz), 145.9, 139.3, 137.9, 132.49 (d, *J* = 9.1 Hz), 129.4, 129.3, 128.9, 128.8, 128.6, 127.4, 126.50 (d, *J* = 9.6 Hz), 119.9, 119.8, 119.6, 109.1, 108.97.

6-Fluoro-4-phenylquinoline (3h')[5]

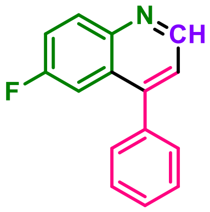

3h'

^{1}H NMR (500 MHz, $CDCl_3$) δ 8.91 (d, *J* = 3.6 Hz, 1H), 8.21 (dd, *J* = 9.1, 5.6 Hz, 1H), 7.56 – 7.47 (m, 6H), 7.37 (d, *J* = 4.1 Hz, 1H).

^{13}C NMR (126 MHz, $CDCl_3$) δ 161.7, 159.7, 148.91 (d, *J* = 2.5 Hz), 148.42 (d, *J* = 5.7 Hz), 145.3, 137.4, 132.01 (d, *J* = 9.2 Hz), 129.3, 128.77 (d, *J* = 4.0 Hz), 127.61 (d, *J* = 9.7 Hz), 121.8, 119.9, 119.7, 109.3, 109.1.

8-Methyl-2,4-diphenylquinoline (3i) and 8-methyl-4-phenylquinoline (3i’):

According to the general procedure, by using petroleum ether/ethyl acetate 100:4 as an eluent, compound **3i** was isolated in 27% yield as a pale yellow oil and by using petroleum ether/ethyl acetate 100:12 as an eluent compound **3i’** was isolated in 33% yield as a pale yellow oil.

8-Methyl-2,4-diphenylquinoline (3i)[4]

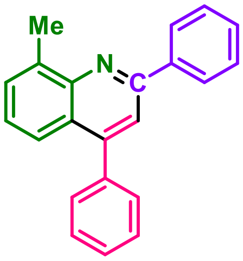

3i

^{1}H NMR (500 MHz, $CDCl_3$) δ 8.31 – 8.27 (m, 2H), 7.84 (s, 1H), 7.73 (d, *J* = 8.3 Hz, 1H), 7.61 – 7.43 (m, 9H), 7.35 (dd, *J* = 8.2, 7.1 Hz, 1H), 2.95 (s, 3H).

^{13}C NMR (126 MHz, $CDCl_3$) δ 154.9, 149.3, 147.6, 139.8, 138.9, 137.9, 129.59 (d, *J* = 3.6 Hz), 129.2, 128.7, 128.5, 128.2, 127.5, 125.9, 125.6, 123.6, 118.6, 18.4.

8-Methyl-4-phenylquinoline (3i’)[2]

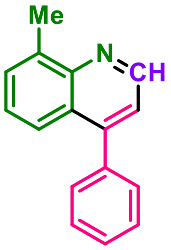

3i'

^{1}H NMR (500 MHz, $CDCl_3$) δ 9.02 (d, *J* = 3.8 Hz, 1H), 7.79 (d, *J* = 8.4 Hz, 1H), 7.61 (d, *J* = 6.8 Hz, 1H), 7.52 (dt, *J* = 13.3, 6.8 Hz, 5H), 7.42 (t, *J* = 7.7 Hz, 1H), 7.38 (d, *J* = 3.6 Hz, 1H), 2.90 (s, 3H).

^{13}C NMR (126 MHz, $CDCl_3$) δ 149.7, 148.2, 138.1, 136.8, 130.1, 129.5, 128.51 (d, *J* = 6.1 Hz), 126.8, 126.6, 124.1, 121.2, 18.7.

8-Fluoro-2,4-diphenylquinoline (3j) and 8-fluoro-4-phenylquinoline (3j’):

According to the general procedure, by using petroleum ether/ethyl acetate 100:5 as an eluent, compound **3j** was isolated in 21% yield as a pale yellow oil and by using petroleum ether/ethyl acetate 100:14 as an eluent compound **3j’** was isolated in 26% yield as a pale yellow oil.

8-Fluoro-2,4-diphenylquinoline (3j)[4]

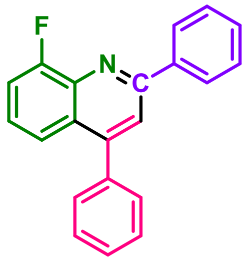

3j

GC-MS (EI, 70 eV) *m/z* (% relative intensity) **298** (100, M^+), 277 (10), 220 (15), 150 (14), 139 (7).

8-Fluoro-4-phenylquinoline (3j')[2]

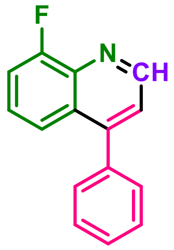

3j'

GC-MS (EI, 70 eV) *m/z* (% relative intensity) **223** (100, M^+), 175 (10), 111 (16), 97 (9).

7-Methyl-2,4-diphenylquinoline (3k) + 5-methyl-2,4-diphenylquinoline (3kk) and 7-methyl-4-phenylquinoline (3k') + 5-methyl-4-phenylquinoline (3kk'):

According to the general procedure, the mixture of regioisomers **3k** + **3kk** was formed in 35% yield and the mixture of regioisomers **3k'** + **3kk'** was formed in 43% yield.

7-Methyl-2,4-diphenylquinoline (3k) + 5-methyl-2,4-diphenylquinoline (3kk)[8]

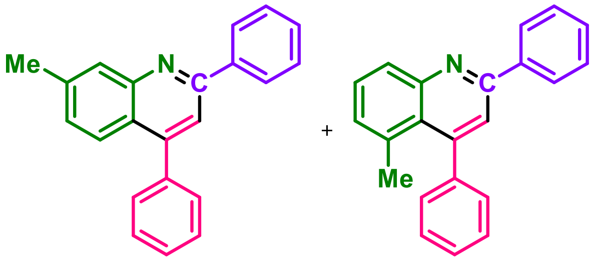

3k 3kk

GC-MS (EI, 70 eV) *m/z* (% relative intensity) **295** (100, M^+), 280 (50), 216 (14), 189 (13), 139 (23).

7-Methyl-4-phenylquinoline (3k') + 5-methyl-4-phenylquinoline (3kk')[2]

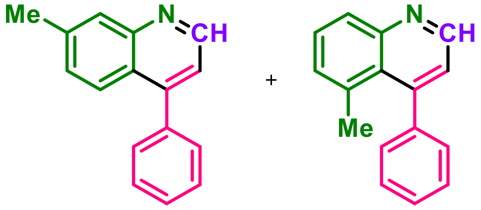

3k' **3kk'**

GC-MS (EI, 70 eV) *m/z* (% relative intensity) **219** (100, M$^+$), 204 (55), 189 (10), 151 (5),

108 (33), 95 (23).

6,7-Dimethyl-2,4-diphenylquinoline (3l) and 5,6-dimethyl-2,4-diphenylquinoline (3ll):

According to the general procedure, the mixture of regioisomers **3l** + **3ll** was formed in 39% yield and the mixture of regioisomers **3l'** + **3ll'** was formed in 48% yield.

6,7-Dimethyl-2,4-diphenylquinoline (3l) + 5,6-dimethyl-2,4-diphenylquinoline (3ll)[8]

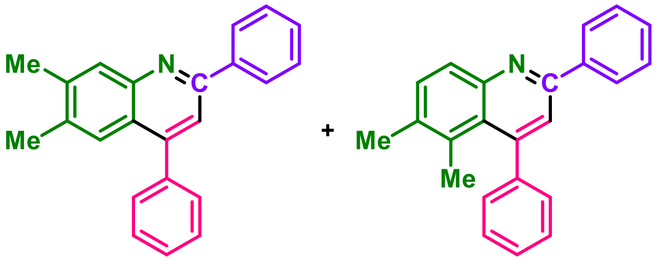

3l **3ll**

GC-MS (EI, 70 eV) *m/z* (% relative intensity) **309** (100, M$^+$), 294 (85), 230 (7), 216 (9),

189 (7), 155 (10), 147 (28).

6,7-Dimethyl-4-phenylquinoline (3l’) + 5,6-dimethyl-4-phenylquinoline (3ll’)[9]

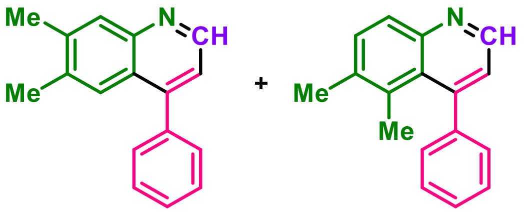

3l'

3ll'

GC-MS (EI, 70 eV) *m/z* (% relative intensity) **233** (100, M^+), 218 (90), 189 (14), 165 (5),

108 (51), 95 (9).

6-Methyl-2,4-di-*p*-tolylquinoline (3m) and 6-methyl-4-(*p*-tolyl)quinoline (3m’):

According to the general procedure, by using petroleum ether/ethyl acetate 100:5 as an eluent, compound **3m** was isolated in 41% yield as a pale yellow solid and by using petroleum ether/ethyl acetate 100:14 as an eluent compound **3m’** was isolated in 54% yield as a pale yellow solid.

6-Methyl-2,4-di-*p*-tolylquinoline (3m)[6]

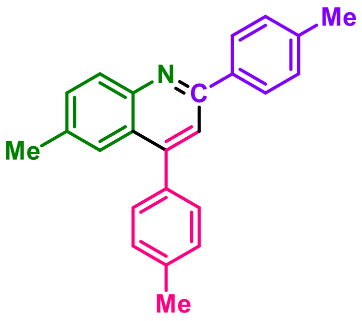

3m

^{1}H NMR (500 MHz, $CDCl_3$) δ 8.11 (d, *J* = 8.6 Hz, 1H), 8.07 (d, *J* = 8.1 Hz, 2H), 7.74 (s, 1H), 7.66 (s, 1H), 7.54 (dd, *J* = 8.6, 1.6 Hz, 1H), 7.45 (d, *J* = 7.9 Hz, 2H), 7.35 (d, *J* = 7.8 Hz, 2H), 7.31 (d, *J* = 8.0 Hz, 2H), 2.48 (s, 3H), 2.46 (s, 3H), 2.42 (s, 3H).

^{13}C NMR (126 MHz, $CDCl_3$) δ 155.98, 148.4, 147.3, 139.1, 138.1, 136.9, 135.9, 135.7, 131.6, 129.7, 129.5, 129.4, 129.3, 127.3, 125.7, 124.4, 119.2, 21.8, 21.3.

6-Methyl-4-(*p*-tolyl)quinoline (3m')[3]

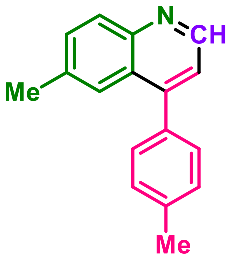

3m'

^{1}H NMR (500 MHz, $CDCl_3$) δ 8.85 (d, *J* = 4.3 Hz, 1H), 8.06 (d, *J* = 8.6 Hz, 1H), 7.69 (s, 1H), 7.55 (d, *J* = 8.1 Hz, 1H), 7.40 (d, *J* = 7.9 Hz, 2H), 7.35 (d, *J* = 7.8 Hz, 2H), 7.28 (d, *J* = 4.4 Hz, 1H), 2.47 (s, 3H), 2.47 (s, 3H).

^{13}C NMR (126 MHz, $CDCl_3$) δ 148.9, 147.9, 147.1, 138.2, 136.5, 135.2, 131.5, 129.4, 129.3, 129.2, 126.8, 124.6, 121.4, 21.8, 21.3.

2,4-Bis(4-(*tert*-butyl)phenyl)-6-methylquinoline (3n) and 4-(4-(*tert*-butyl)phenyl)-6-methylquinoline (3n'):

According to the general procedure, by using petroleum ether/ethyl acetate 100:5 as an eluent, compound **3n** was isolated in 42% yield as a pale yellow oil and by using petroleum ether/ethyl acetate 100:14 as an eluent compound **3n'** was isolated in 54% yield as a white solid.

2,4-Bis(4-(*tert*-butyl)phenyl)-6-methylquinoline (3n)

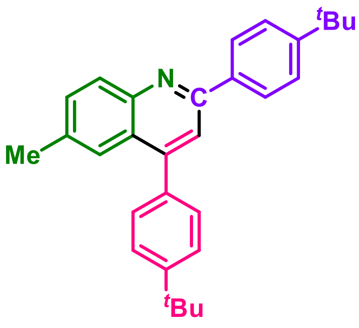

3n

^{1}H NMR (500 MHz, $CDCl_3$) δ 8.10 (dd, *J* = 16.3, 8.5 Hz, 3H), 7.76 (s, 1H), 7.71 (s, 1H), 7.54 (ddd, *J* = 23.1, 13.6, 8.2 Hz, 7H), 2.48 (s, 3H), 1.43 (s, 9H), 1.37 (s, 9H).

^{13}C NMR (126 MHz, $CDCl_3$) δ 156.0, 152.2, 151.3, 148.2, 147.4, 137.1, 135.9, 135.7, 131.6, 129.8, 129.3, 127.2, 125.71 (d, *J* = 7.9 Hz), 125.5, 124.5, 119.4, 34.74 (d, *J* = 5.2 Hz), 31.36 (d, *J* = 13.2 Hz), 21.8.

4-(4-(*tert*-Butyl)phenyl)-6-methylquinoline (3n')[3]

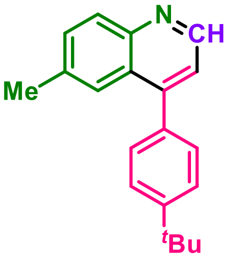

3n'

^{1}H NMR (500 MHz, $CDCl_3$) δ 8.86 (d, *J* = 4.4 Hz, 1H), 8.07 (d, *J* = 8.6 Hz, 1H), 7.74 (s, 1H), 7.55 (d, *J* = 8.2 Hz, 3H), 7.45 (d, *J* = 8.2 Hz, 2H), 7.29 (d, *J* = 4.4 Hz, 1H), 2.48 (s, 3H), 1.42 (s, 9H).

^{13}C NMR (126 MHz, $CDCl_3$) δ 151.4, 149.0, 147.8, 147.2, 136.4, 135.2, 131.5, 129.4, 129.2, 126.8, 125.5, 124.7, 121.4, 34.7, 31.4, 21.8.

2,4-Bis(4-bromophenyl)-6-methylquinoline (3o) and 4-(4-bromophenyl)-6-methylquinoline (3o'):

According to the general procedure, by using petroleum ether/ethyl acetate 100:5 as an eluent, compound **3o** was isolated in 35% yield as a white solid and by using petroleum ether/ethyl acetate 100:14 as an eluent compound **3o'** was isolated in 40% yield as a beige solid.

2,4-Bis(4-bromophenyl)-6-methylquinoline (3o)[10]

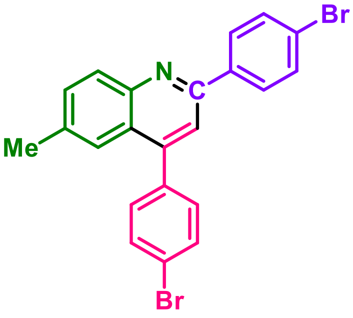

3o

^{1}H NMR (500 MHz, $CDCl_3$) δ 8.14 – 8.04 (m, 3H), 7.72 – 7.68 (m, 3H), 7.67 – 7.63 (m, 2H), 7.60 – 7.56 (m, 2H), 7.44 – 7.40 (m, 2H), 2.48 (s, 3H).

^{13}C NMR (126 MHz, $CDCl_3$) δ 154.7), 147.4, 147.3, 138.3, 137.3, 136.8, 132.1, 131.9, 131.8, 131.1, 129.8, 128.9, 125.4, 124.0, 123.8, 122.7, 118.7, 21.9.

4-(4-Bromophenyl)-6-methylquinoline (3o')[3]

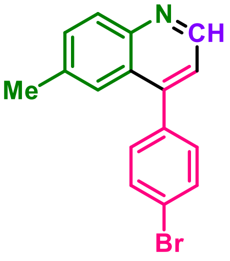

3o'

^{1}H NMR (500 MHz, $CDCl_3$) δ 8.87 (d, *J* = 4.4 Hz, 1H), 8.11 (d, *J* = 8.5 Hz, 1H), 7.68 (d, *J* = 8.3 Hz, 2H), 7.62 – 7.56 (m, 2H), 7.38 (d, *J* = 8.3 Hz, 2H), 7.28 (d, *J* = 4.4 Hz, 1H), 2.48 (s, 3H).

^{13}C NMR (126 MHz, $CDCl_3$) δ 148.5, 146.97, 146.7, 137.1, 136.9, 131.99, 131.8, 131.1, 129.2, 126.4, 124.2, 122.8, 121.2, 21.8.

2,4-Bis(4-chlorophenyl)-6-methylquinoline (3p) and 4-(4-chlorophenyl)-6-methylquinoline (3p'):

According to the general procedure, by using petroleum ether/ethyl acetate 100:5 as an eluent, compound **3p** was isolated in 34% yield as a white solid and by using petroleum ether/ethyl acetate 100:14 as an eluent compound **3p'** was isolated in 38% yield as a pale yellow solid.

2,4-Bis(4-chlorophenyl)-6-methylquinoline (3p)[10]

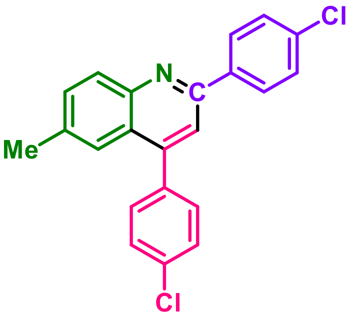

3p

^{1}H NMR (500 MHz, $CDCl_3$) δ 8.14 – 8.10 (m, 3H), 7.70 (s, 1H), 7.58 (d, *J* = 8.2 Hz, 2H), 7.54 (d, *J* = 8.4 Hz, 2H), 7.49 (dd, *J* = 8.4, 1.7 Hz, 4H), 2.48 (s, 3H).

^{13}C NMR (126 MHz, $CDCl_3$) δ 154.6, 147.34 (d, *J* = 12.7 Hz), 137.9, 136.81 (d, *J* = 1.8 Hz), 135.4, 132.1, 130.8, 129.8, 129.0, 128.9, 128.7, 125.5, 124.0, 118.8, 21.8.

4-(4-Chlorophenyl)-6-methylquinoline (3p')[3]

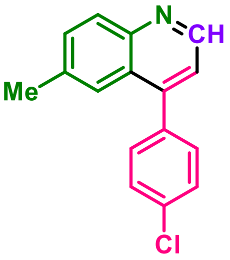

3p'

^{1}H NMR (500 MHz, $CDCl_3$) δ 8.87 (d, *J* = 4.4 Hz, 1H), 8.08 (d, *J* = 8.5 Hz, 1H), 7.58 (dd, *J* = 11.8, 3.3 Hz, 2H), 7.52 (d, *J* = 8.3 Hz, 2H), 7.44 (d, *J* = 8.3 Hz, 2H), 7.27 (s, 1H), 2.48 (s, 3H).

^{13}C NMR (126 MHz, $CDCl_3$) δ 148.9, 147.1, 146.5, 136.8, 136.6, 134.5, 131.7, 130.8, 129.5, 128.8, 126.5, 124.2, 121.3, 21.8.

2,4-Bis(4-fluorophenyl)-6-methylquinoline (3q) and 4-(4-fluorophenyl)-6-methylquinoline (3q'):

According to the general procedure, by using petroleum ether/ethyl acetate 100:5 as an eluent, compound **3q** was isolated in 25% yield as a white solid and by using petroleum ether/ethyl acetate 100:14 as an eluent compound **3q'** was isolated in 31% yield as a yellow oil.

2,4-Bis(4-fluorophenyl)-6-methylquinoline (3q)[10]

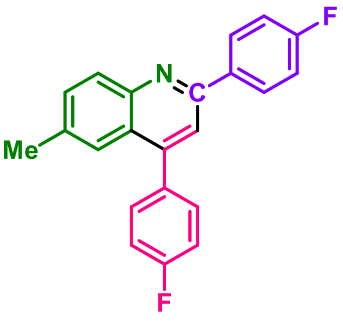

3q

^{1}H NMR (500 MHz, $CDCl_3$) δ 8.18 – 8.14 (m, 2H), 8.11 (d, *J* = 8.7 Hz, 1H), 7.69 (s, 1H), 7.57 (d, *J* = 8.1 Hz, 2H), 7.54 – 7.49 (m, 2H), 7.23 (ddd, *J* = 28.4, 12.9, 5.4 Hz, 5H), 2.48 (s, 3H).

^{13}C NMR (126 MHz, $CDCl_3$) δ 164.7, 163.8, 162.7, 161.9, 154.8, 147.6, 147.2, 136.5, 135.66 (d, *J* = 3.0 Hz), 134.39 (d, *J* = 3.4 Hz), 132.0, 131.18 (d, *J* = 8.1 Hz), 129.7, 129.29 (d, *J* = 8.4 Hz), 125.6, 124.1, 119.1, 115.71 (dd, *J* = 21.6, 9.4 Hz), 21.8.

4-(4-Fluorophenyl)-6-methylquinoline (3q')[3]

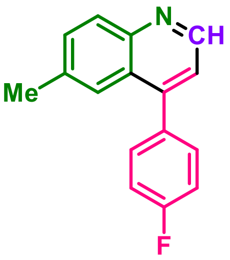

3q'

GC-MS (EI, 70 eV) *m/z* (% relative intensity) **237** (100, M^+), 222 (67), 194 (6), 117 (24) 111 (17).

2,4-Bis(3-methoxyphenyl)-6-methylquinoline (3r) and 4-(3-methoxyphenyl)-6-methylquinoline (3r'):

According to the general procedure, by using petroleum ether/ethyl acetate 100:8 as an eluent, compound **3r** was isolated in 39% yield as a pale yellow solid and by using petroleum ether/ethyl acetate 100:20 as an eluent compound **3r'** was isolated in 47% yield as a pale yellow solid.

2,4-Bis(3-methoxyphenyl)-6-methylquinoline (3r)

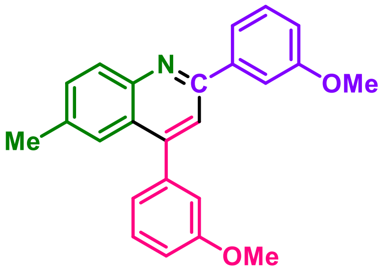

3r

^{1}H NMR (500 MHz, $CDCl_3$) δ 8.13 (d, *J* = 8.6 Hz, 1H), 7.77 (d, *J* = 4.2 Hz, 2H), 7.71 (d, *J* = 7.8 Hz, 1H), 7.67 (s, 1H), 7.56 (dd, *J* = 8.6, 1.7 Hz, 1H), 7.44 (dt, *J* = 23.9, 7.9 Hz, 2H), 7.13 (d, *J* = 7.5 Hz, 1H), 7.10 – 7.03 (m, 2H), 7.00 (dd, *J* = 8.1, 2.3 Hz, 1H), 3.93 (s, 3H), 3.88 (s, 3H), 2.48 (s, 3H).

^{13}C NMR (126 MHz, $CDCl_3$) δ 160.1, 159.6, 155.8, 148.3, 147.2, 141.2, 139.9, 136.3, 131.8, 129.78 (d, *J* = 2.7 Hz), 129.6, 125.7, 124.4, 121.97, 119.9, 119.4, 115.3, 115.1, 113.8, 112.5, 55.42 (d, *J* = 2.9 Hz), 21.8.

4-(3-Methoxyphenyl)-6-methylquinoline (3r')

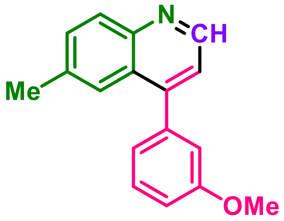

3r'

^{1}H NMR (500 MHz, $CDCl_3$) δ 8.87 (d, *J* = 4.4 Hz, 1H), 8.07 (d, *J* = 8.6 Hz, 1H), 7.68 (s, 1H), 7.56 (dd, *J* = 8.6, 1.6 Hz, 1H), 7.45 (t, *J* = 7.8 Hz, 1H), 7.30 (d, *J* = 4.4 Hz, 1H), 7.10 – 7.01 (m, 3H), 3.87 (s, 3H), 2.47 (s, 3H).

^{13}C NMR (126 MHz, $CDCl_3$) δ 159.6, 148.9, 147.8, 147.0, 139.5, 136.6, 131.7, 129.6, 129.3, 126.7, 124.6, 121.9, 121.3, 115.1, 113.7, 55.4, 21.8.

^{1}H and ^{13}C NMR spectra of synthesized quinoline derivatives

^{1}H NMR (500 MHz, $CDCl_3$) of 2,4-diphenylquinoline (3a)

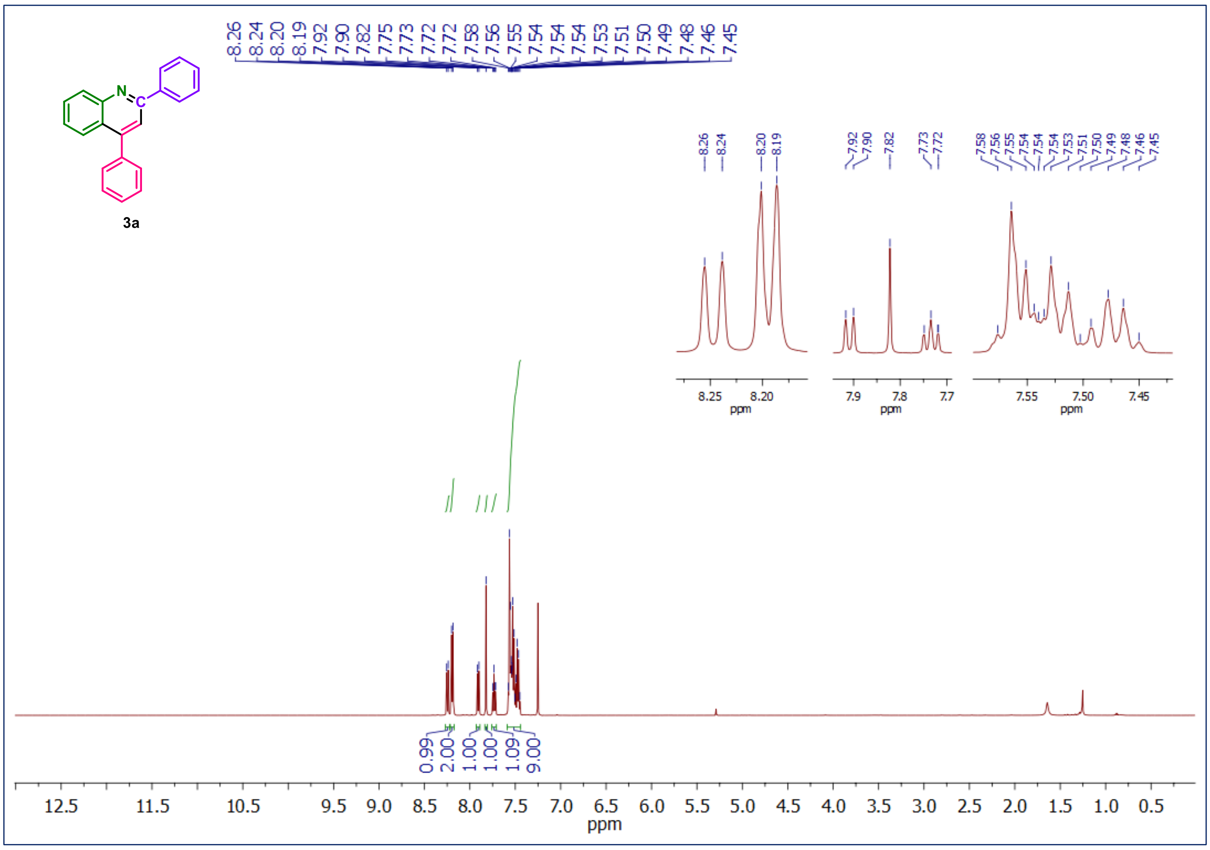

^{13}C{^{1}H} NMR (126 MHz, $CDCl_3$) of 2,4-diphenylquinoline (3a)

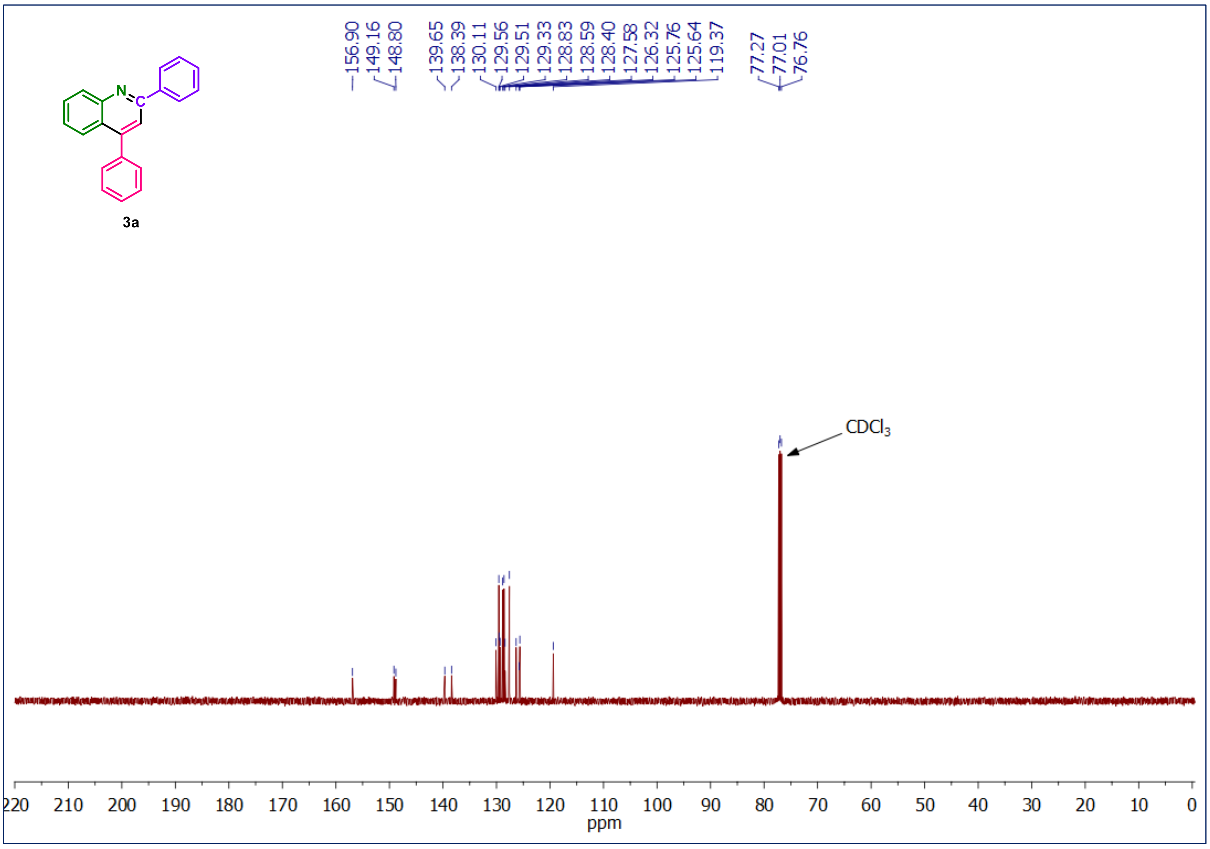

^{1}H NMR (500 MHz, $CDCl_3$) of 4-phenylquinoline (3a')

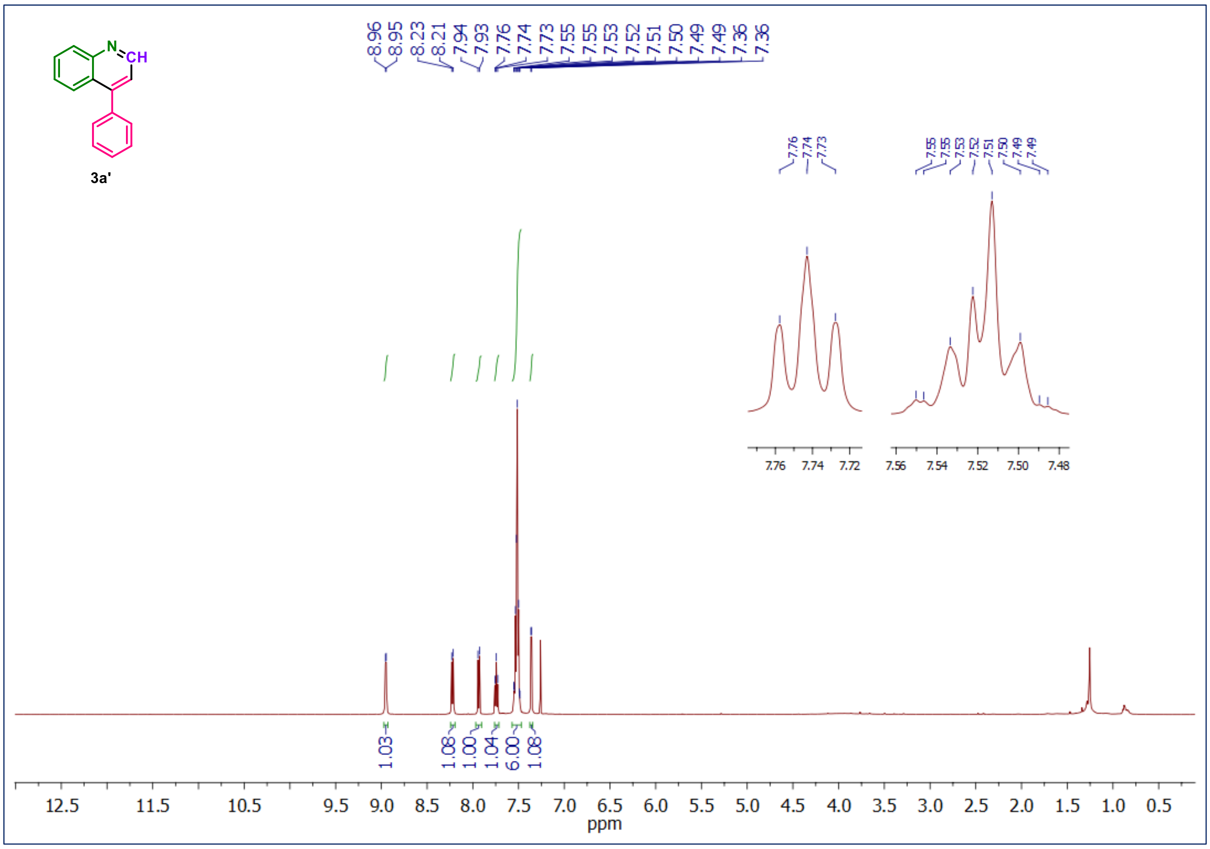

^{13}C{^{1}H} NMR (126 MHz, $CDCl_3$) of 4-phenylquinoline (3a')

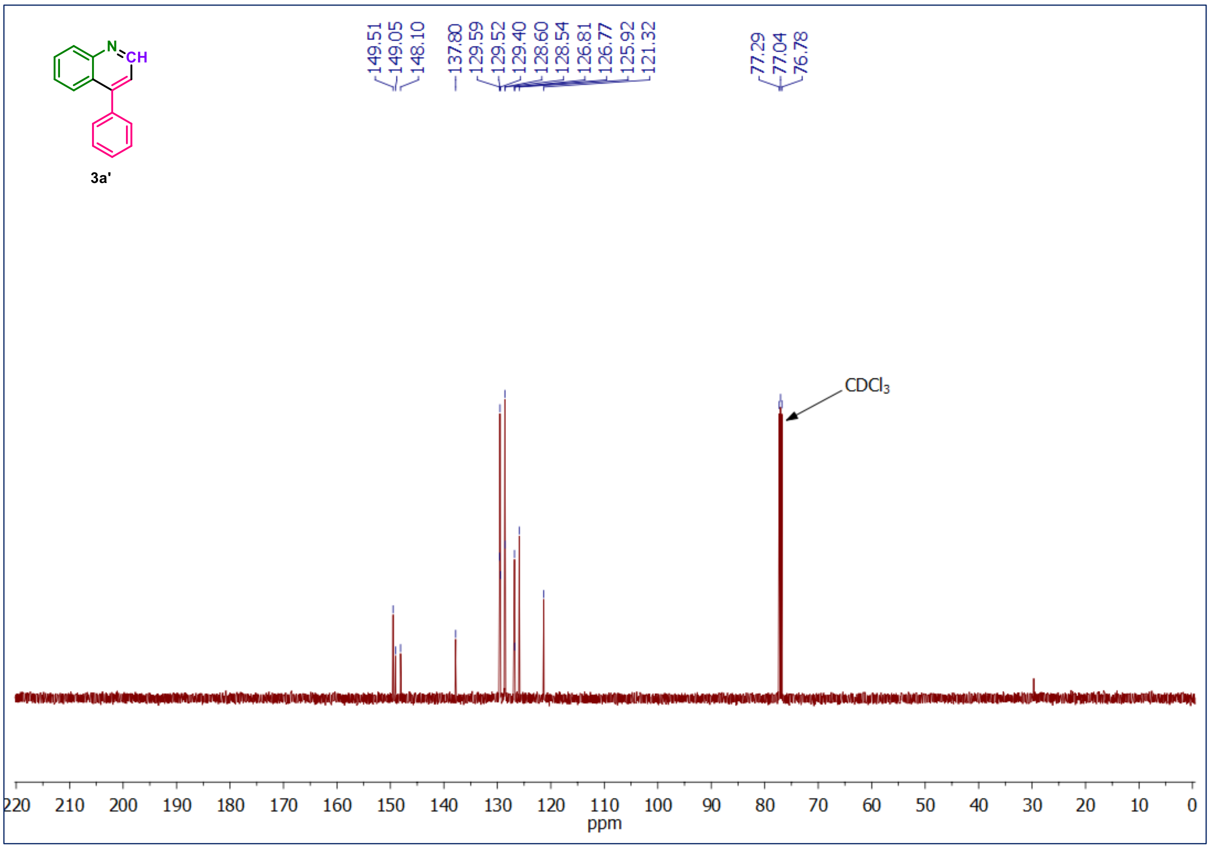

^{1}H NMR (500 MHz, $CDCl_3$) of 6-methyl-2,4-diphenylquinoline (3b)

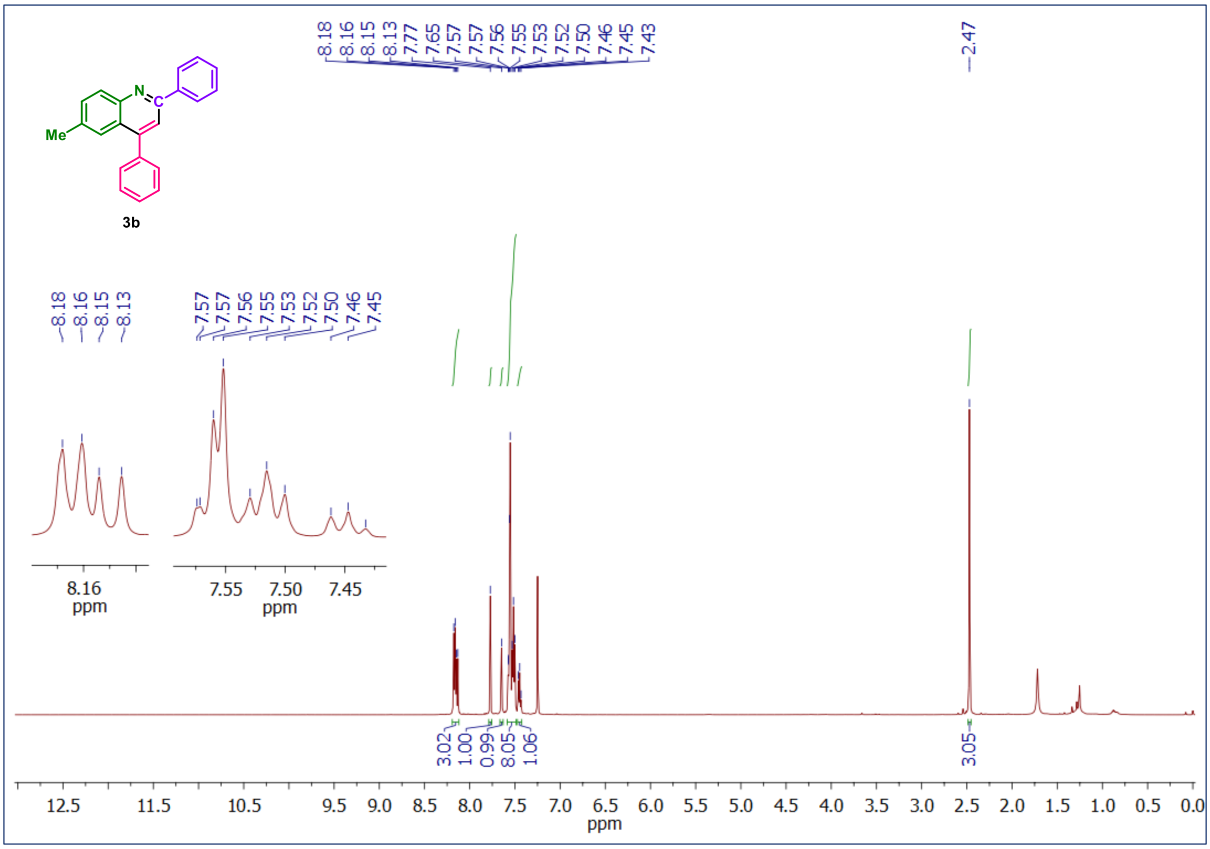

^{13}C{^{1}H} NMR (126 MHz, $CDCl_3$) of 6-methyl-2,4-diphenylquinoline (3b)

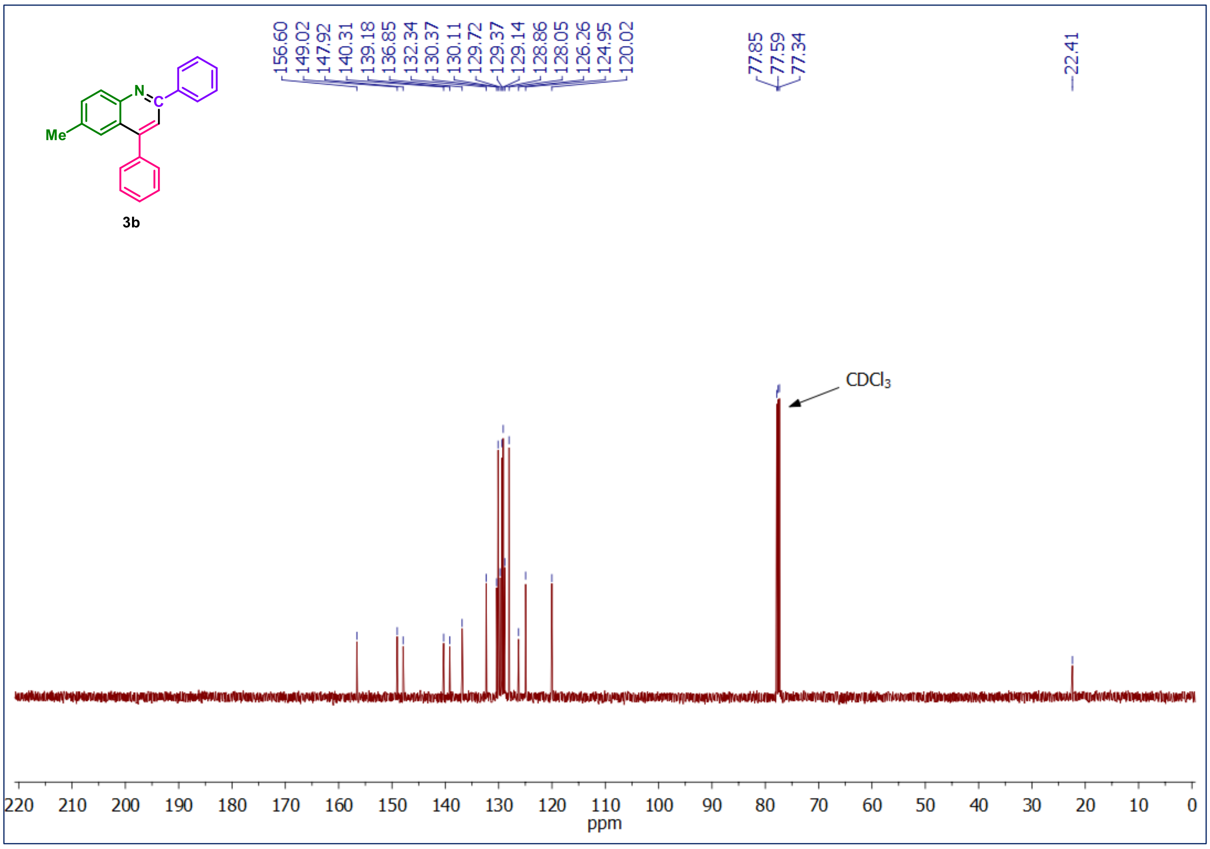

^{1}H NMR (500 MHz, $CDCl_3$) of 6-methyl-4-phenylquinoline (3b')

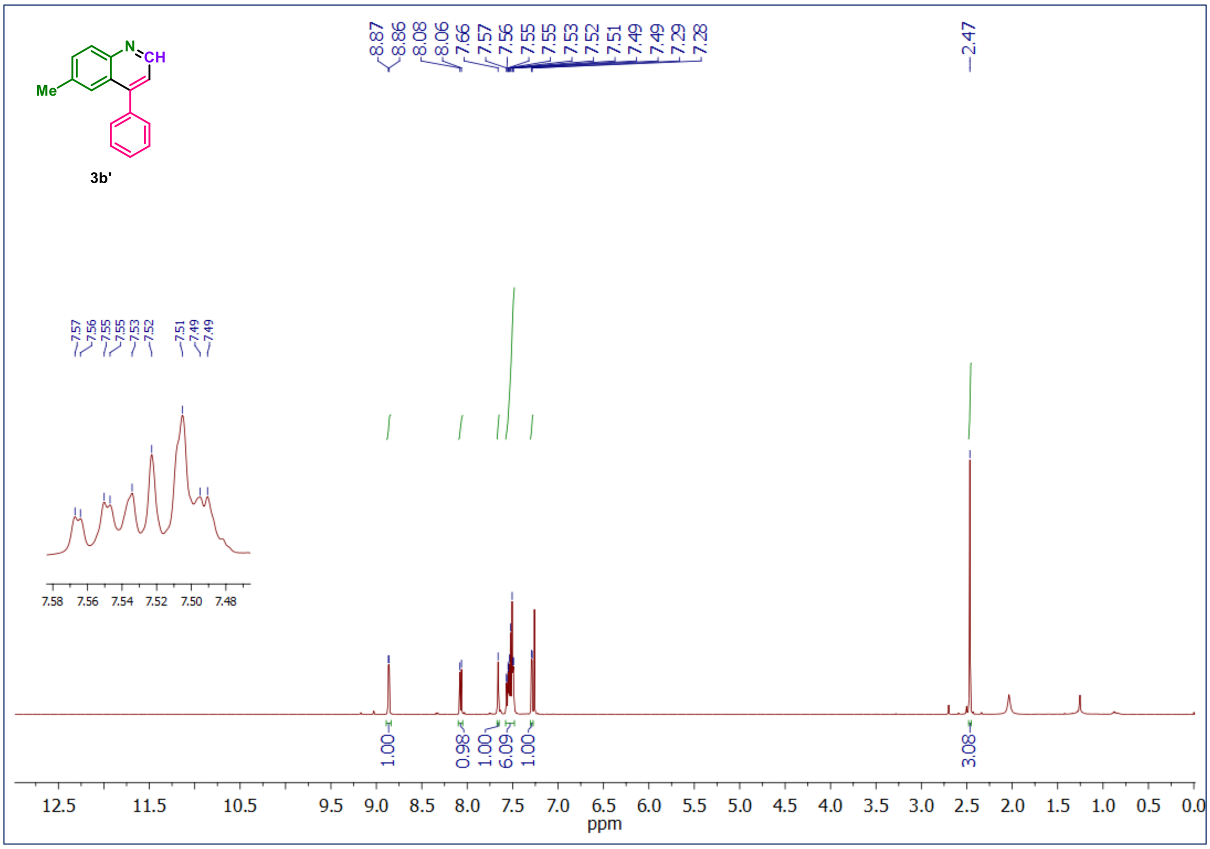

^{13}C{^{1}H} NMR (126 MHz, $CDCl_3$) of 6-methyl-4-phenylquinoline (3b')

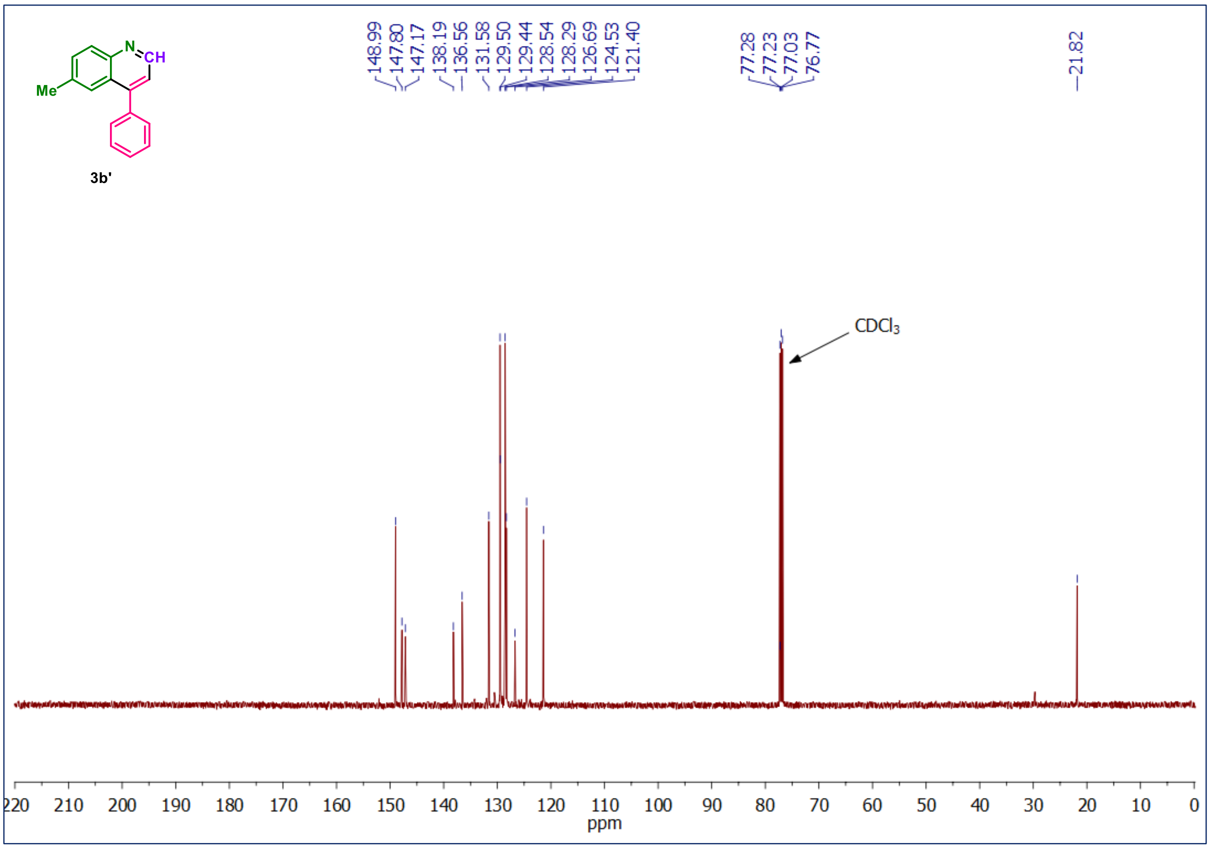

^{1}H NMR (500 MHz, CDCl$_3$) of 6-ethyl-2,4-diphenylquinoline (3c)

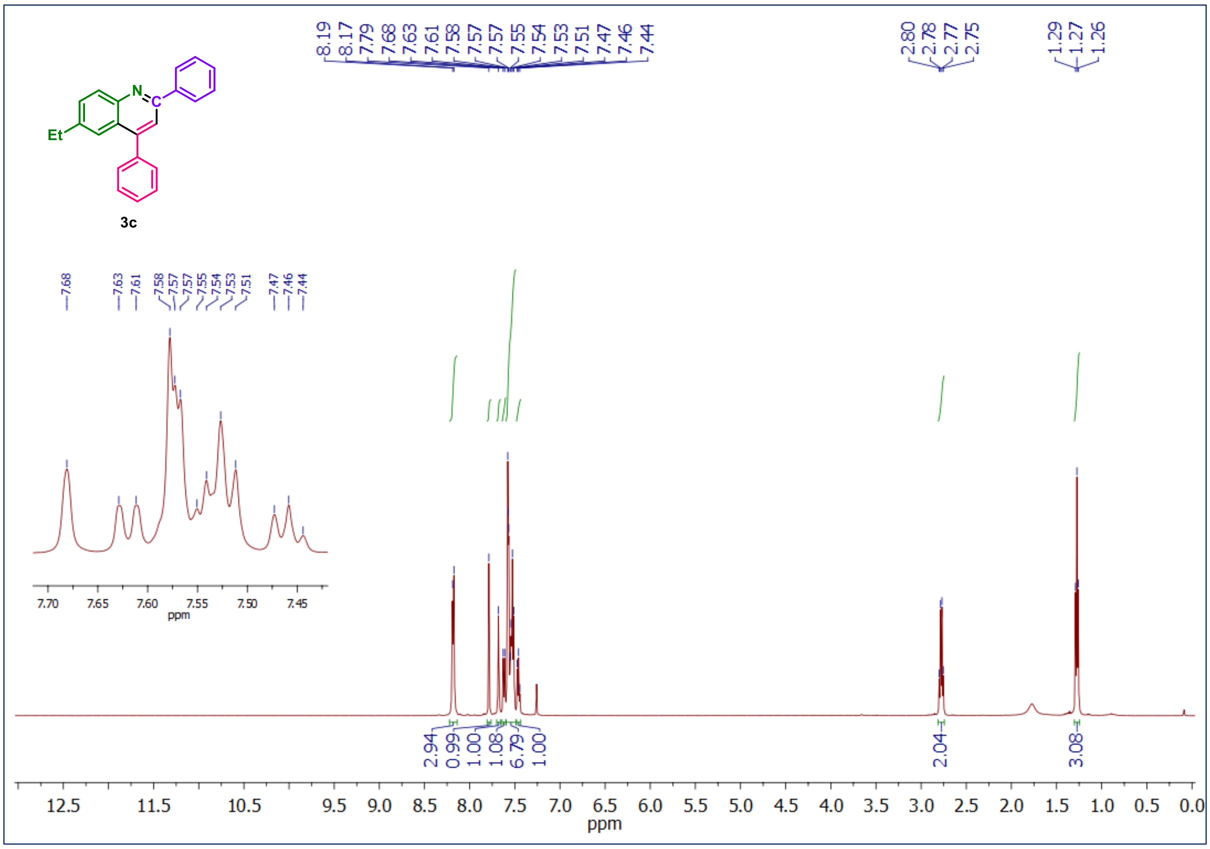

^{13}C{^{1}H} NMR (126 MHz, CDCl$_3$) of 6-ethyl-2,4-diphenylquinoline (3c)

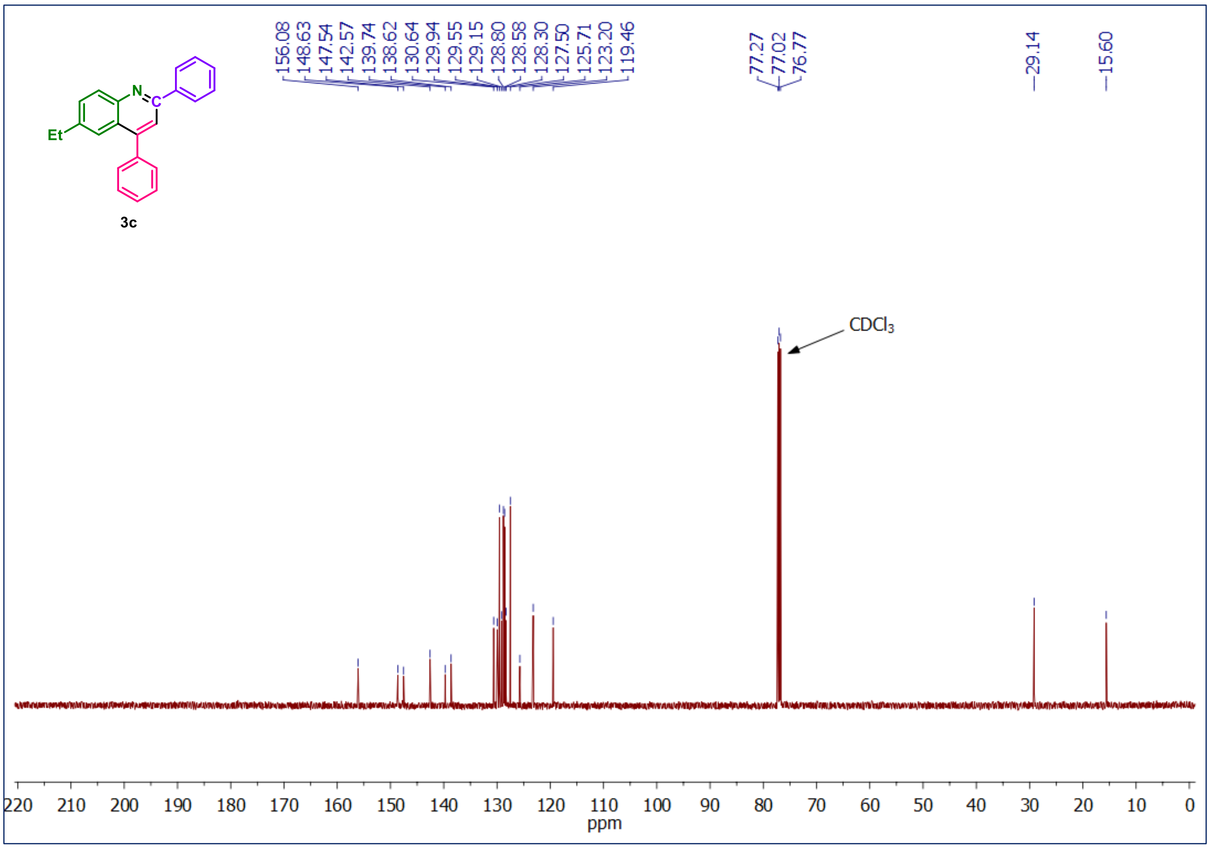

^{1}H NMR (500 MHz, $CDCl_3$) of 6-ethyl-4-phenylquinoline (3c’)

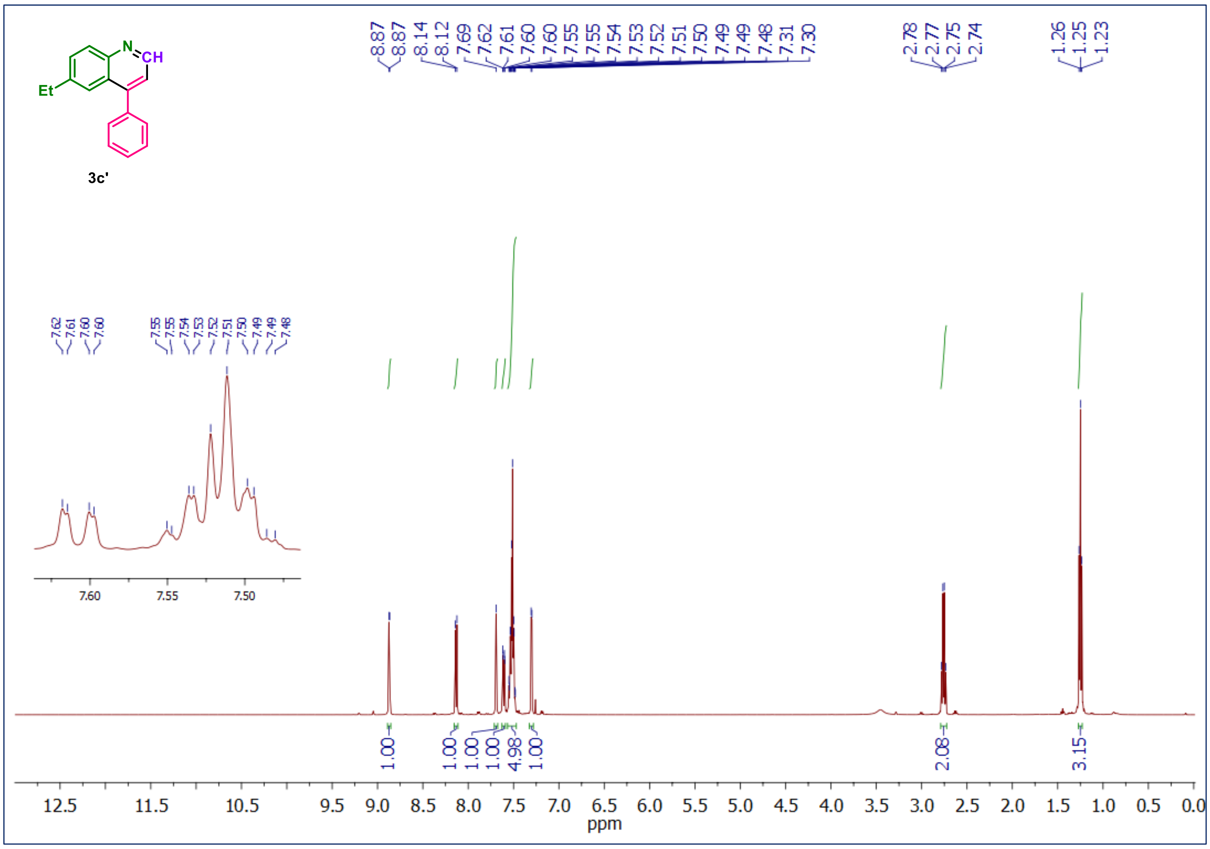

$^{13}C\{^{1}H\}$ NMR (126 MHz, $CDCl_3$) of 6-ethyl-4-phenylquinoline (3c’)

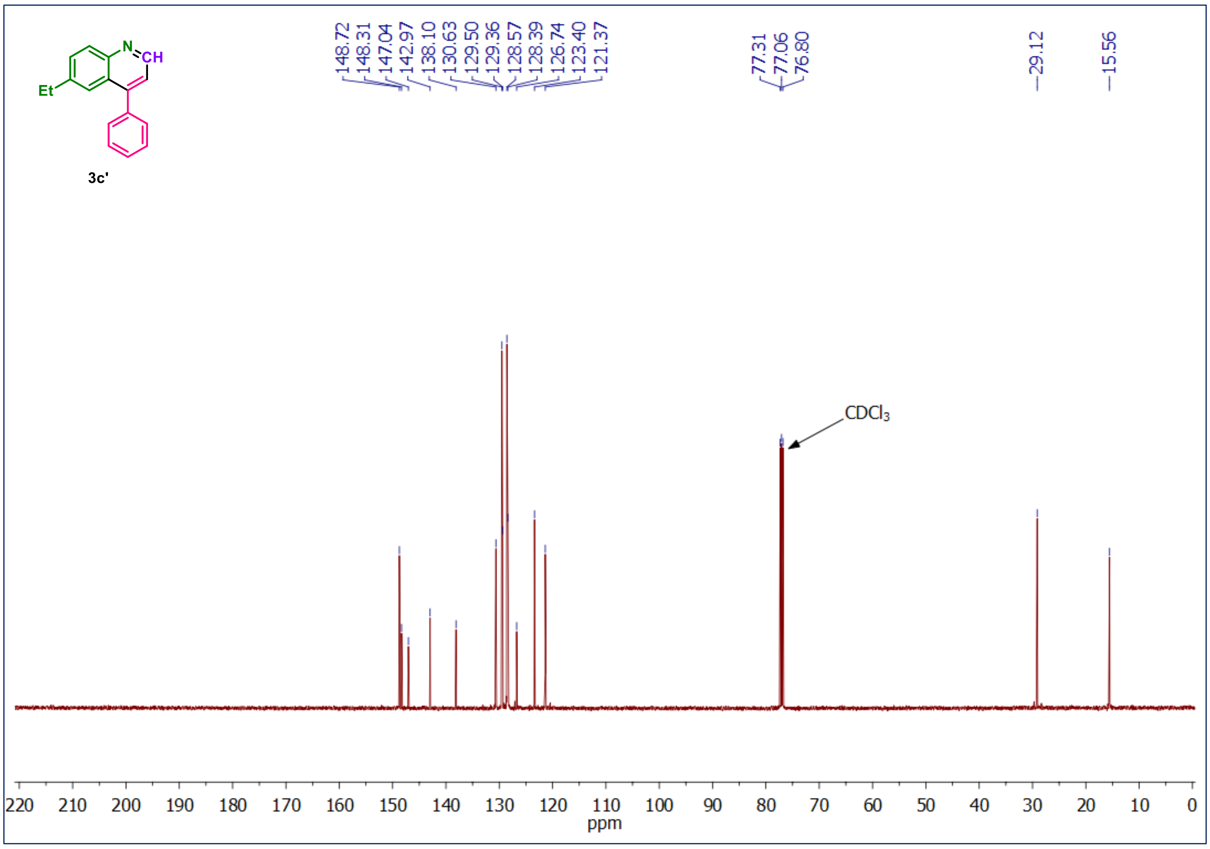

^{1}H NMR (500 MHz, CDCl$_3$) of 6-(*tert*-butyl)-2,4-diphenylquinoline (3d)

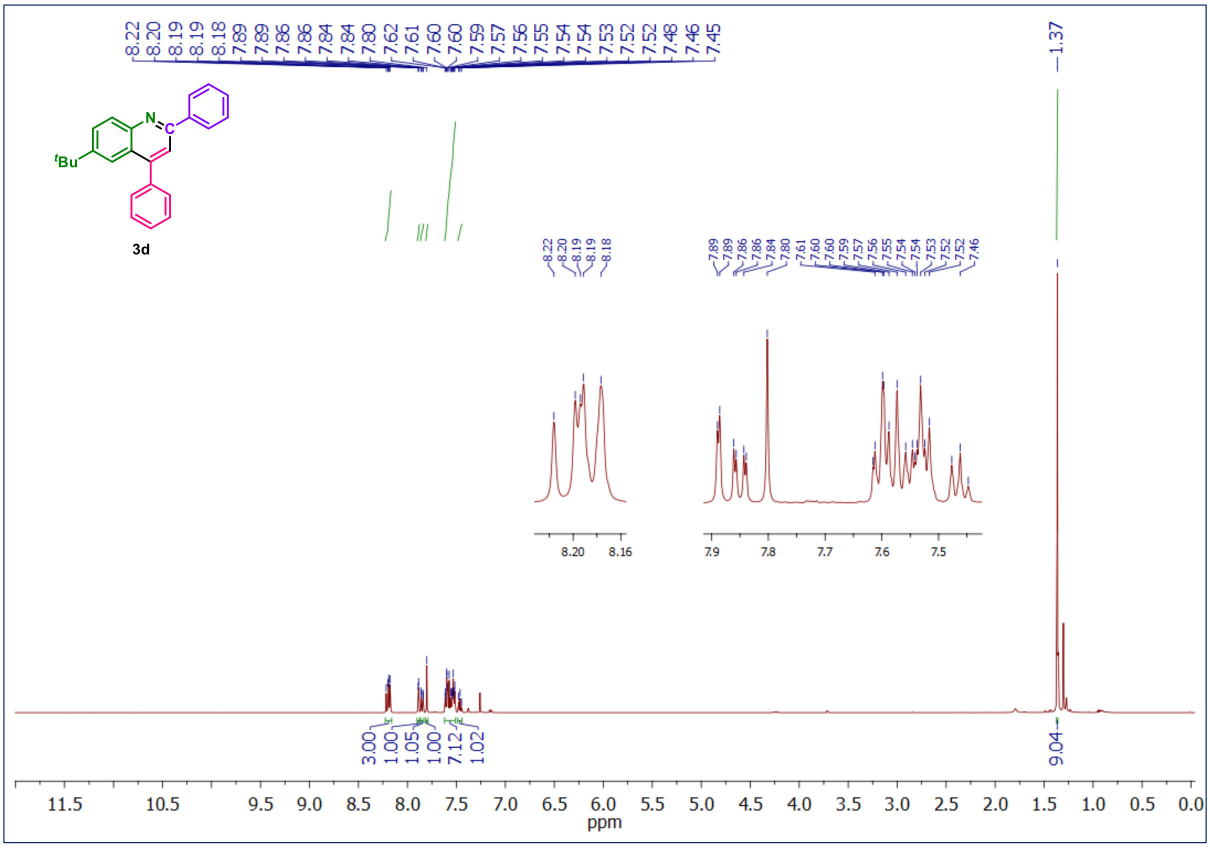

^{13}C{^{1}H} NMR (126 MHz, CDCl$_3$) of 6-(*tert*-butyl)-2,4-diphenylquinoline (3d)

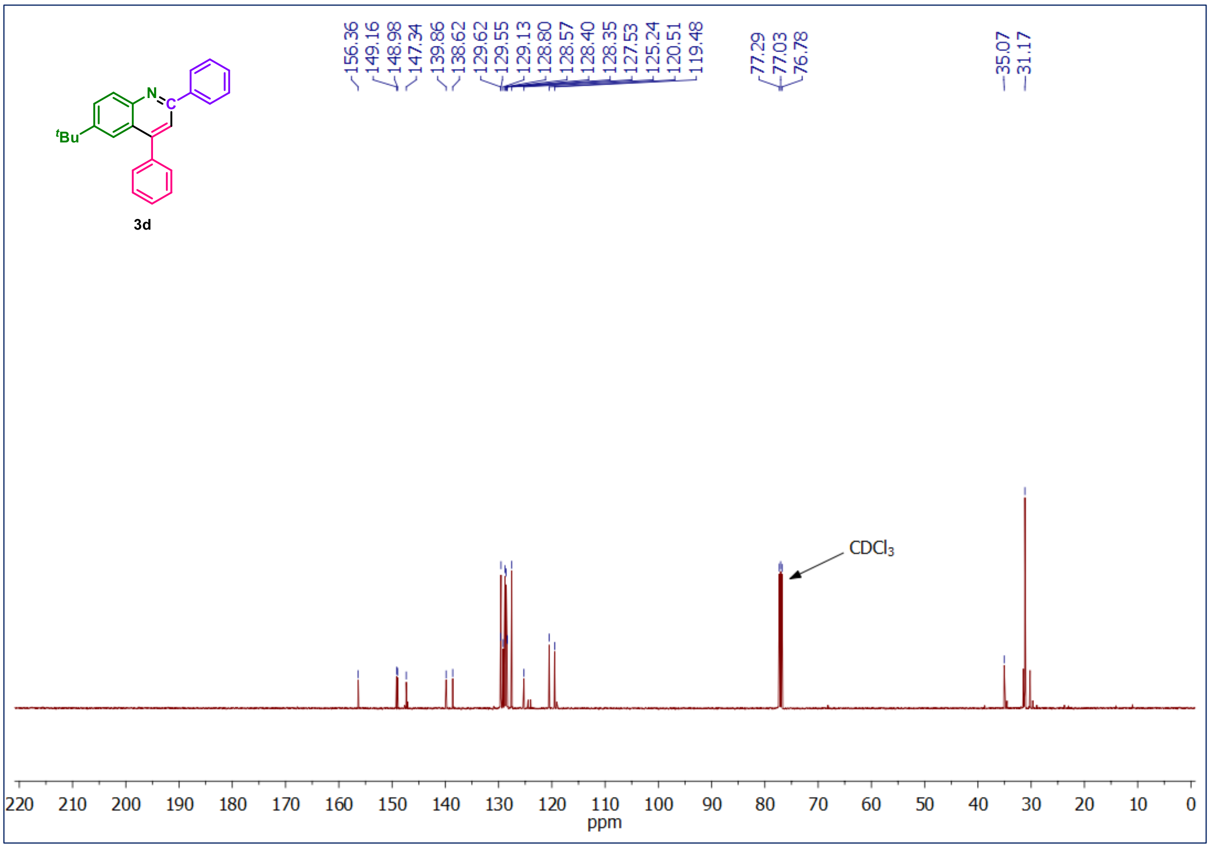

^{1}H NMR (500 MHz, $CDCl_3$) of 6-(*tert*-butyl)-4-phenylquinoline (3d’)

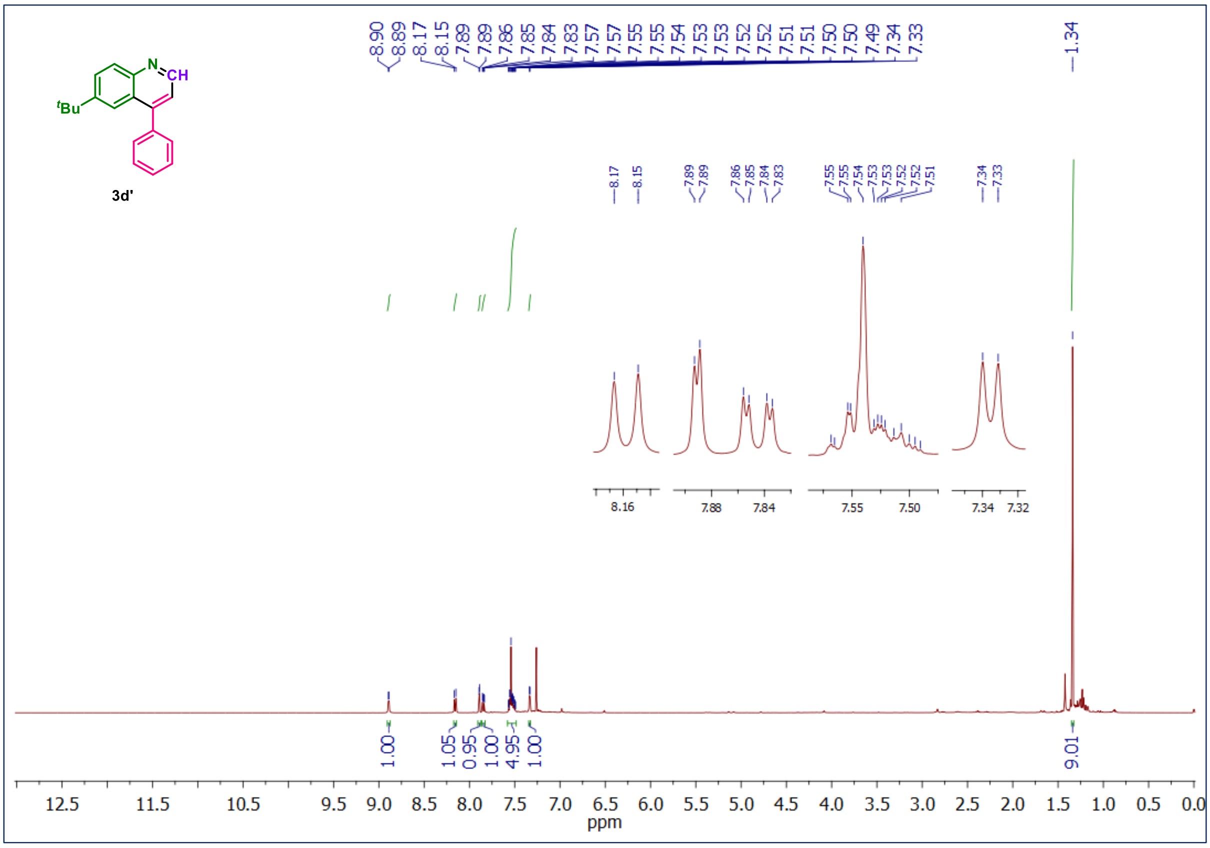

$^{13}C\{^{1}H\}$ NMR (126 MHz, $CDCl_3$) of 6-(*tert*-butyl)-4-phenylquinoline (3d’)

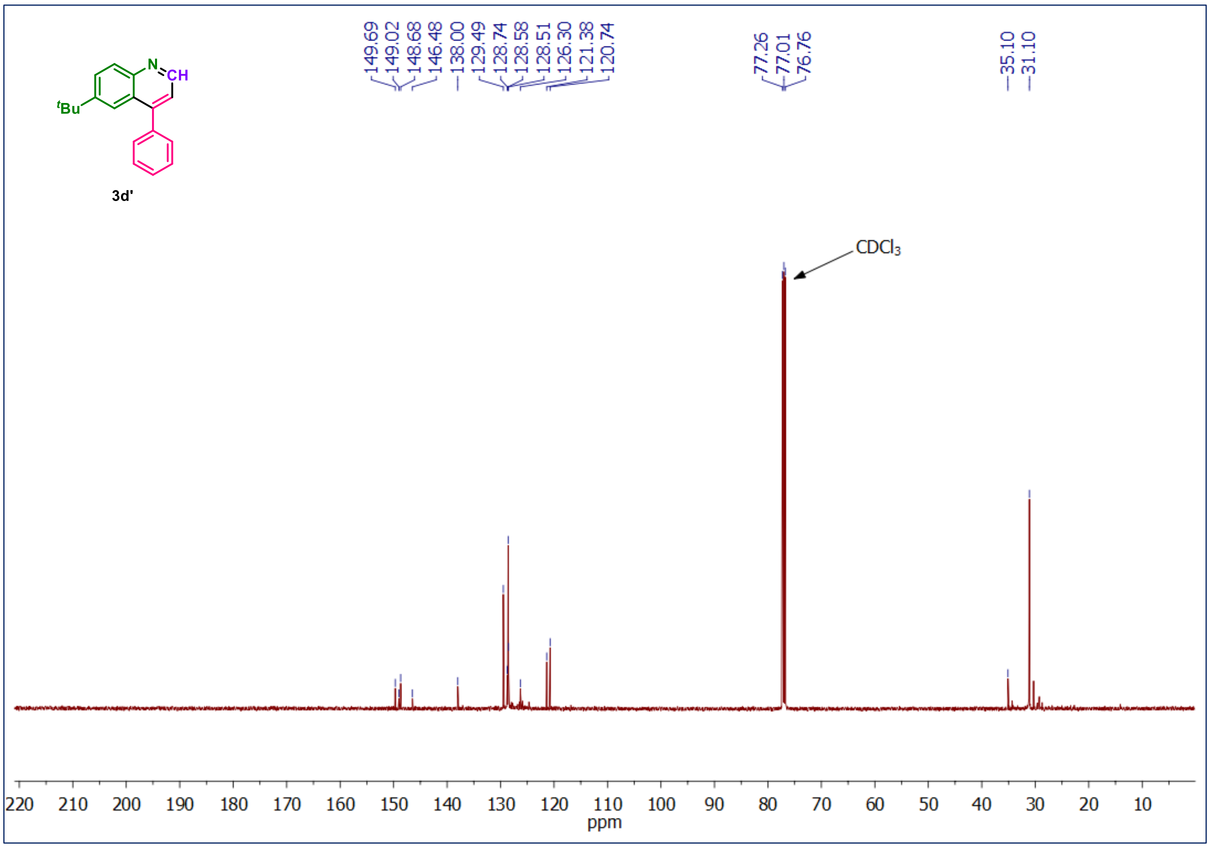

^{1}H NMR (500 MHz, $CDCl_3$) of 2,4,6-triphenylquinoline (3e)

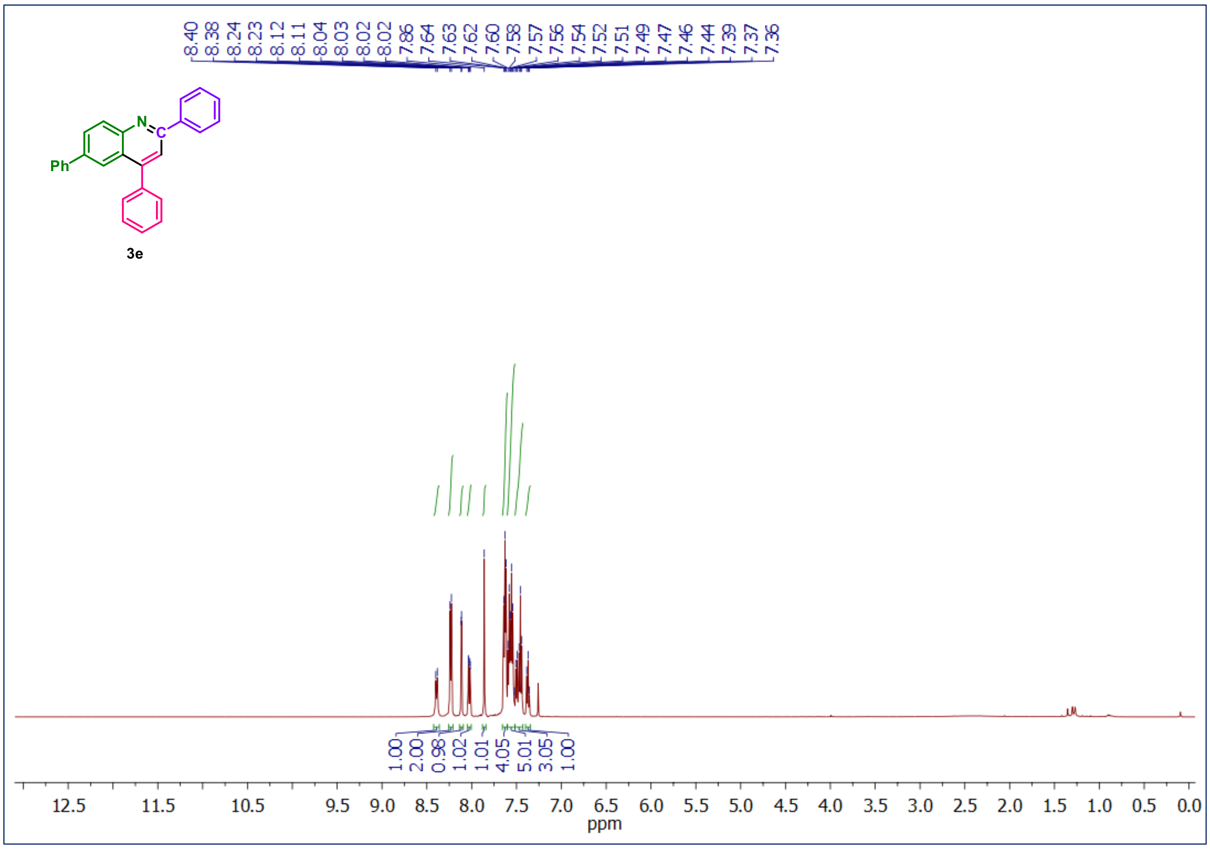

$^{13}C\{^{1}H\}$ NMR (126 MHz, $CDCl_3$) of 2,4,6-triphenylquinoline (3e)

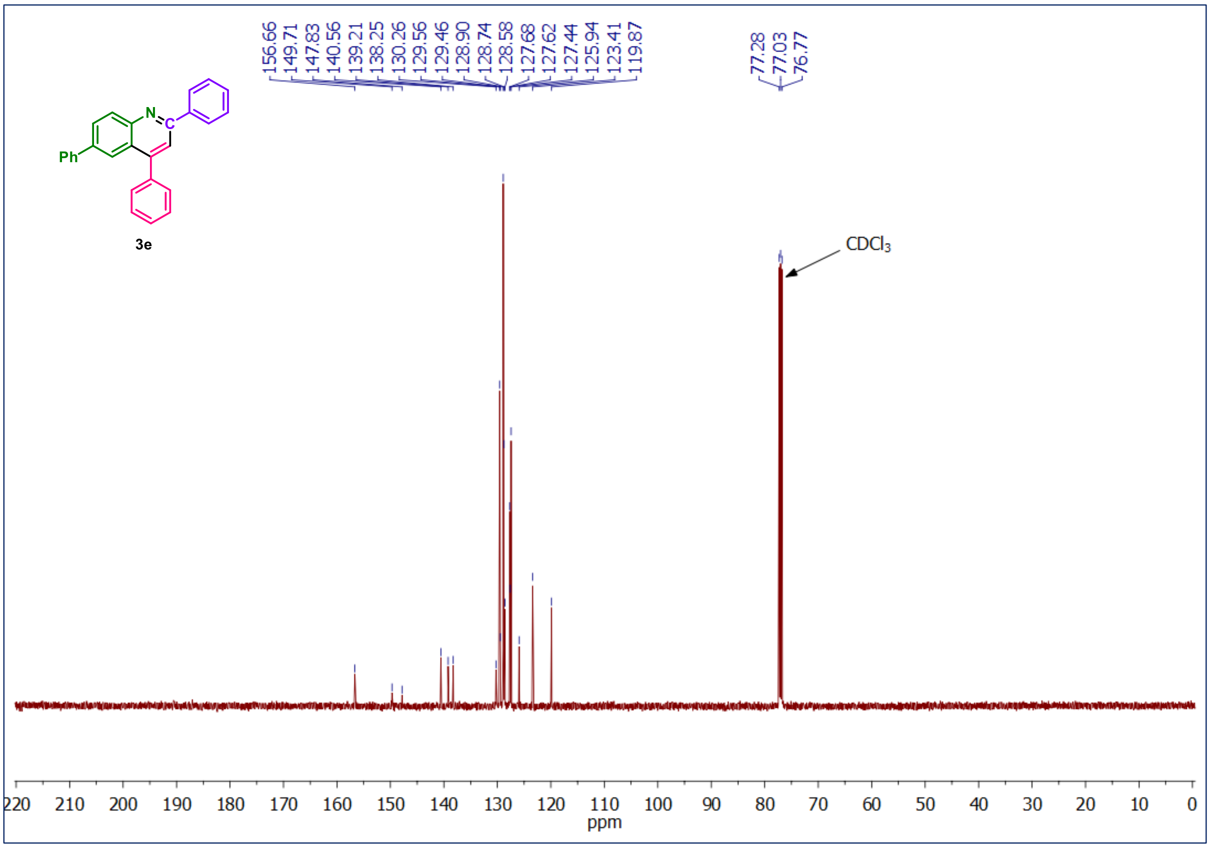

^{1}H NMR (500 MHz, $CDCl_3$) of 4,6-diphenylquinoline (3e')

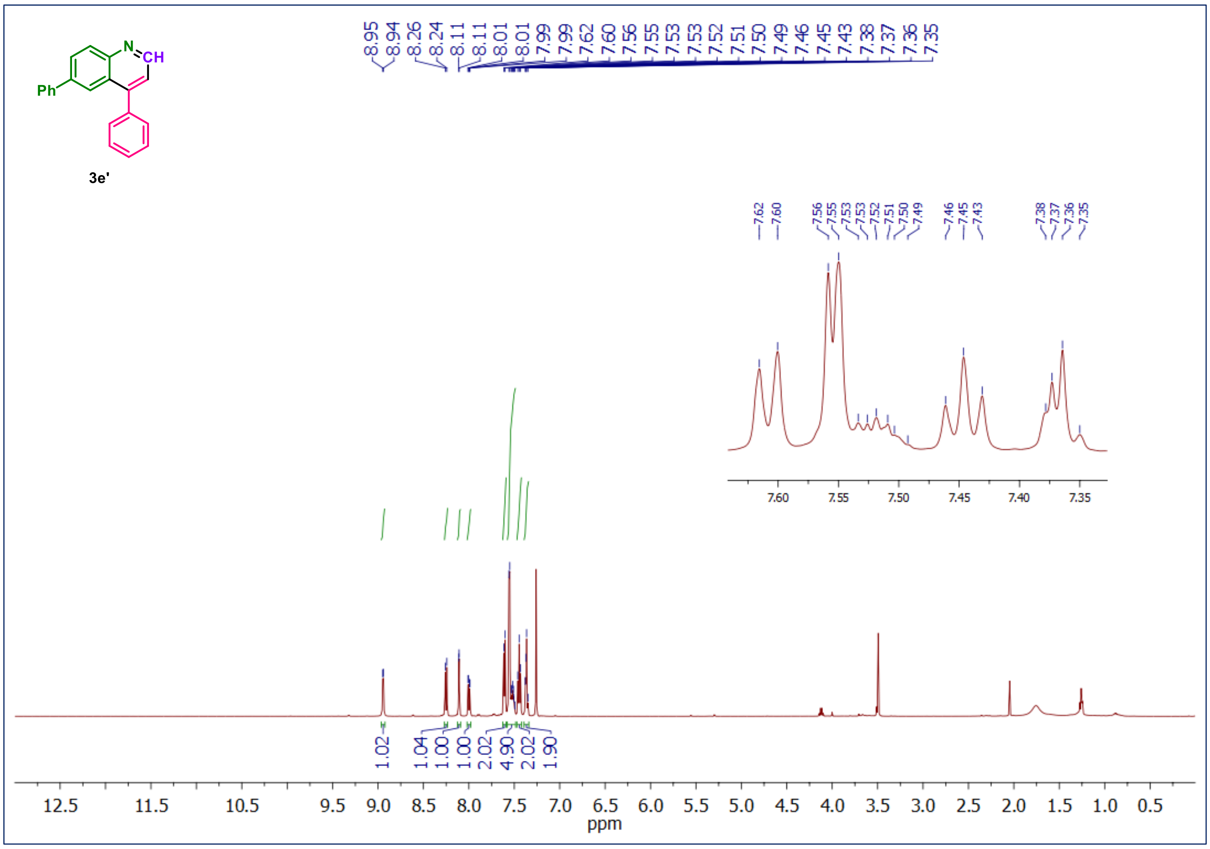

$^{13}C\{^{1}H\}$ NMR (126 MHz, $CDCl_3$) of 4,6-diphenylquinoline (3e')

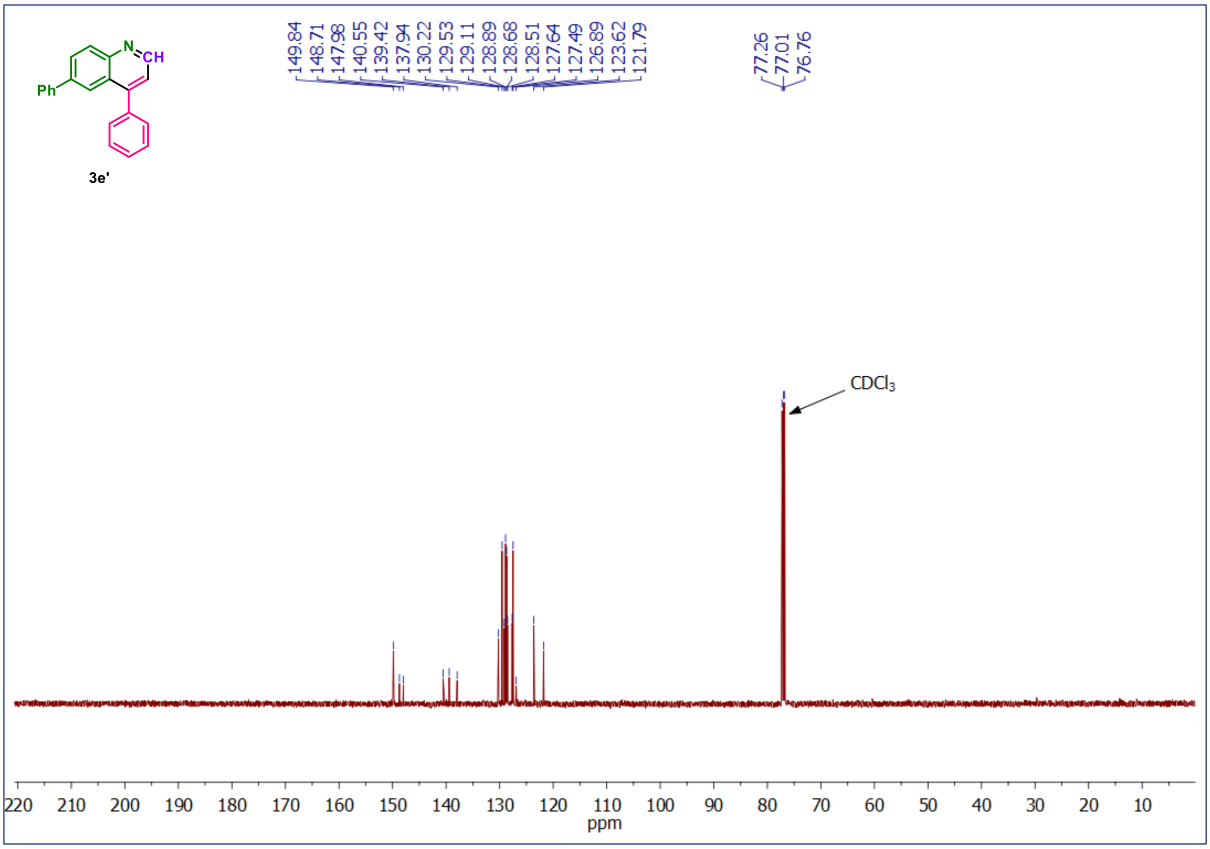

^{1}H NMR (500 MHz, $CDCl_3$) of 6-chloro-2,4-diphenylquinoline (3f)

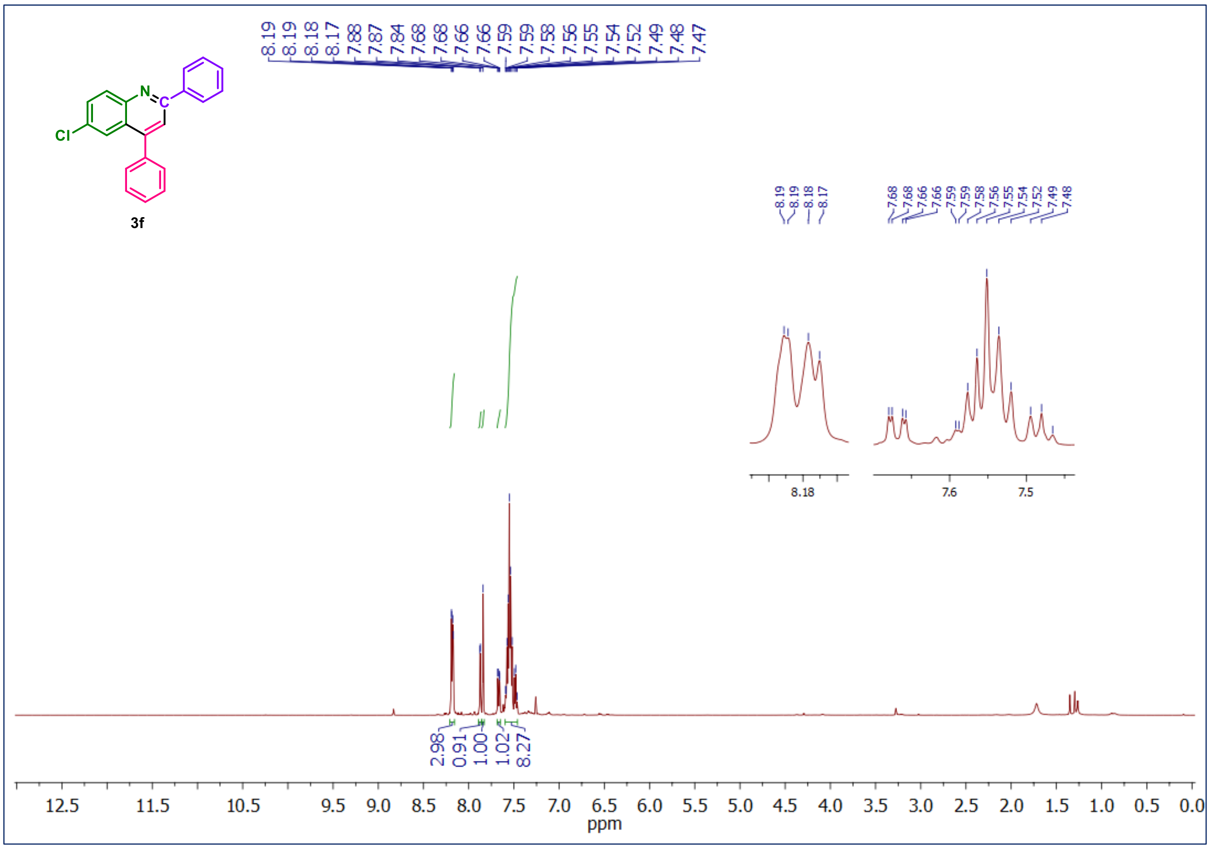

^{13}C{^{1}H} NMR (126 MHz, $CDCl_3$) of 6-chloro-2,4-diphenylquinoline (3f)

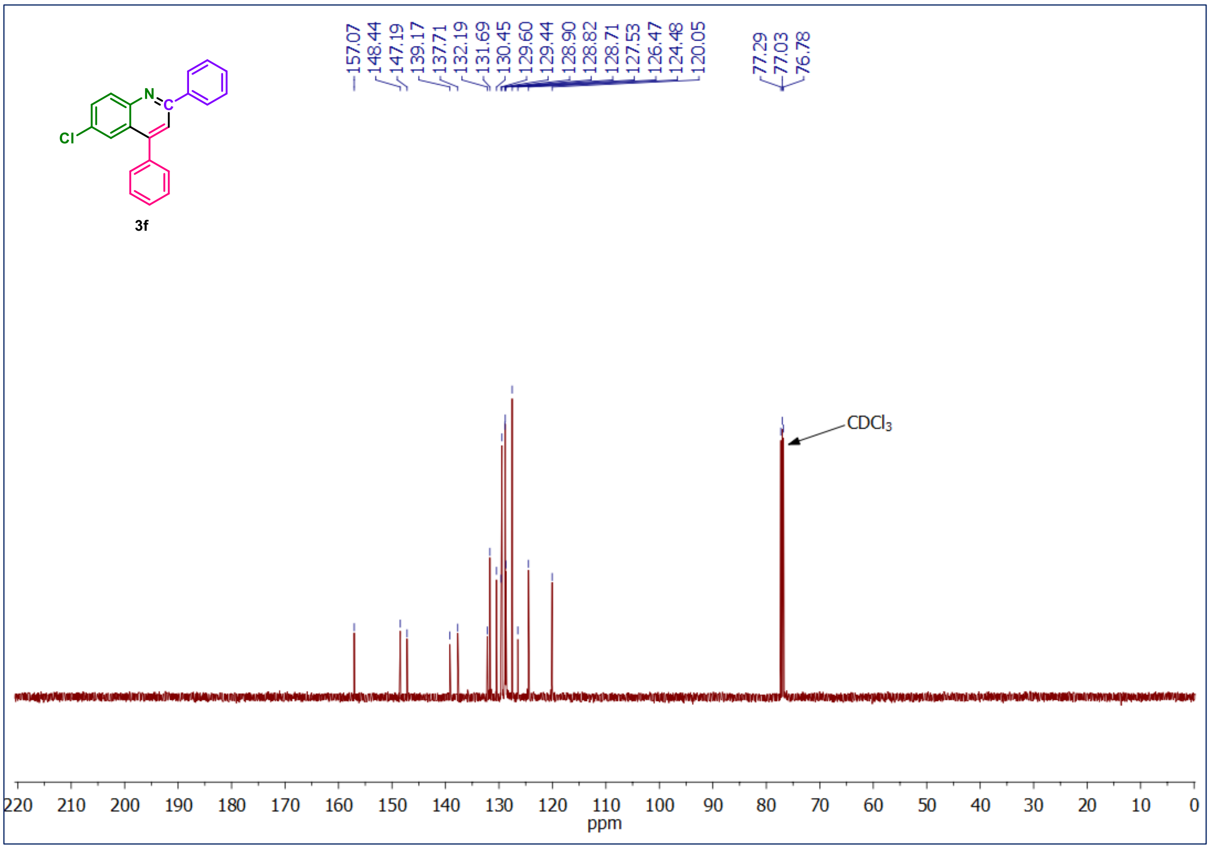

^{1}H NMR (500 MHz, $CDCl_3$) of 6-chloro-4-phenylquinoline (3f')

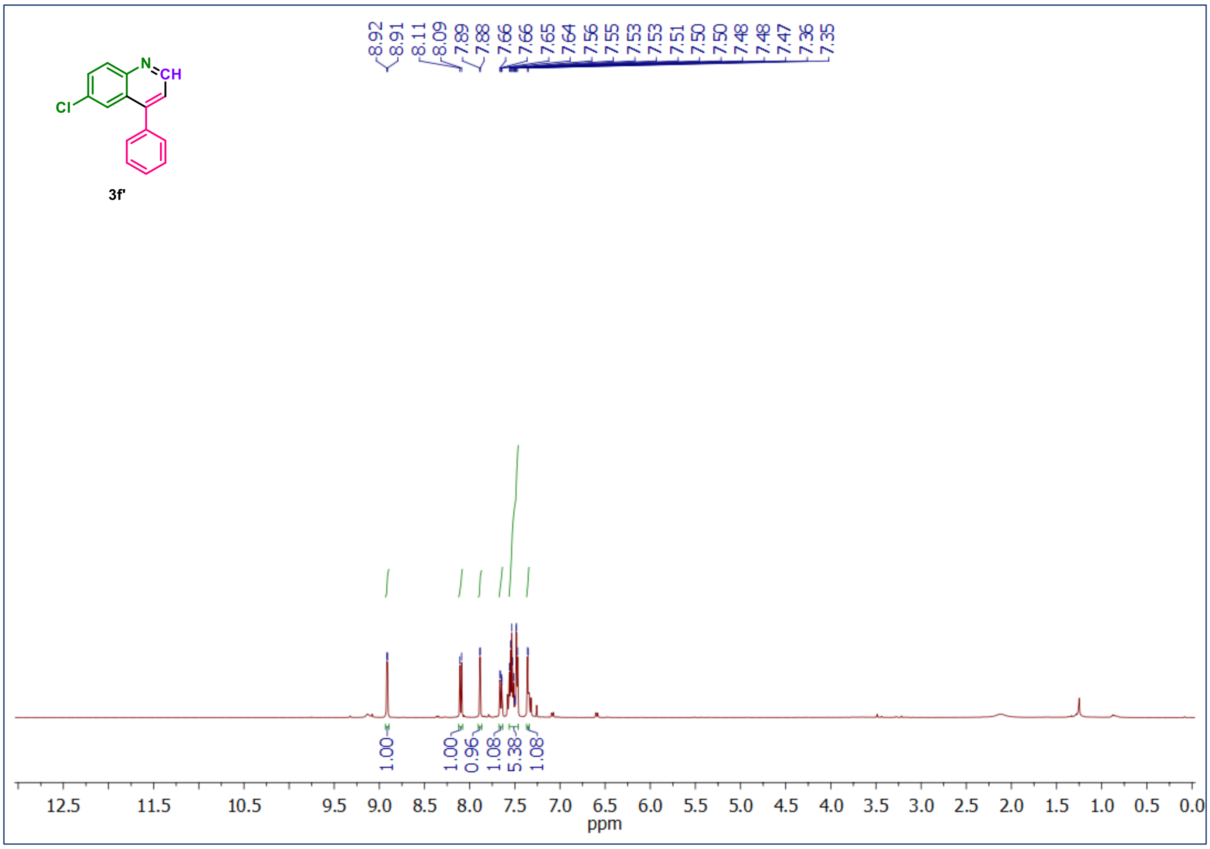

$^{13}C\{^{1}H\}$ NMR (126 MHz, $CDCl_3$) of 6-chloro-4-phenylquinoline (3f')

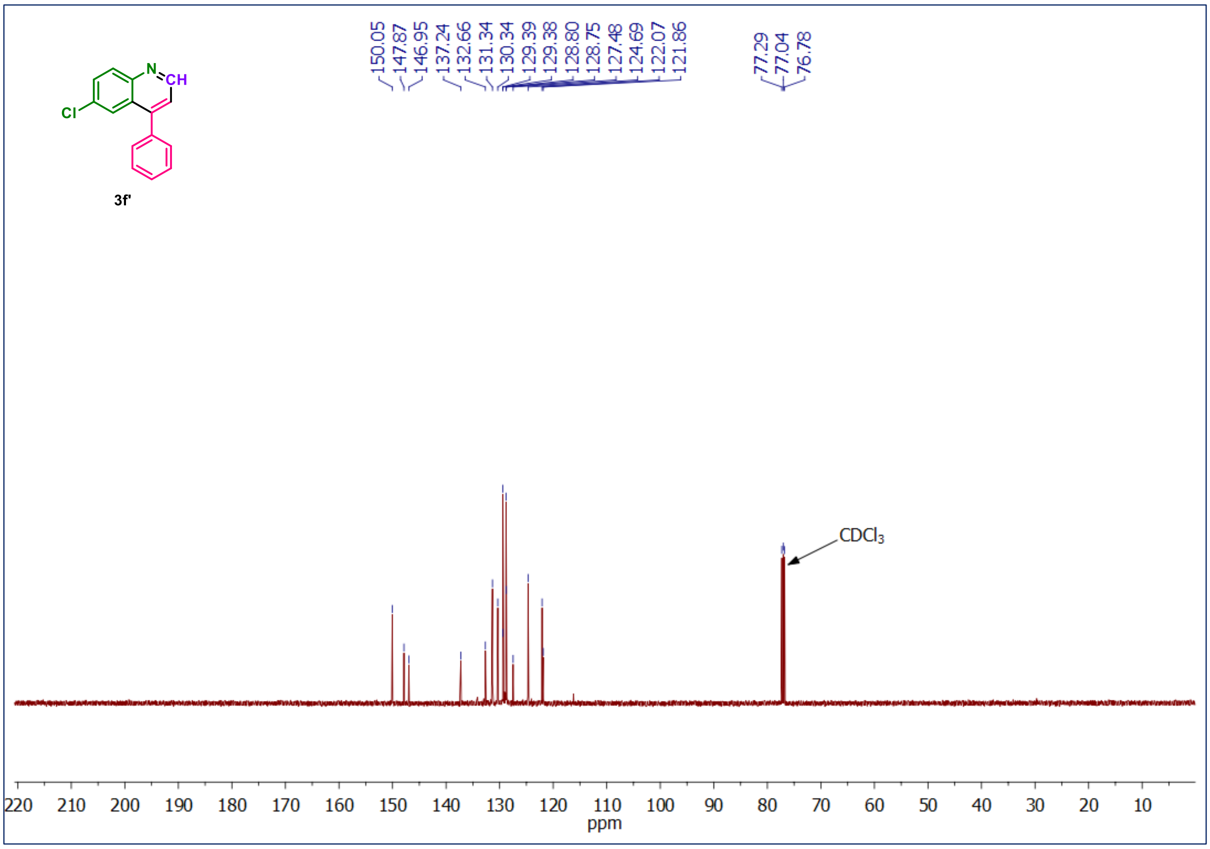

^{1}H NMR (500 MHz, $CDCl_3$) of 6-bromo-2,4-diphenylquinoline (3g)

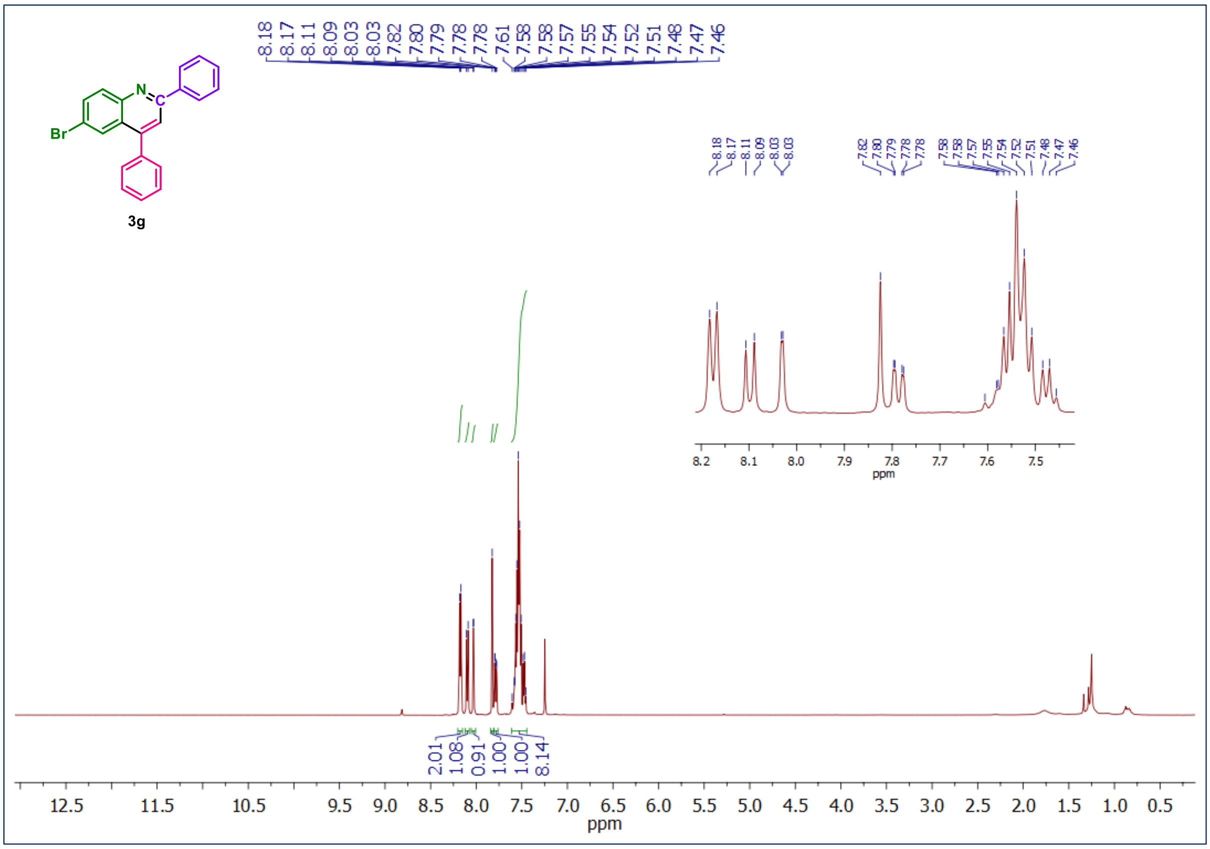

$^{13}C\{^{1}H\}$ NMR (126 MHz, $CDCl_3$) of 6-bromo-2,4-diphenylquinoline (3g)

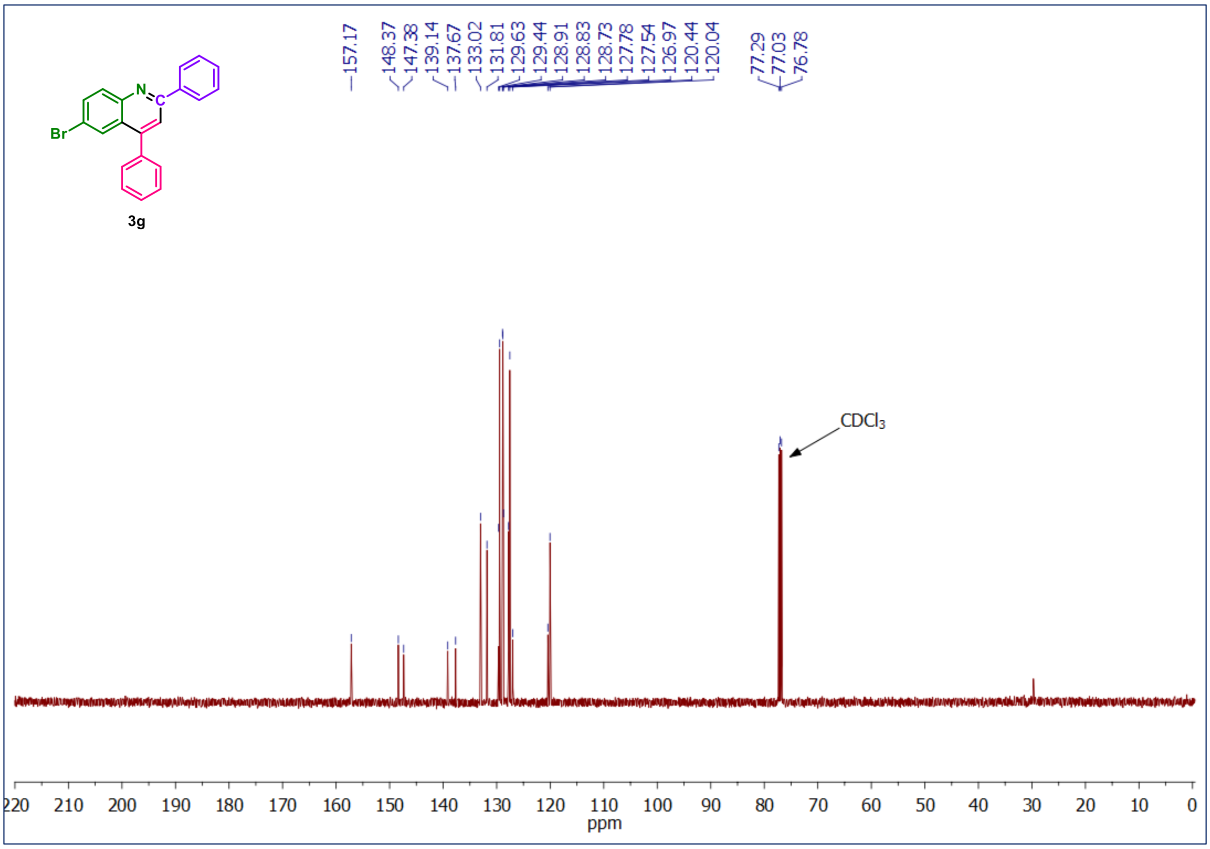

^{1}H NMR (500 MHz, CDCl3) of 6-bromo-4-phenylquinoline (3g')

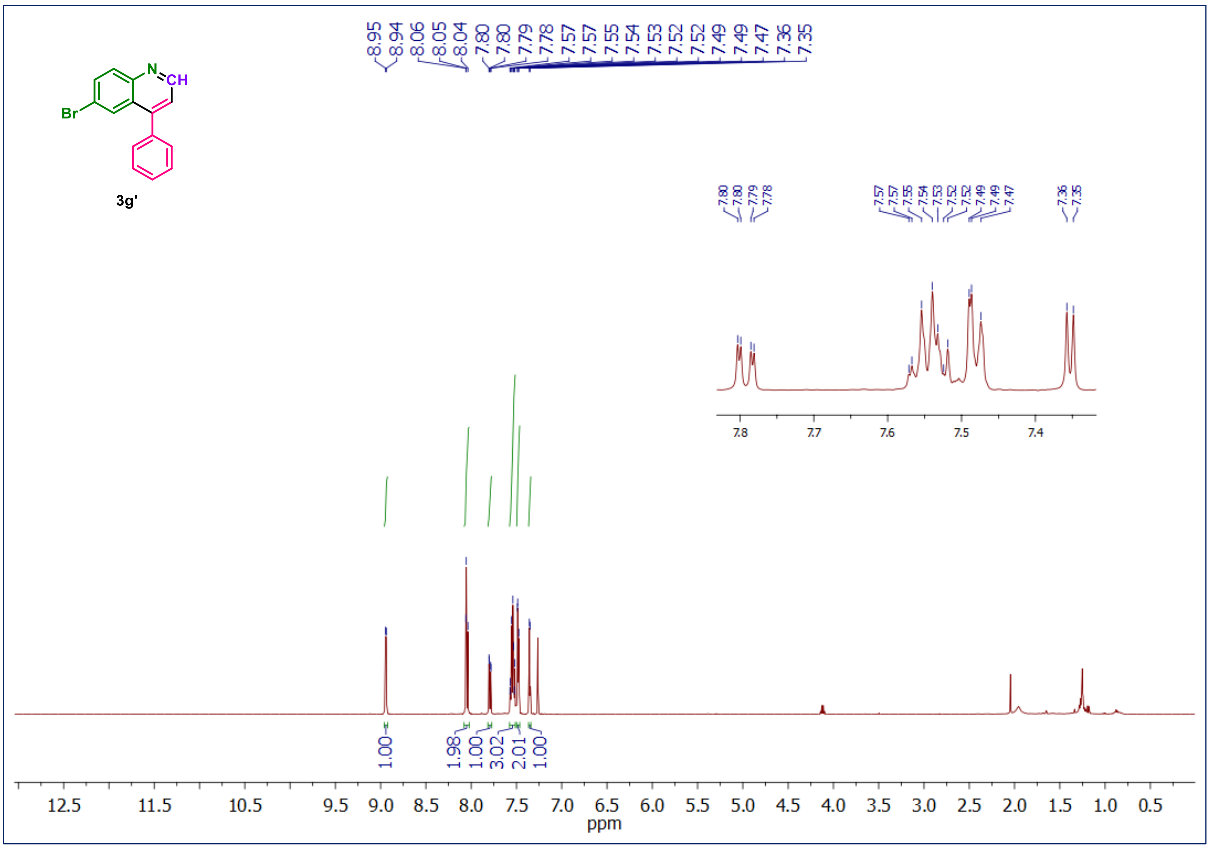

^{13}C{^{1}H} NMR (126 MHz, CDCl3) of 6-bromo-4-phenylquinoline (3g')

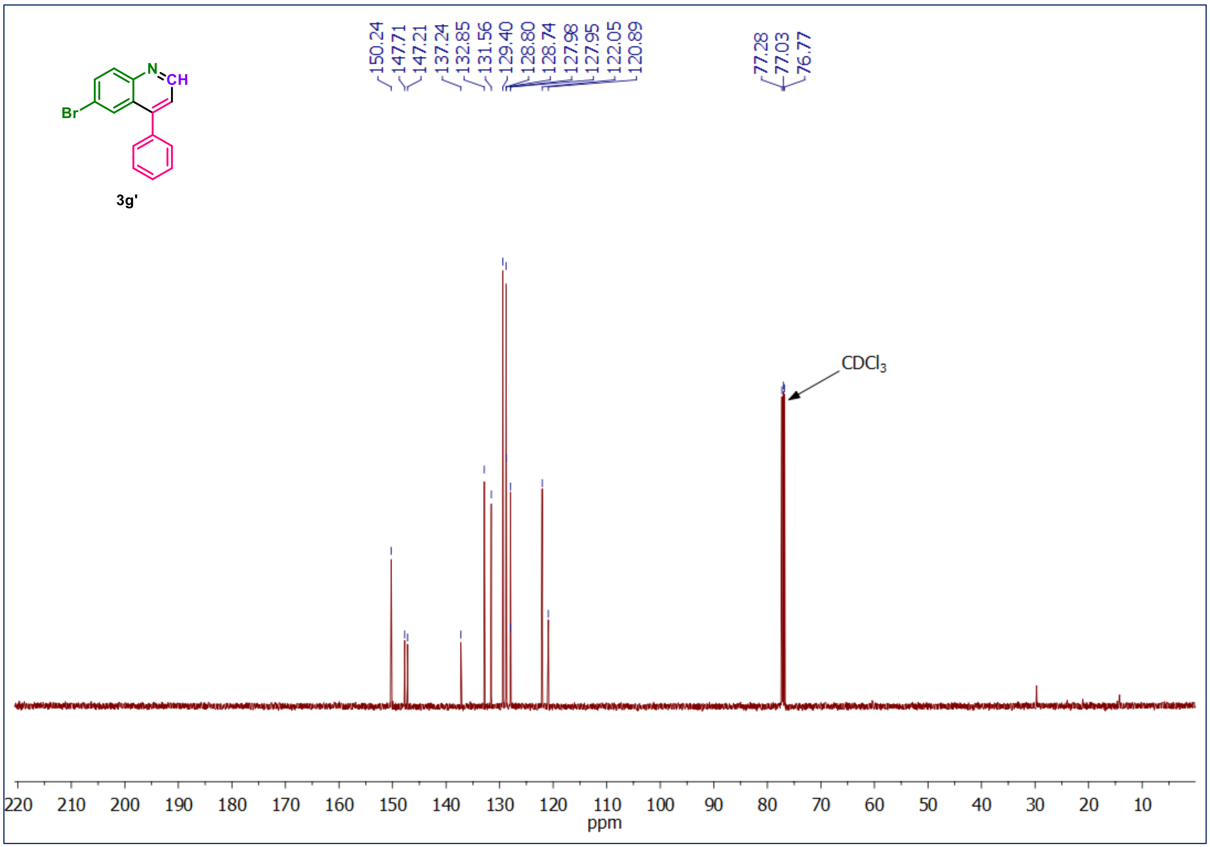

^{1}H NMR (500 MHz, $CDCl_3$) of 6-fluoro-2,4-diphenylquinoline (3h)

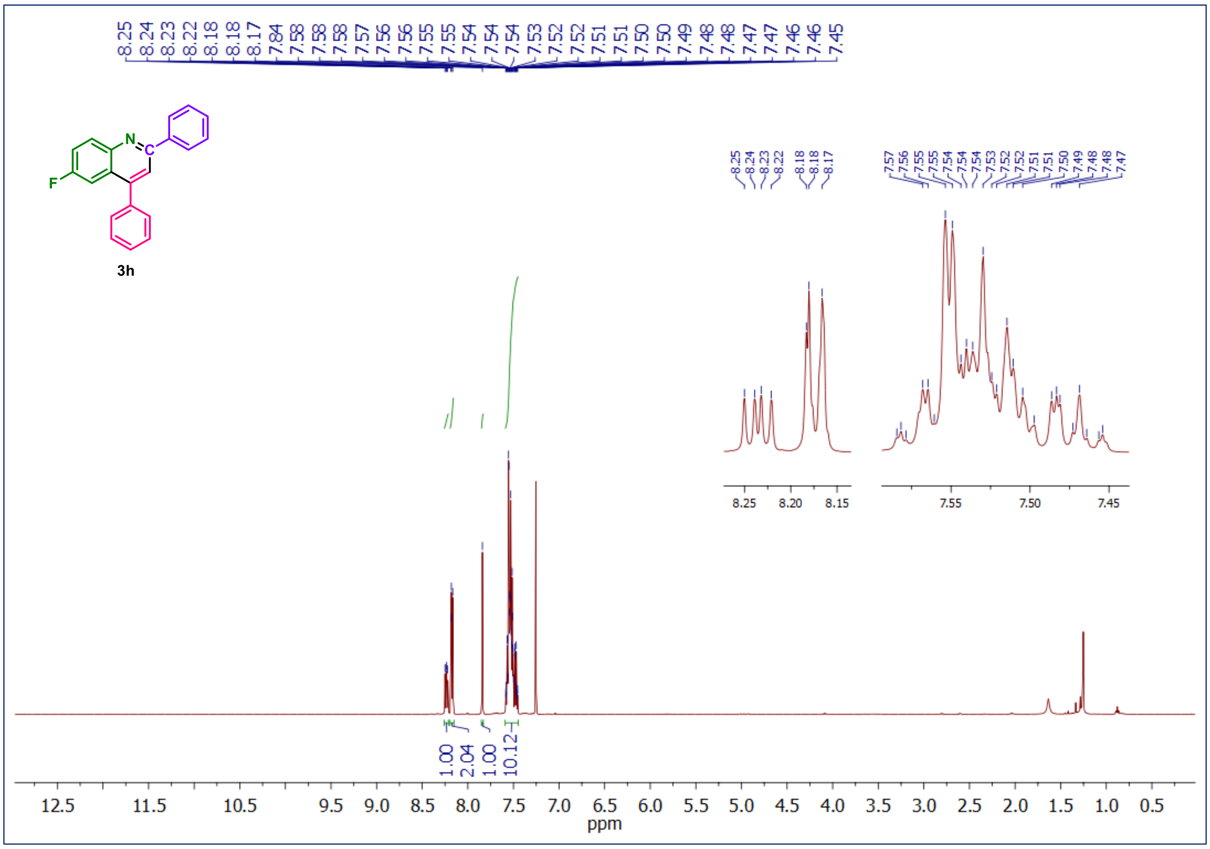

^{13}C{^{1}H} NMR (126 MHz, $CDCl_3$) of 6-fluoro-2,4-diphenylquinoline (3h)

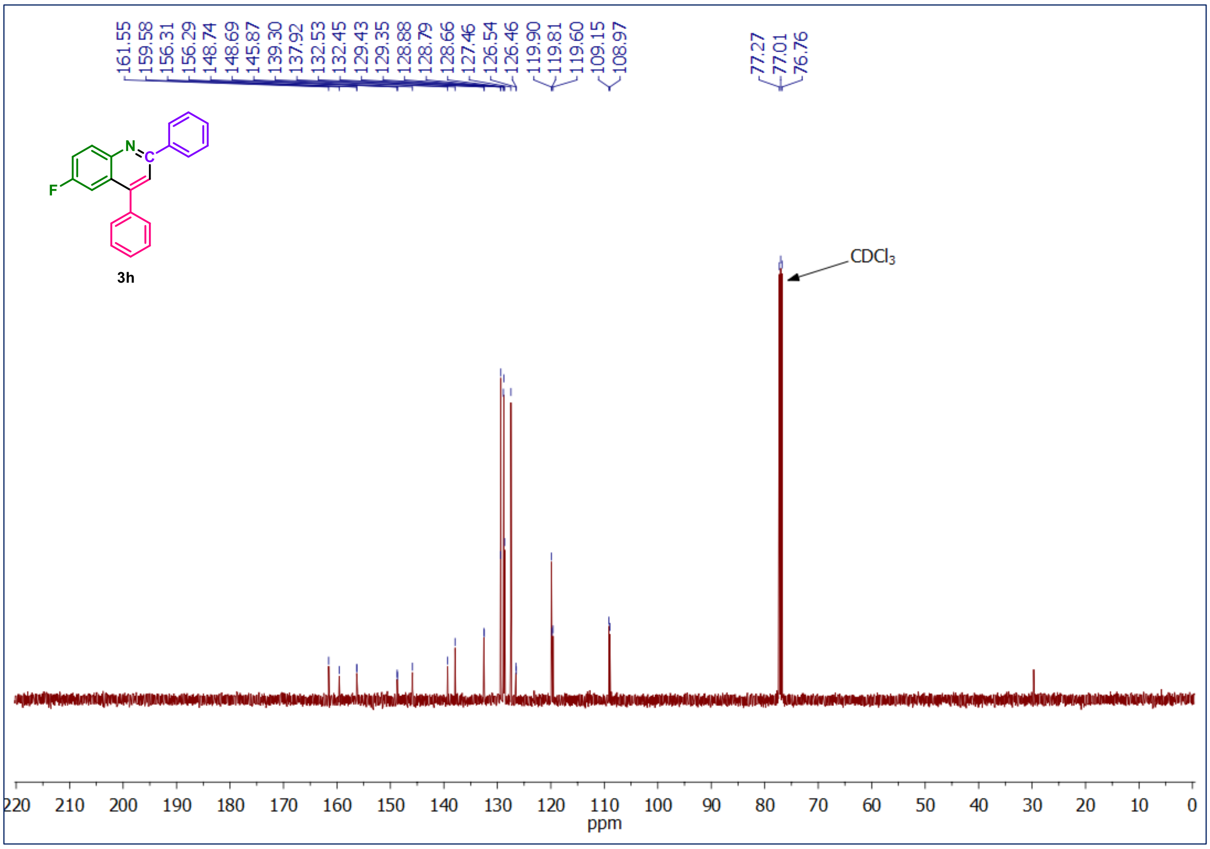

^{1}H NMR (500 MHz, CDCl3) of 6-fluoro-4-phenylquinoline (3h’)

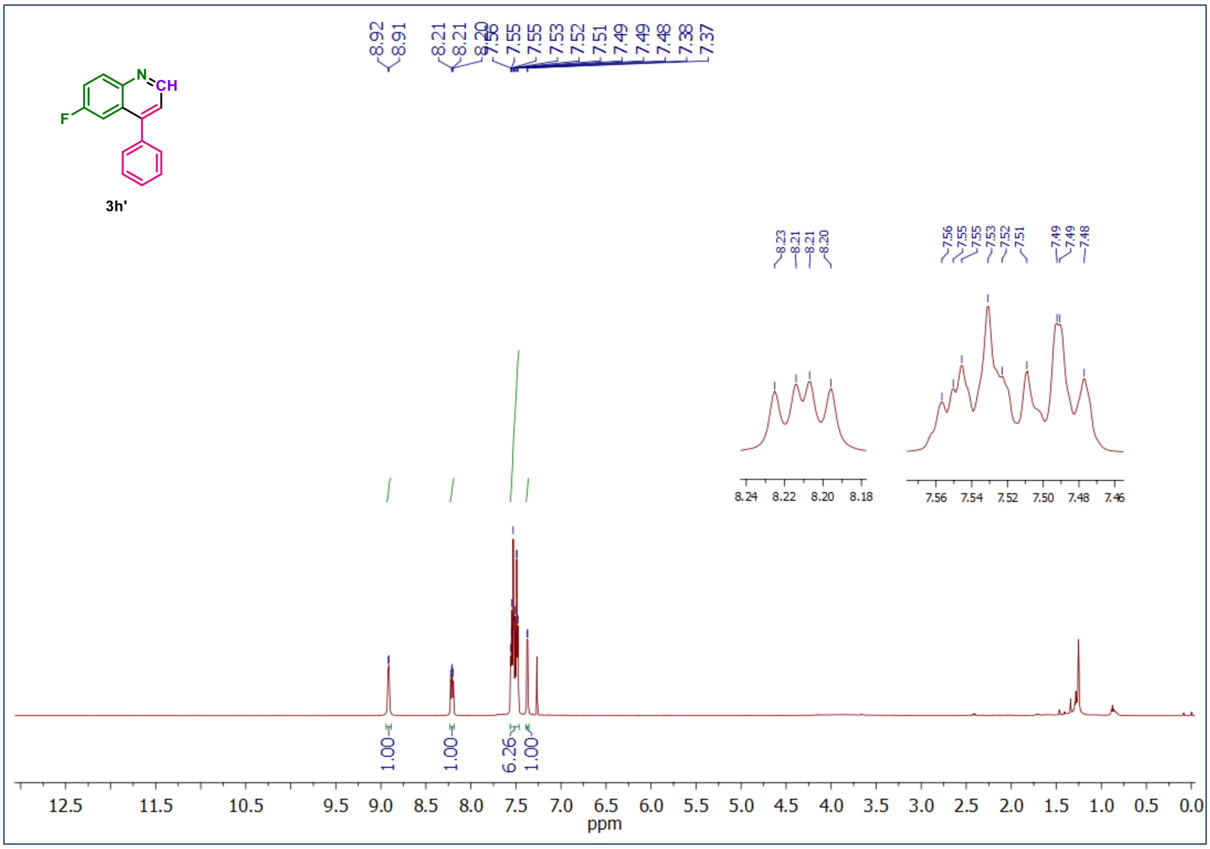

^{13}C{^{1}H} NMR (126 MHz, CDCl3) of 6-fluoro-4-phenylquinoline (3h’)

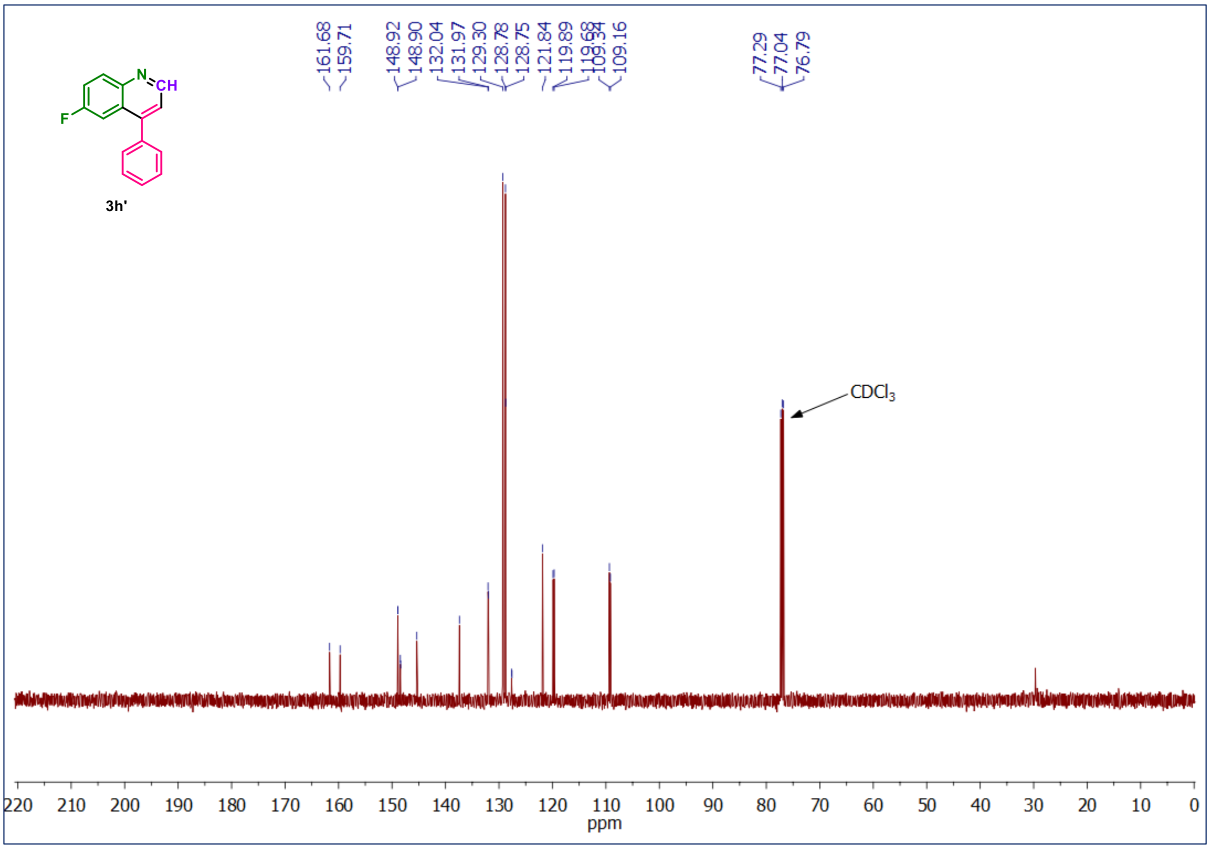

^{1}H NMR (500 MHz, $CDCl_3$) of 8-methyl-2,4-diphenylquinoline (3i)

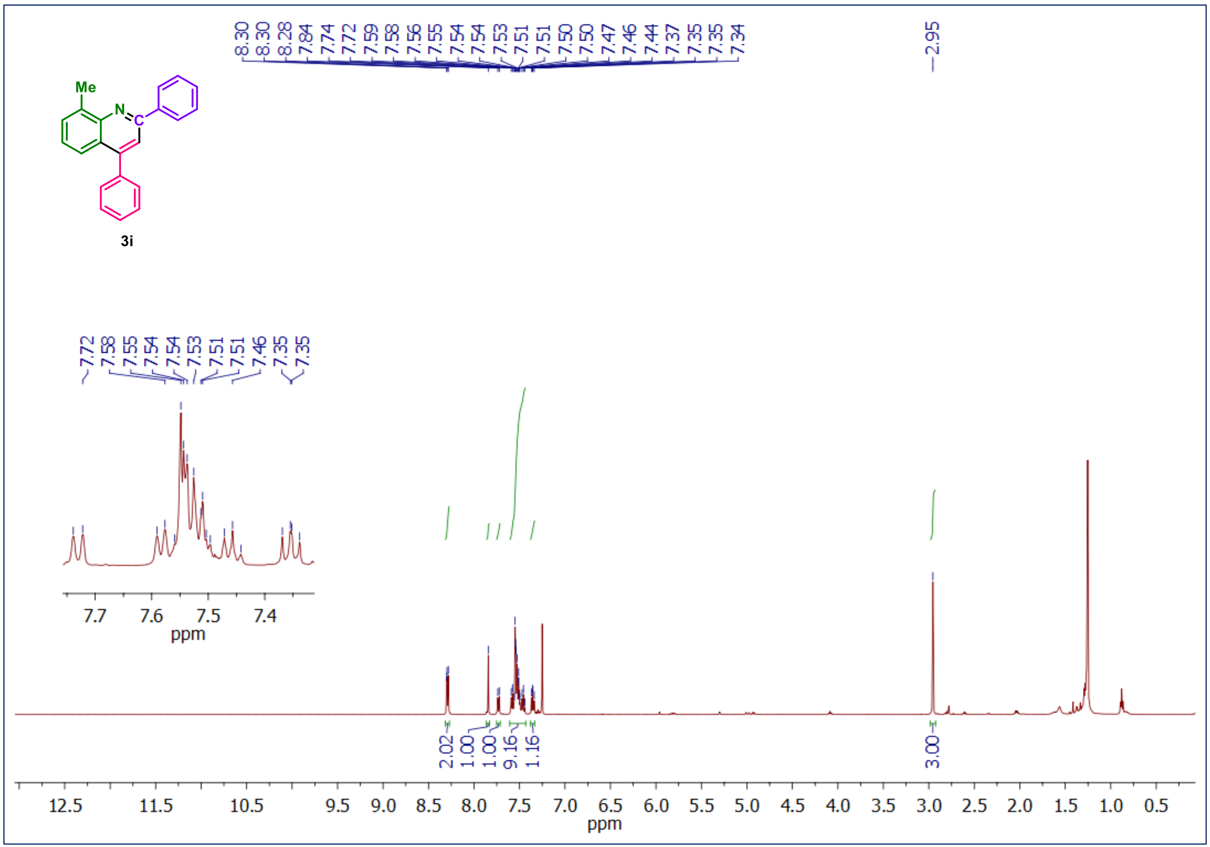

^{13}C{^{1}H} NMR (126 MHz, $CDCl_3$) of 8-methyl-2,4-diphenylquinoline (3i)

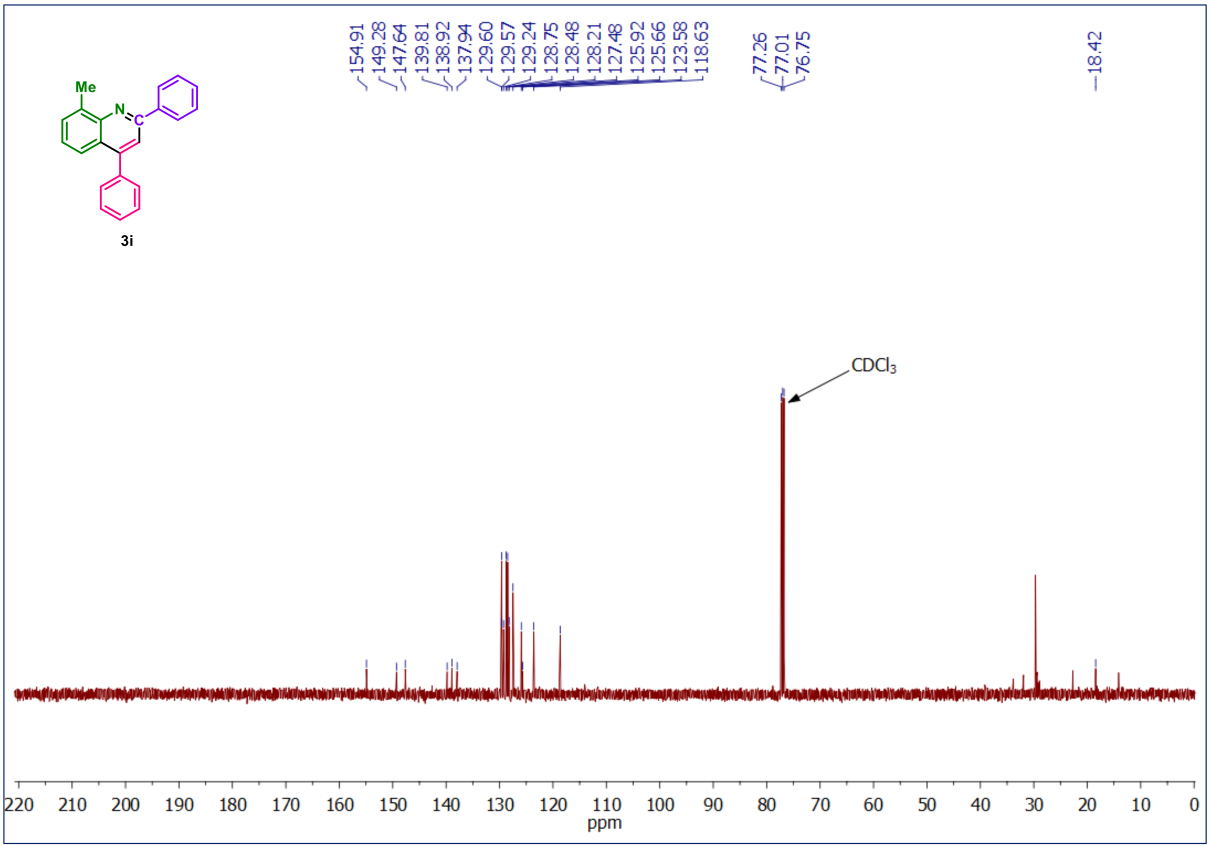

^{1}H NMR (500 MHz, $CDCl_3$) of 8-methyl-4-phenylquinoline (3i')

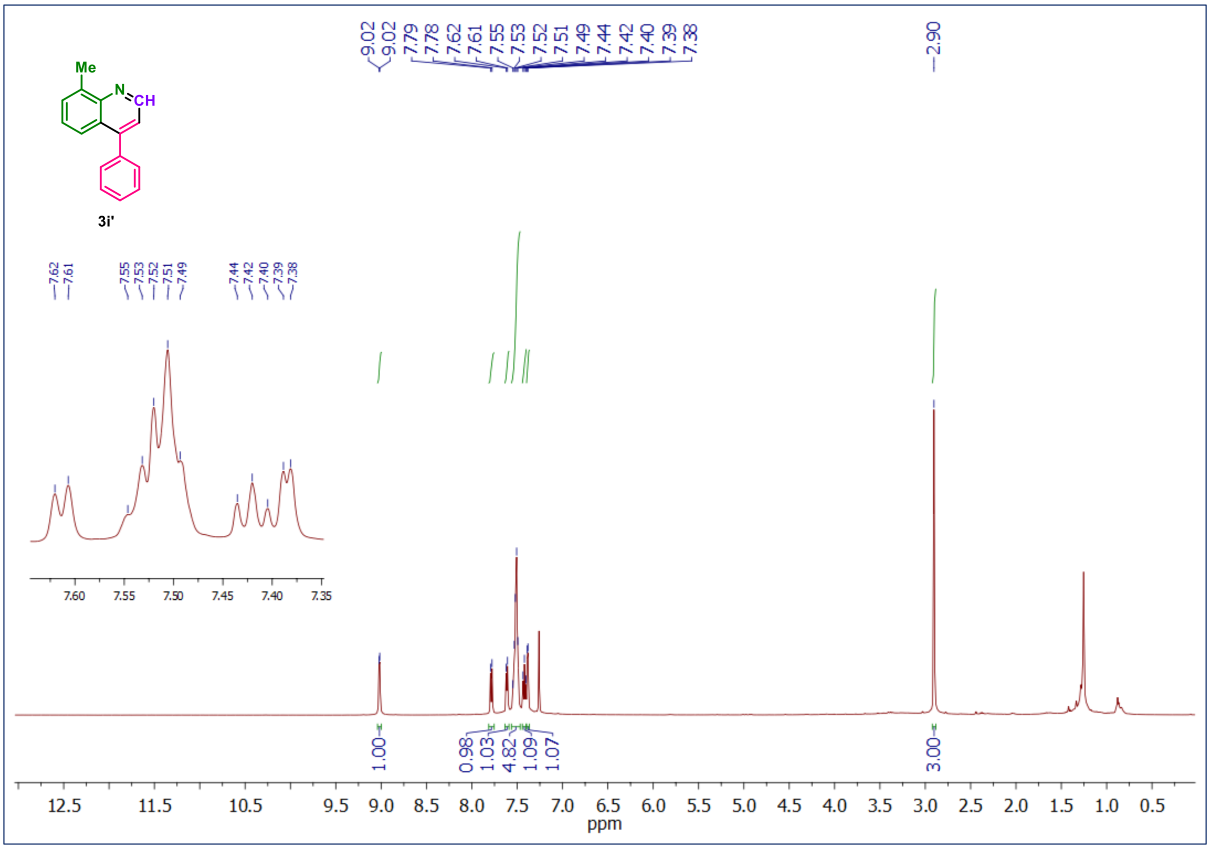

^{13}C{^{1}H} NMR (126 MHz, $CDCl_3$) of 8-methyl-4-phenylquinoline (3i')

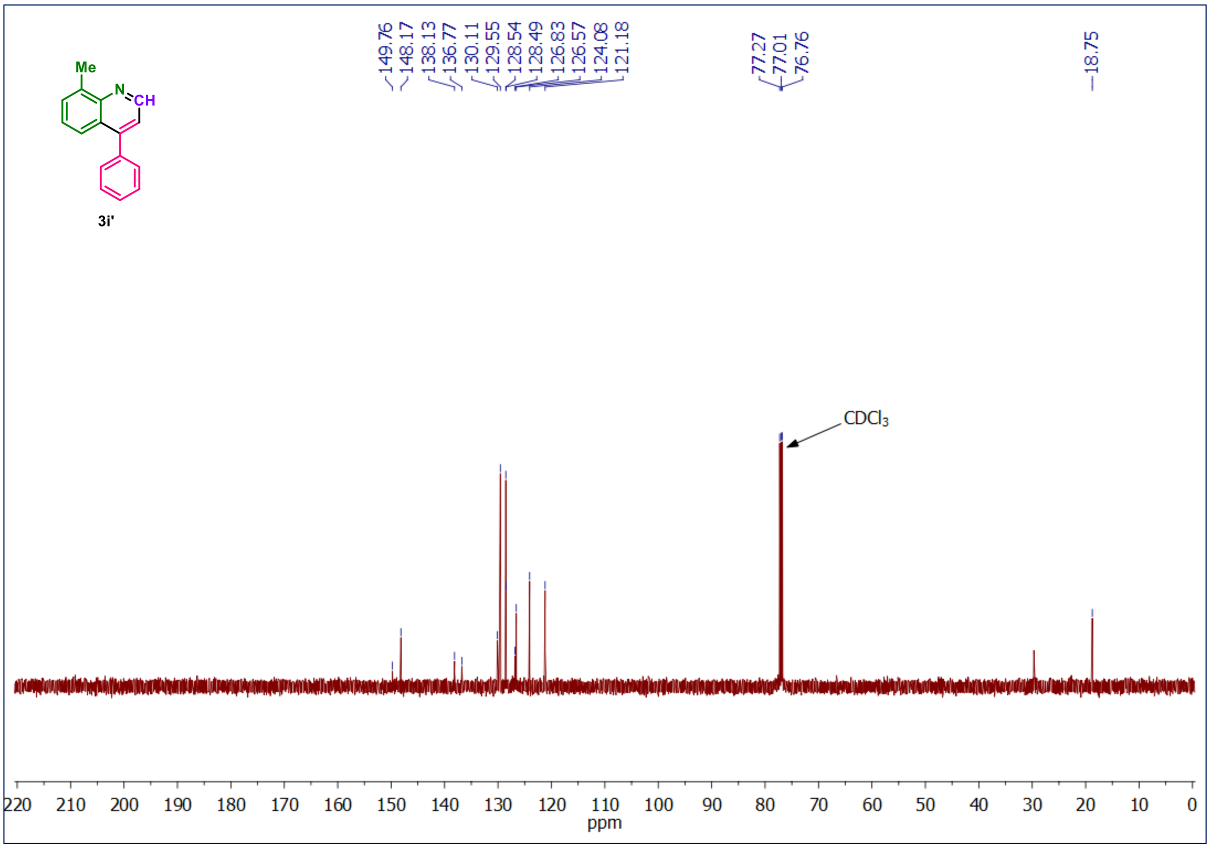

^{1}H NMR (500 MHz, $CDCl_3$) of 6-methyl-2,4-di-*p*-tolylquinoline (3m)

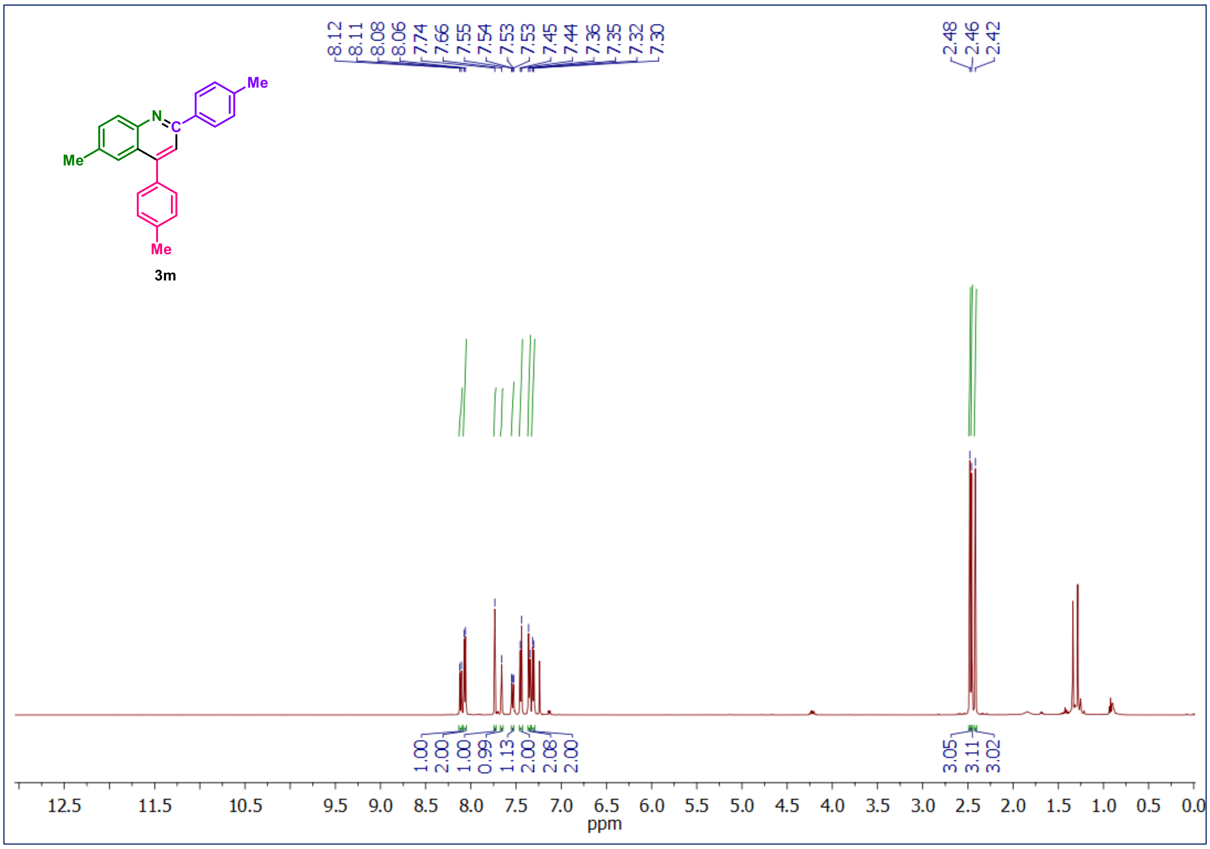

$^{13}C\{^{1}H\}$ NMR (126 MHz, $CDCl_3$) of 6-methyl-2,4-di-*p*-tolylquinoline (3m)

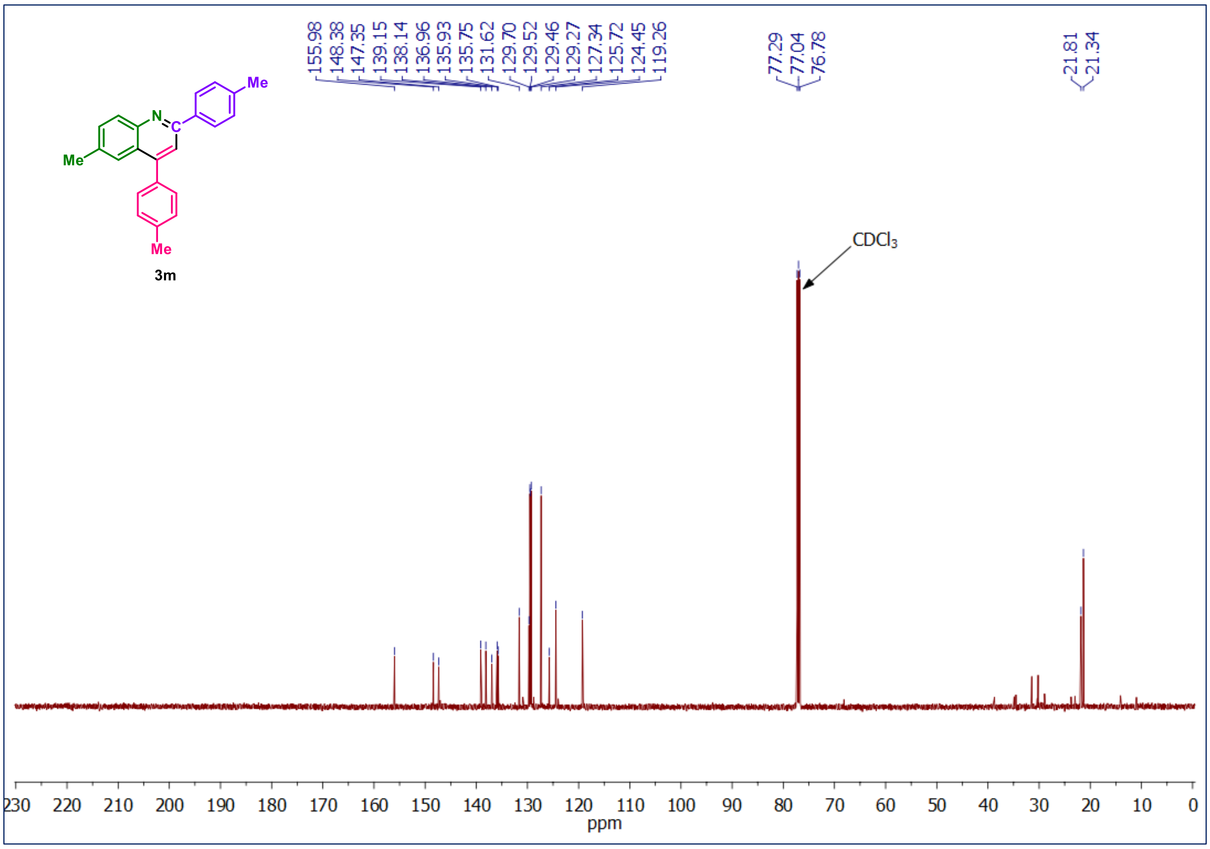

^{1}H NMR (500 MHz, $CDCl_3$) of 6-methyl-4-(*p*-tolyl)quinoline (3m')

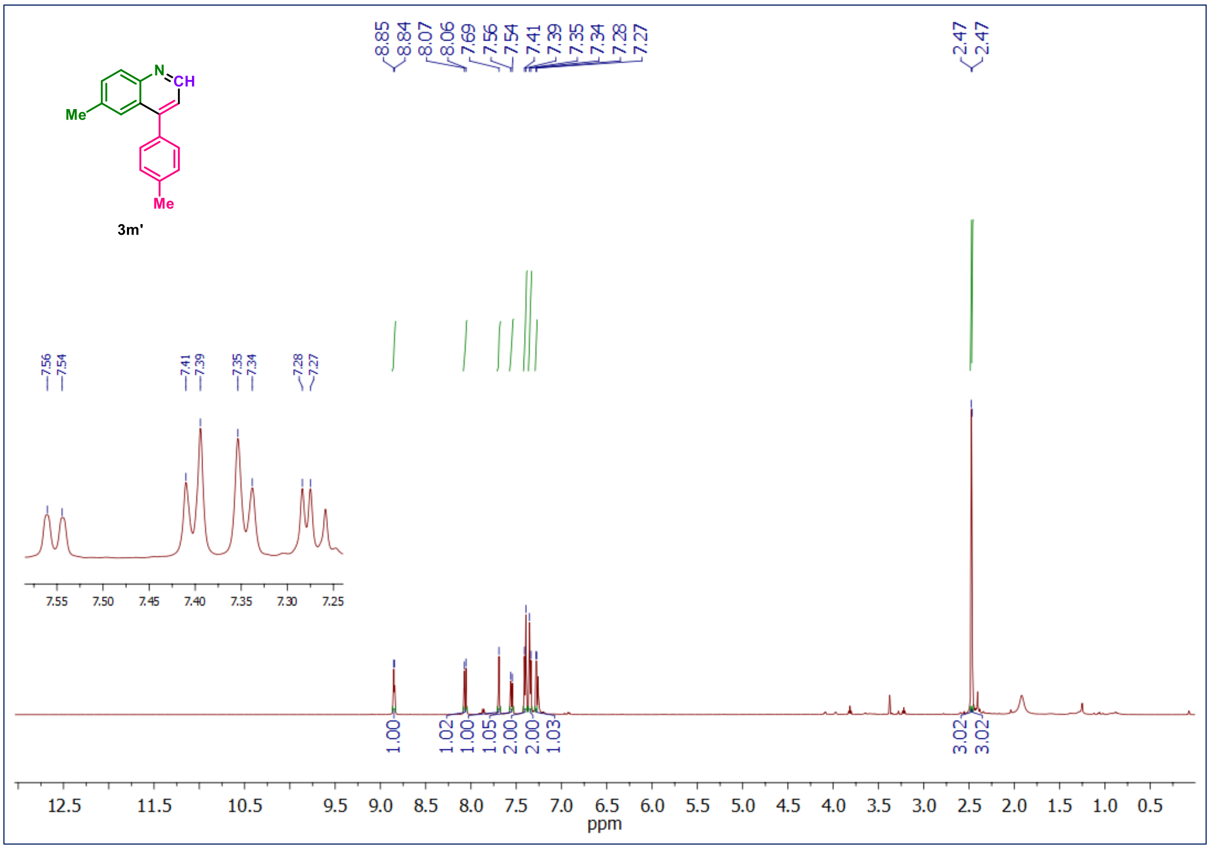

^{13}C{^{1}H} NMR (126 MHz, $CDCl_3$) of 6-methyl-4-(*p*-tolyl)quinoline (3m')

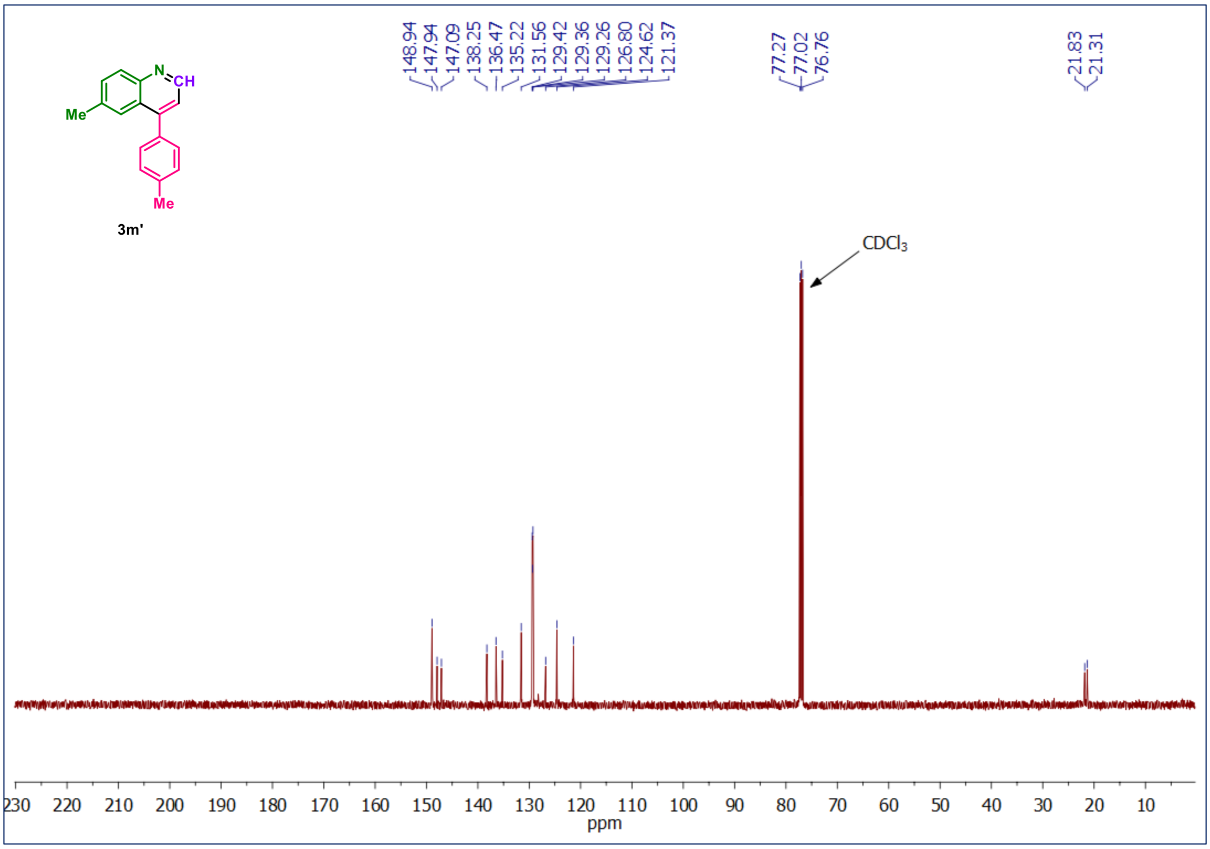

^{1}H NMR (500 MHz, $CDCl_3$) of 2,4-bis(4-(*tert*-butyl)phenyl)-6-methylquinoline (3n)

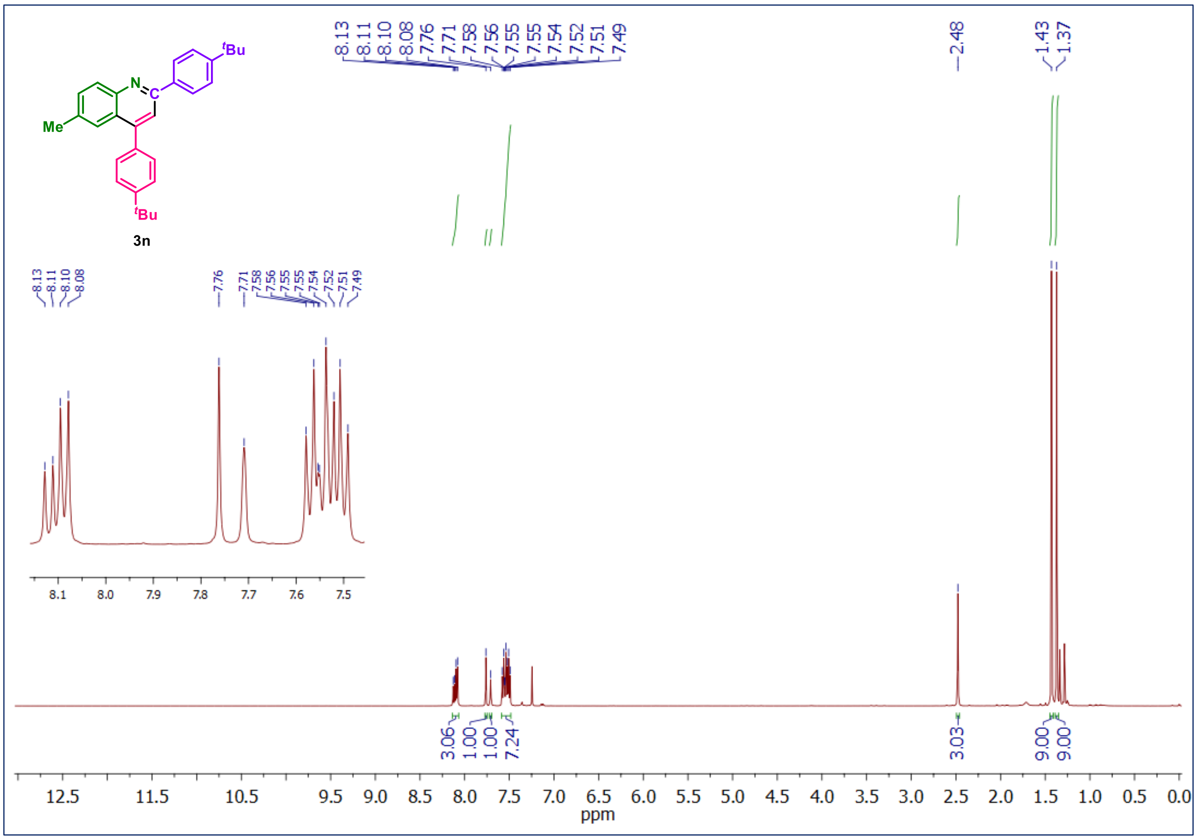

^{13}C{^{1}H} NMR (126 MHz, $CDCl_3$) of 2,4-bis(4-(*tert*-butyl)phenyl)-6-methylquinoline (3n)

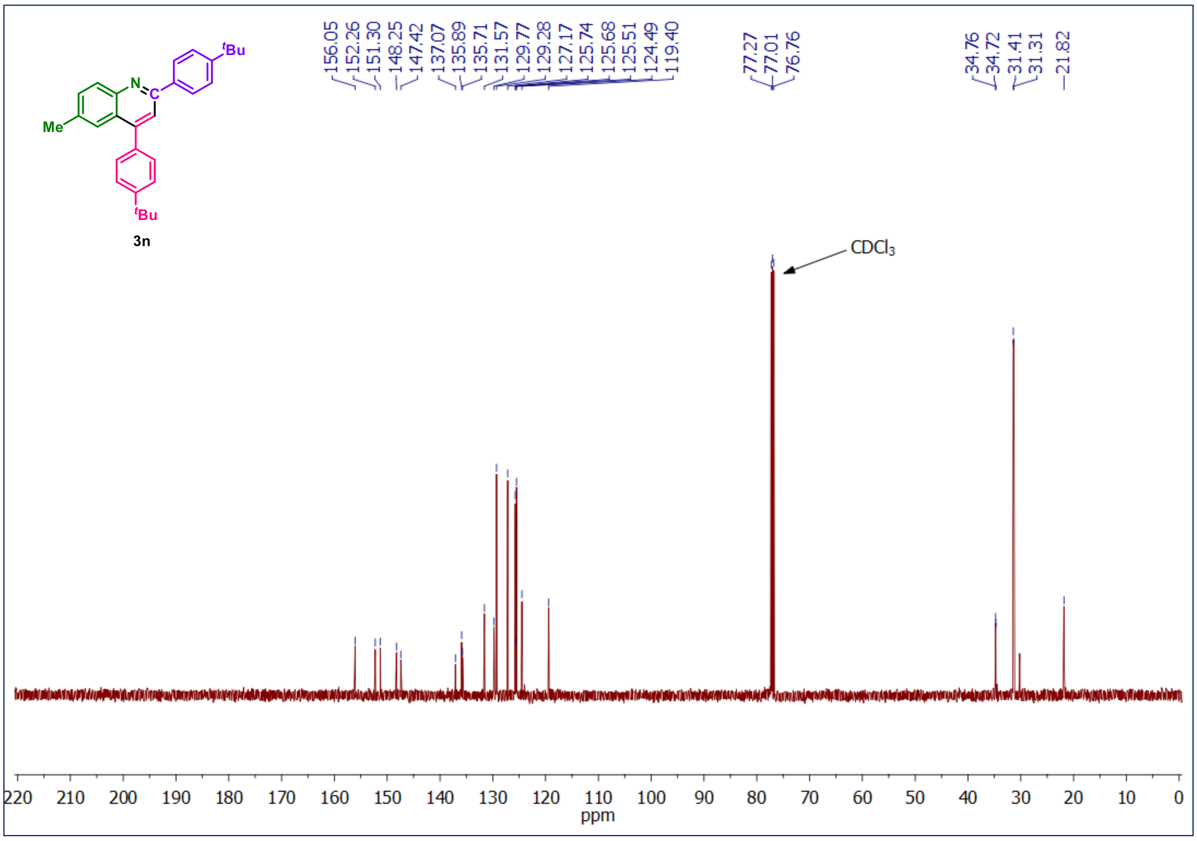

^{1}H NMR (500 MHz, $CDCl_3$) of 4-(4-(*tert*-butyl)phenyl)-6-methylquinoline (3n')

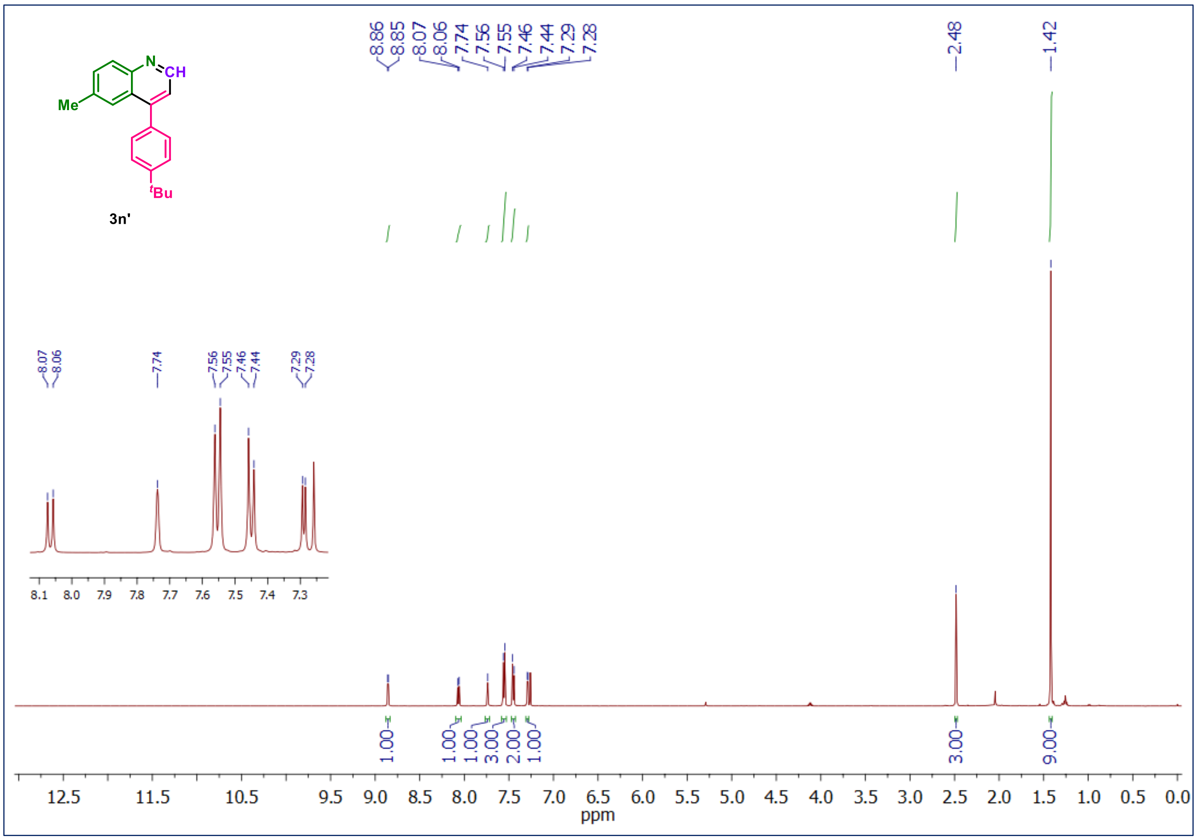

$^{13}C\{^{1}H\}$ NMR (126 MHz, $CDCl_3$) of 4-(4-(*tert*-butyl)phenyl)-6-methylquinoline (3n')

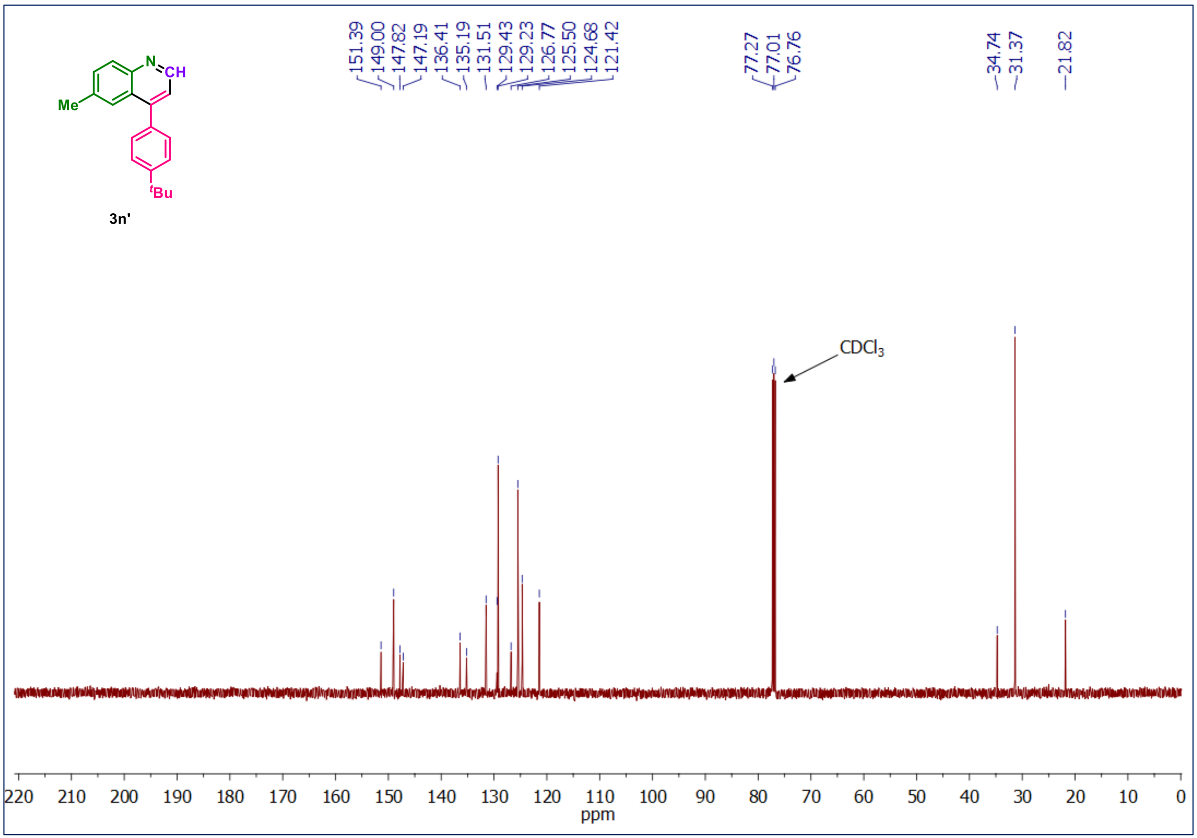

^{1}H NMR (500 MHz, CDCl3) of 2,4-bis(4-bromophenyl)-6-methylquinoline (3o)

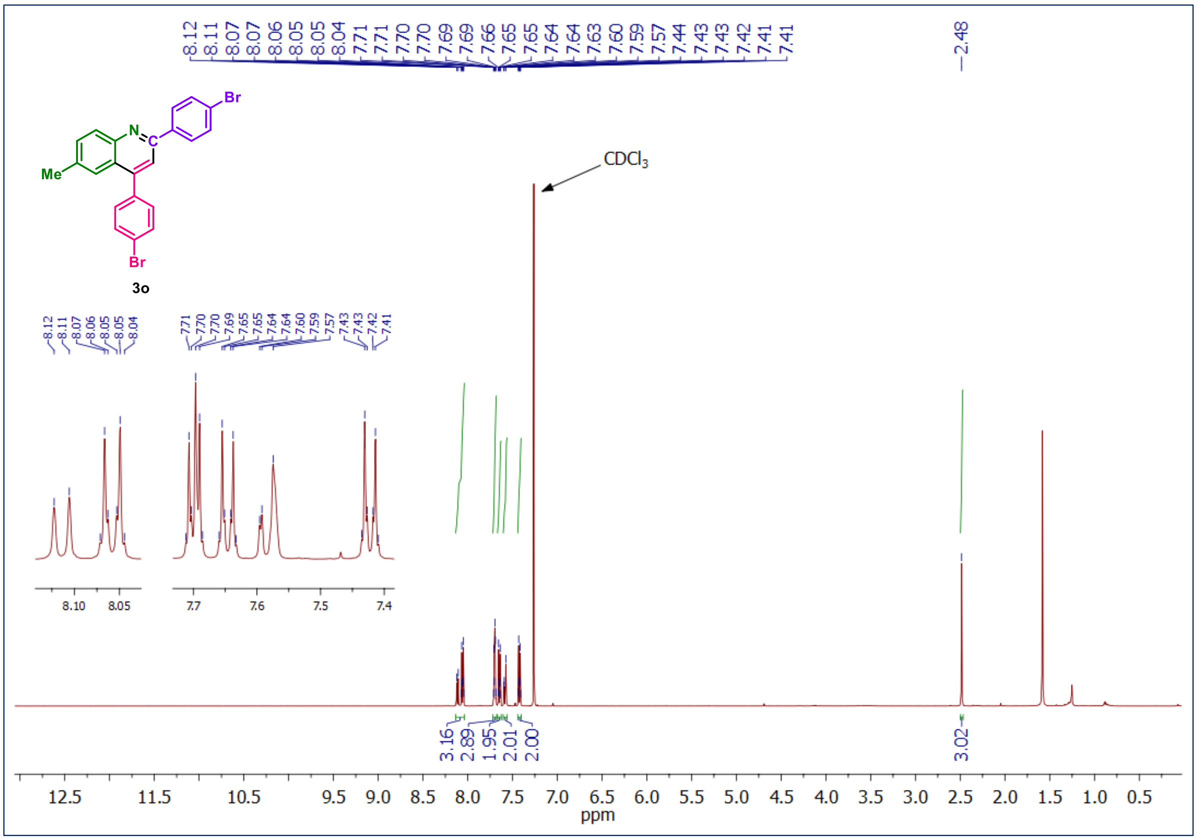

^{13}C{^{1}H} NMR (126 MHz, CDCl3) of 2,4-bis(4-bromophenyl)-6-methylquinoline (3o)

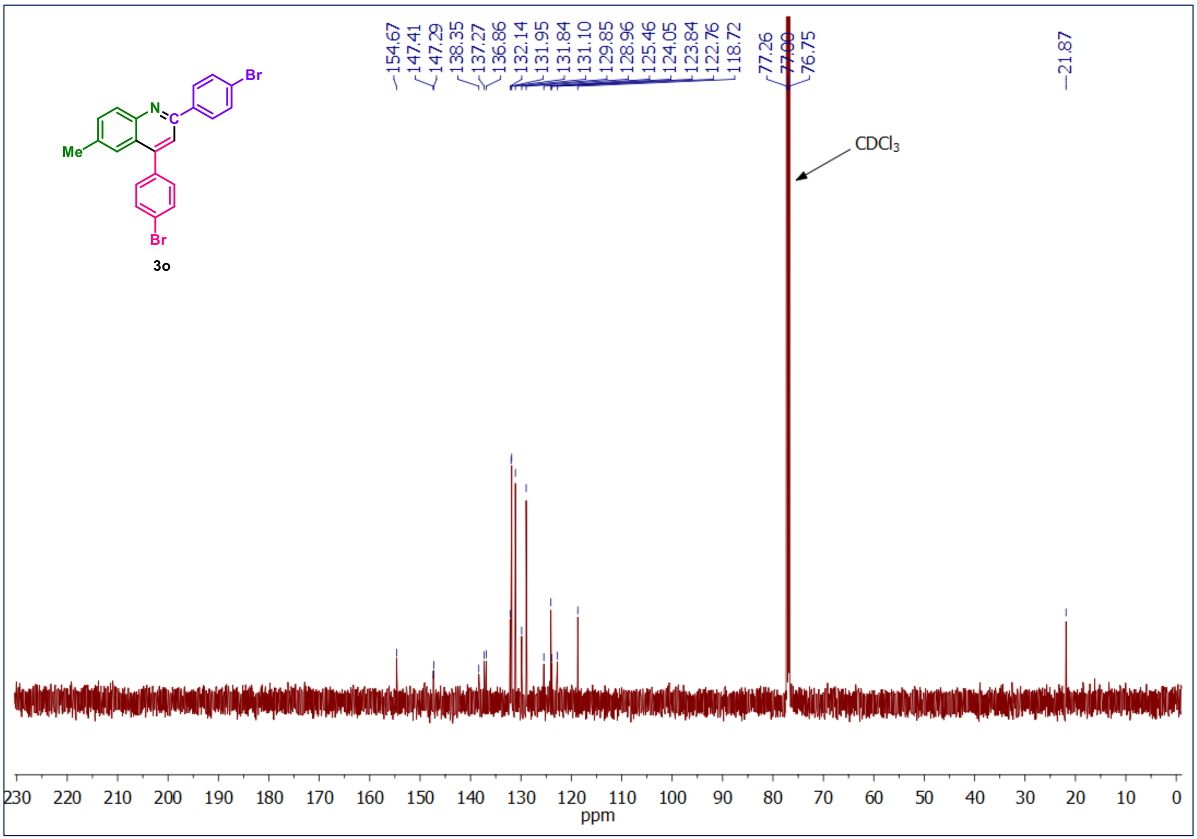

^{1}H NMR (500 MHz, $CDCl_3$) of 4-(4-bromophenyl)-6-methylquinoline (3o')

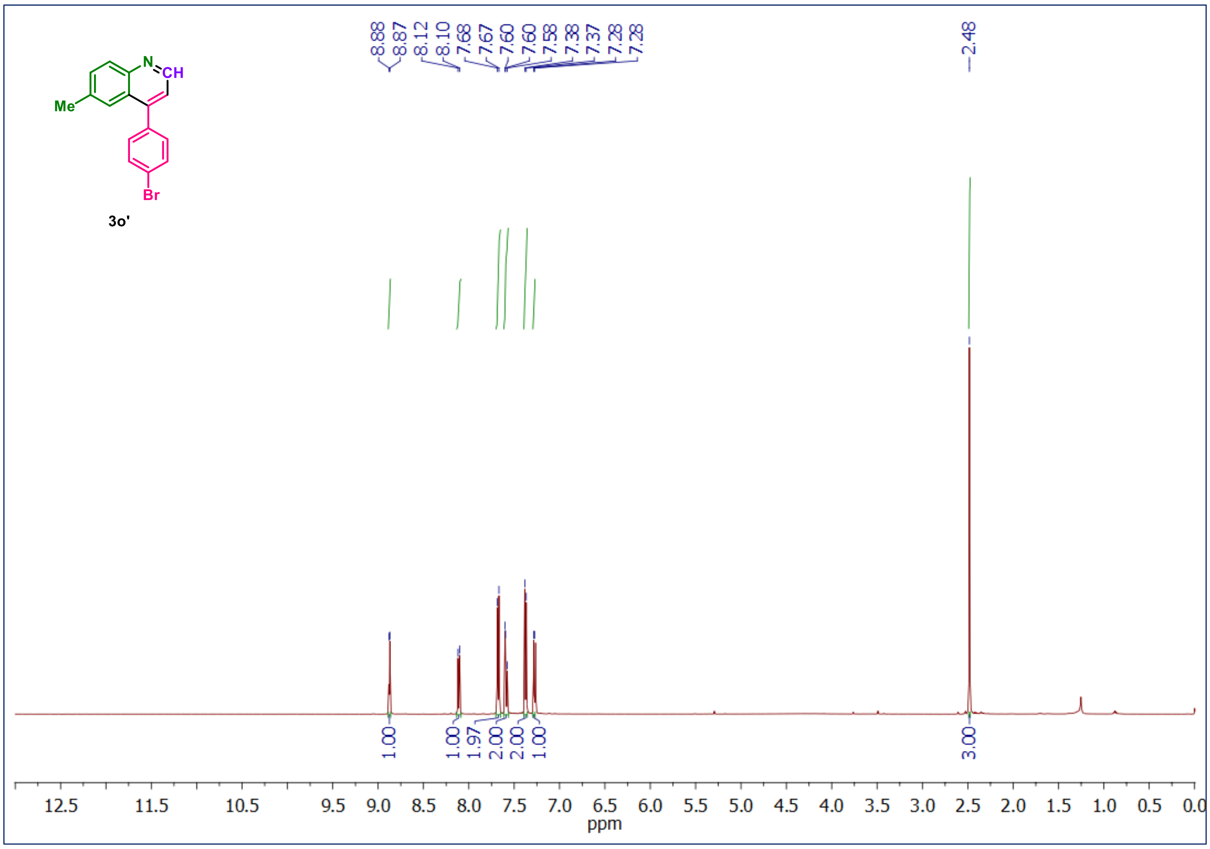

^{13}C{^{1}H} NMR (126 MHz, $CDCl_3$) of 4-(4-bromophenyl)-6-methylquinoline (3o')

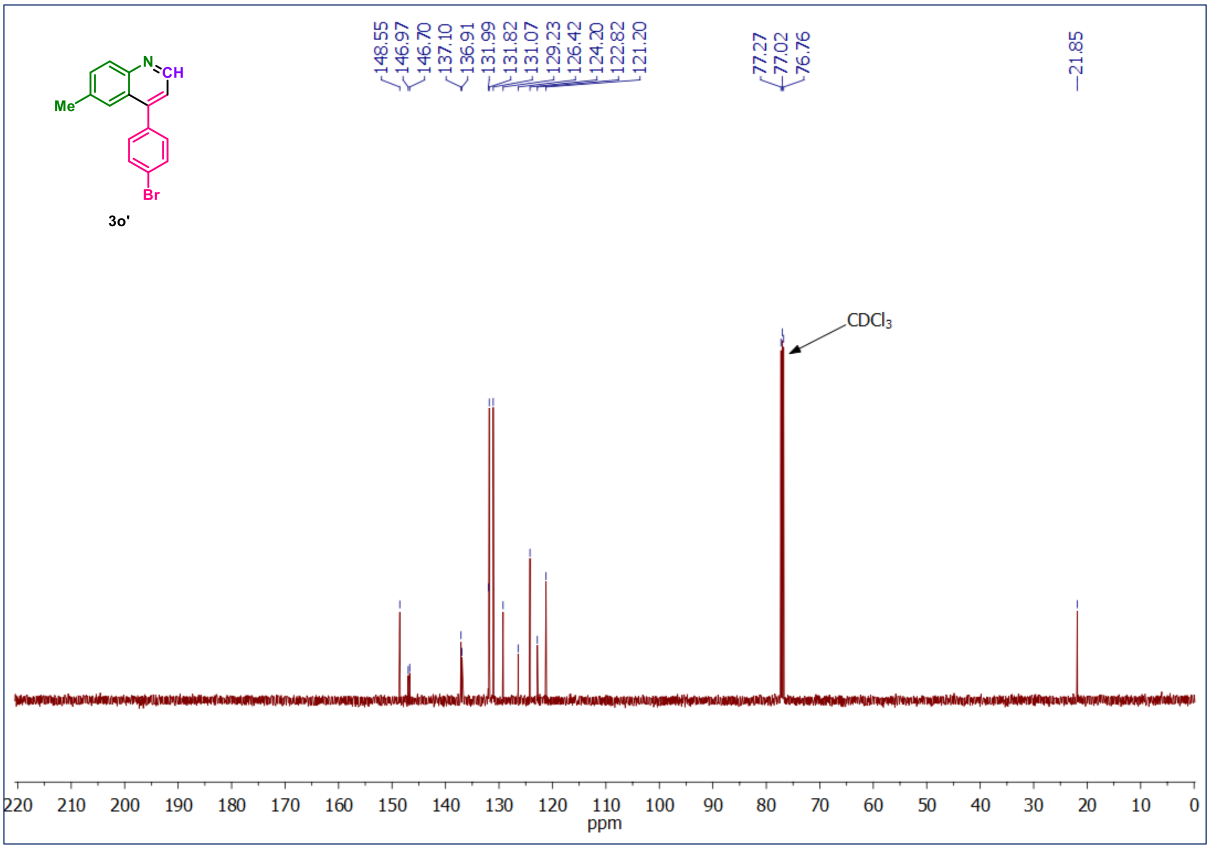

^{1}H NMR (500 MHz, $CDCl_3$) of 2,4-bis(4-chlorophenyl)-6-methylquinoline (3p)

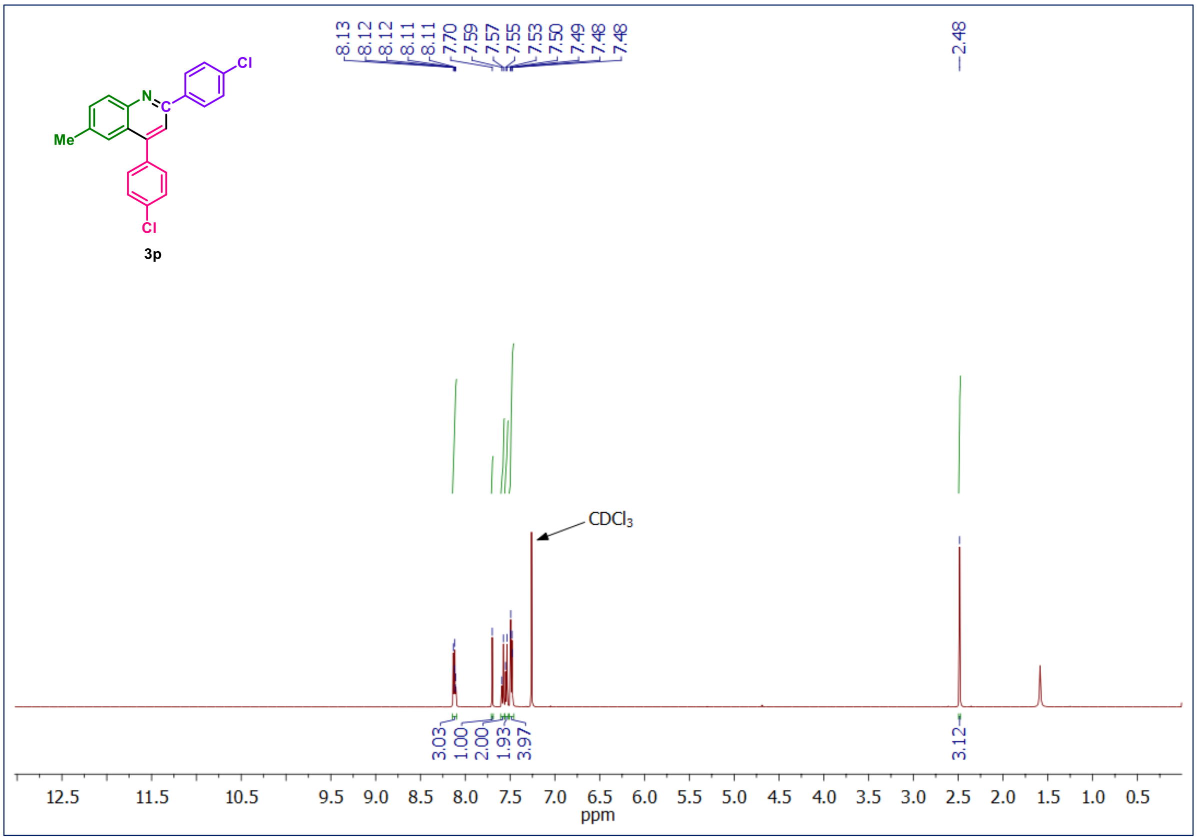

$^{13}C\{^1H\}$ NMR (126 MHz, $CDCl_3$) of 2,4-bis(4-chlorophenyl)-6-methylquinoline (3p)

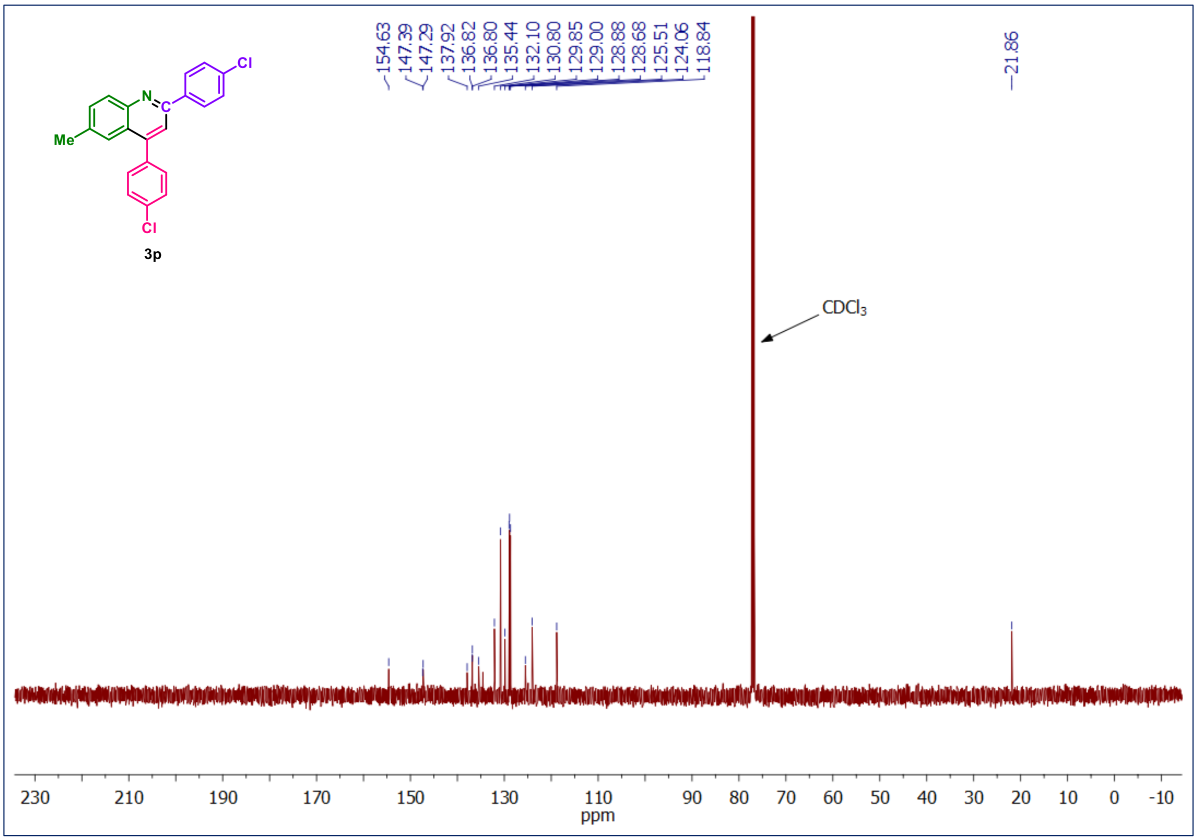

^{1}H NMR (500 MHz, CDCl3) of 4-(4-chlorophenyl)-6-methylquinoline (3p')

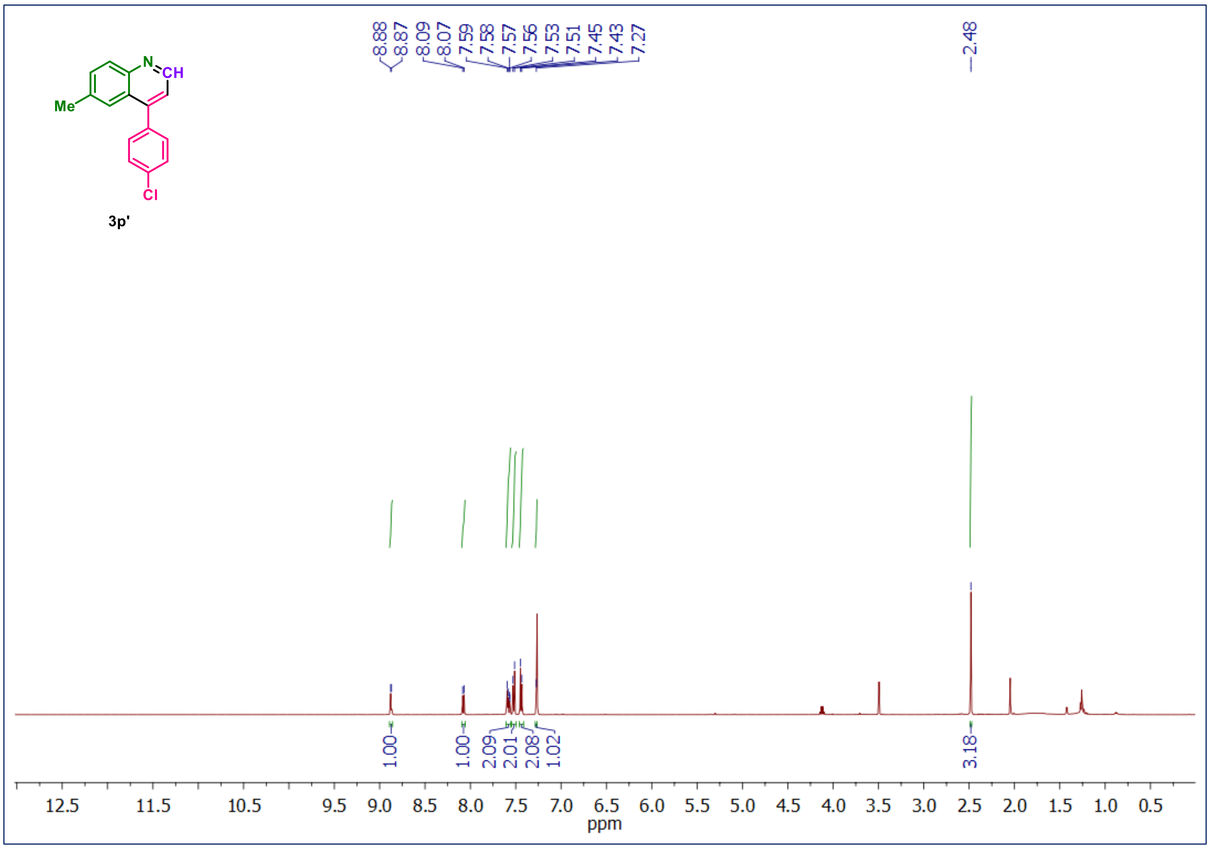

^{13}C{^{1}H} NMR (126 MHz, CDCl3) of 4-(4-chlorophenyl)-6-methylquinoline (3p')

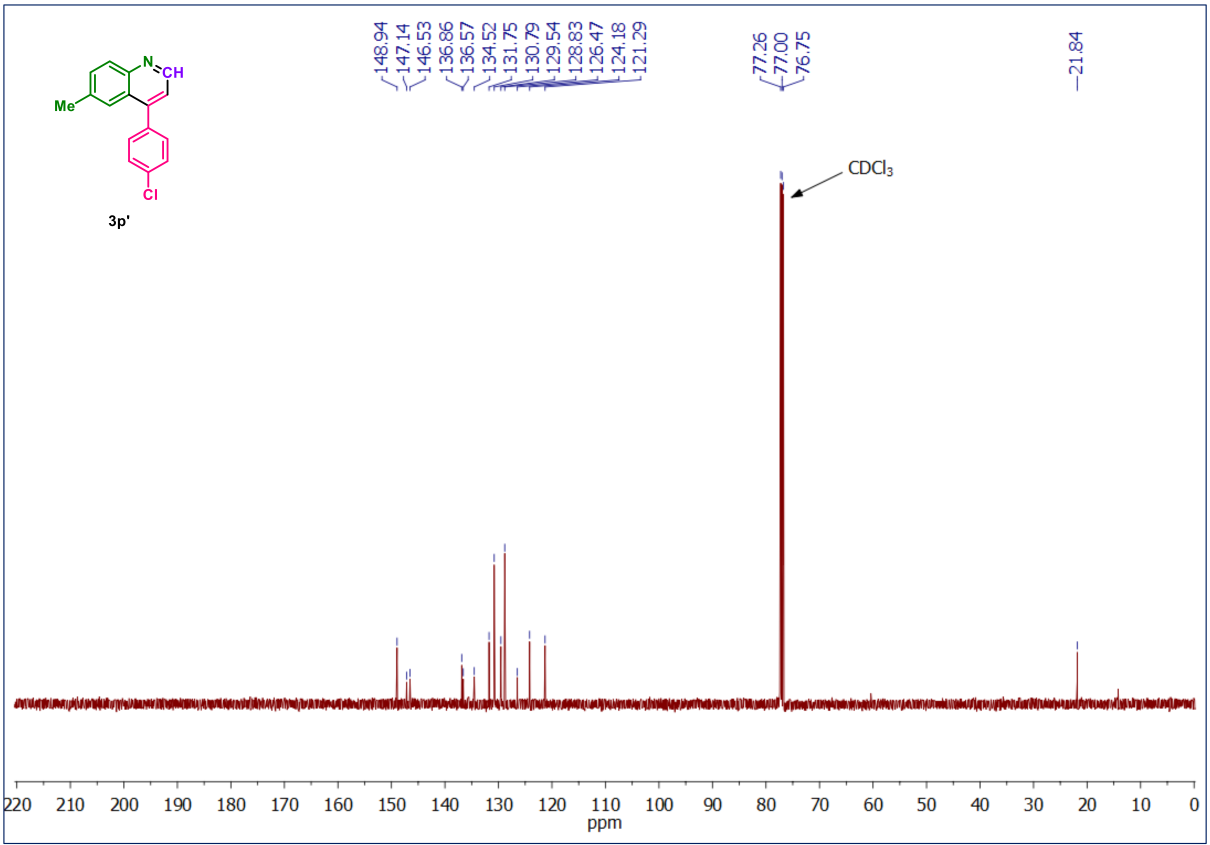

^{1}H NMR (500 MHz, $CDCl_3$) of 2,4-bis(4-fluorophenyl)-6-methylquinoline (3q)

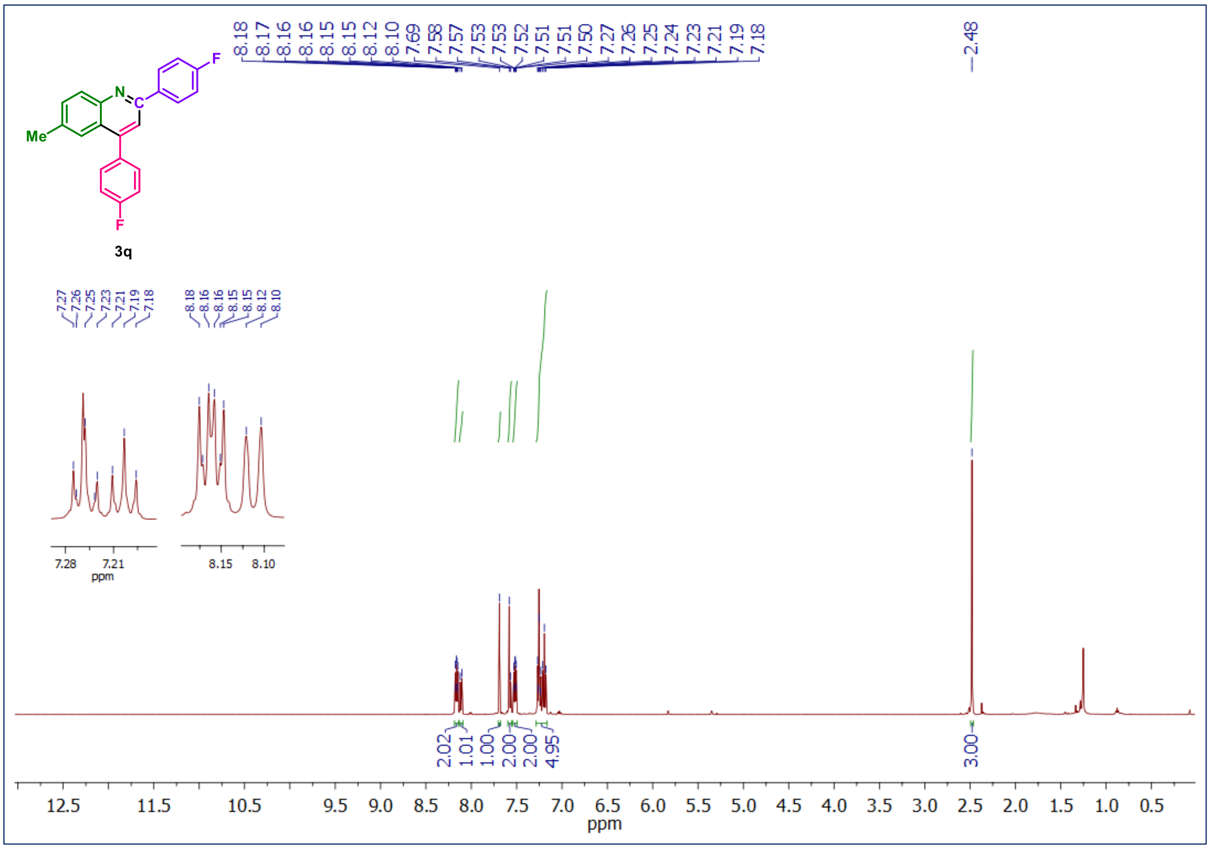

$^{13}C\{^{1}H\}$ NMR (126 MHz, $CDCl_3$) of 2,4-bis(4-fluorophenyl)-6-methylquinoline (3q)

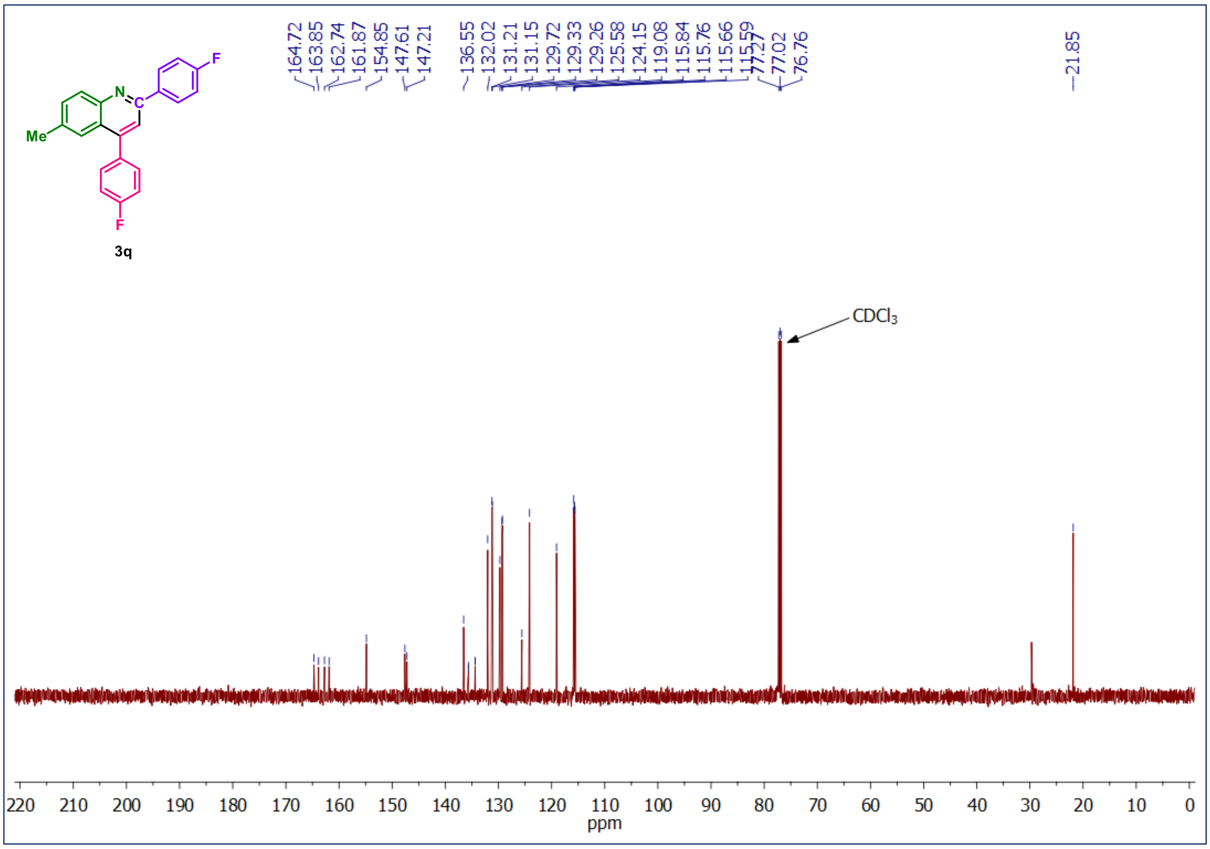

^{1}H NMR (500 MHz, $CDCl_3$) of 2,4-bis(3-methoxyphenyl)-6-methylquinoline (3r)

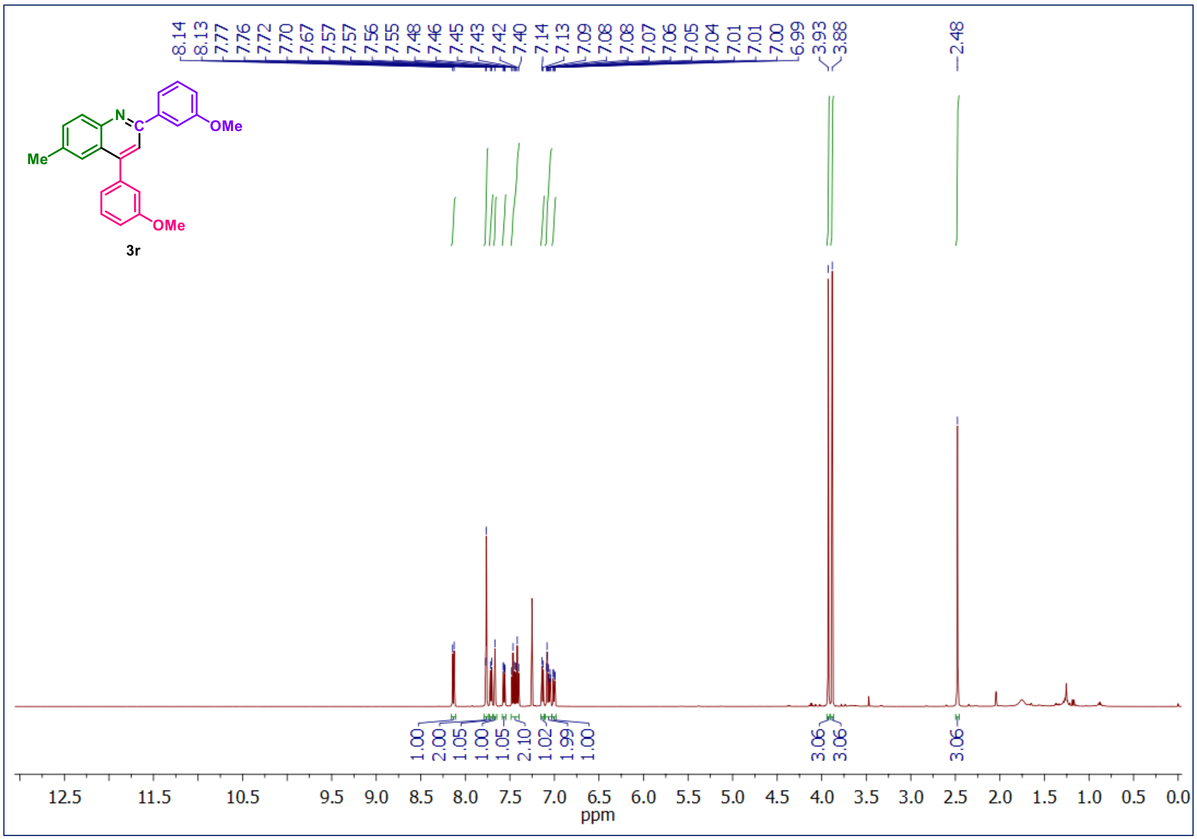

^{13}C{^{1}H} NMR (126 MHz, $CDCl_3$) of 2,4-bis(3-methoxyphenyl)-6-methylquinoline (3r)

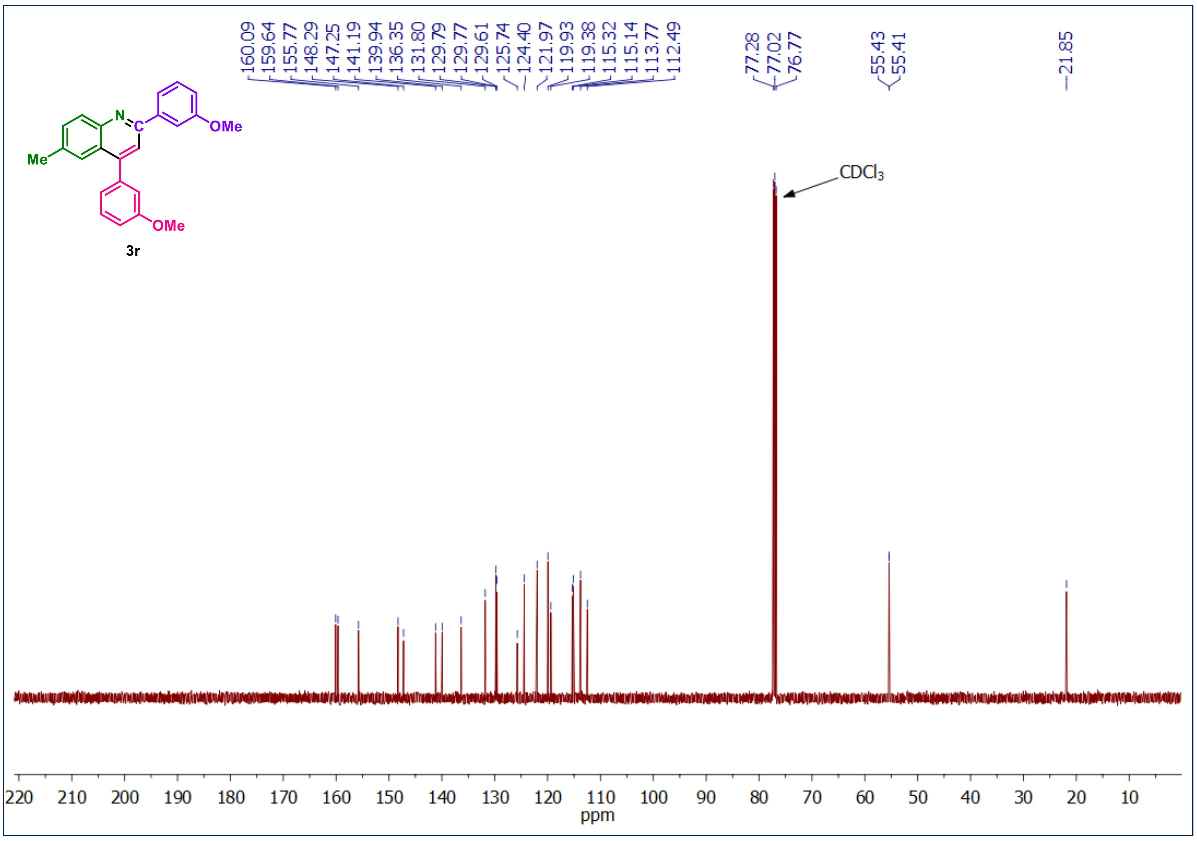

^{1}H NMR (500 MHz, CDCl$_3$) of 4-(3-methoxyphenyl)-6-methylquinoline (3r’)

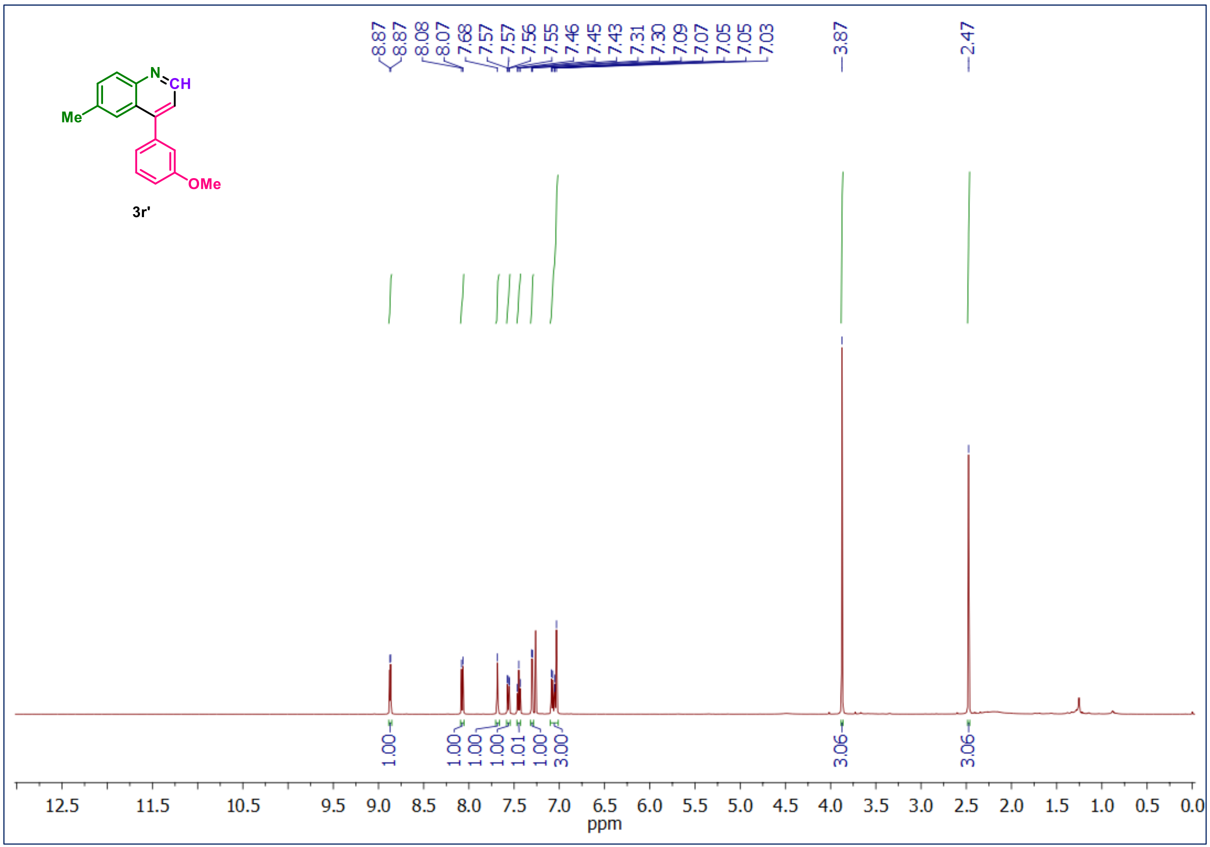

^{13}C{^{1}H} NMR (126 MHz, CDCl$_3$) of 4-(3-methoxyphenyl)-6-methylquinoline (3r’)

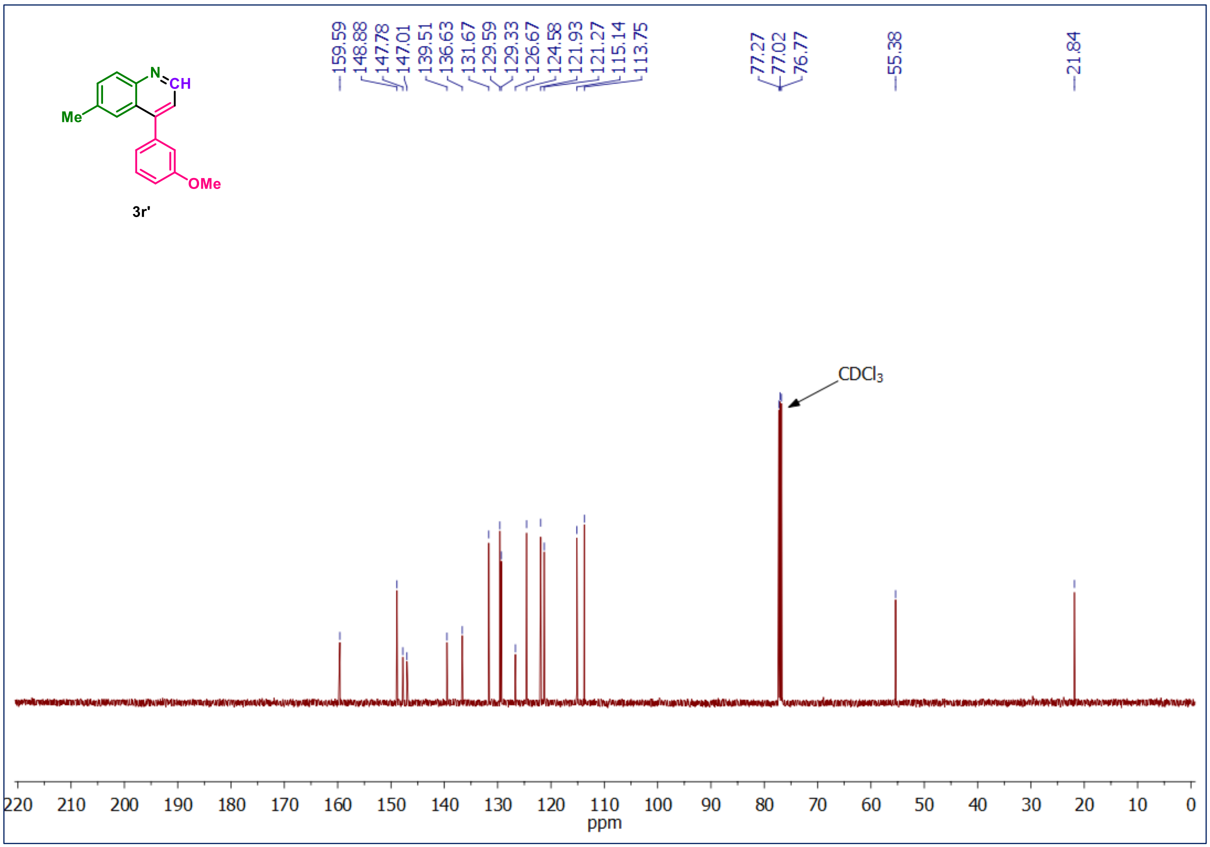

GC–MS spectrum of 8-fluoro-2,4-diphenylquinoline (3j)

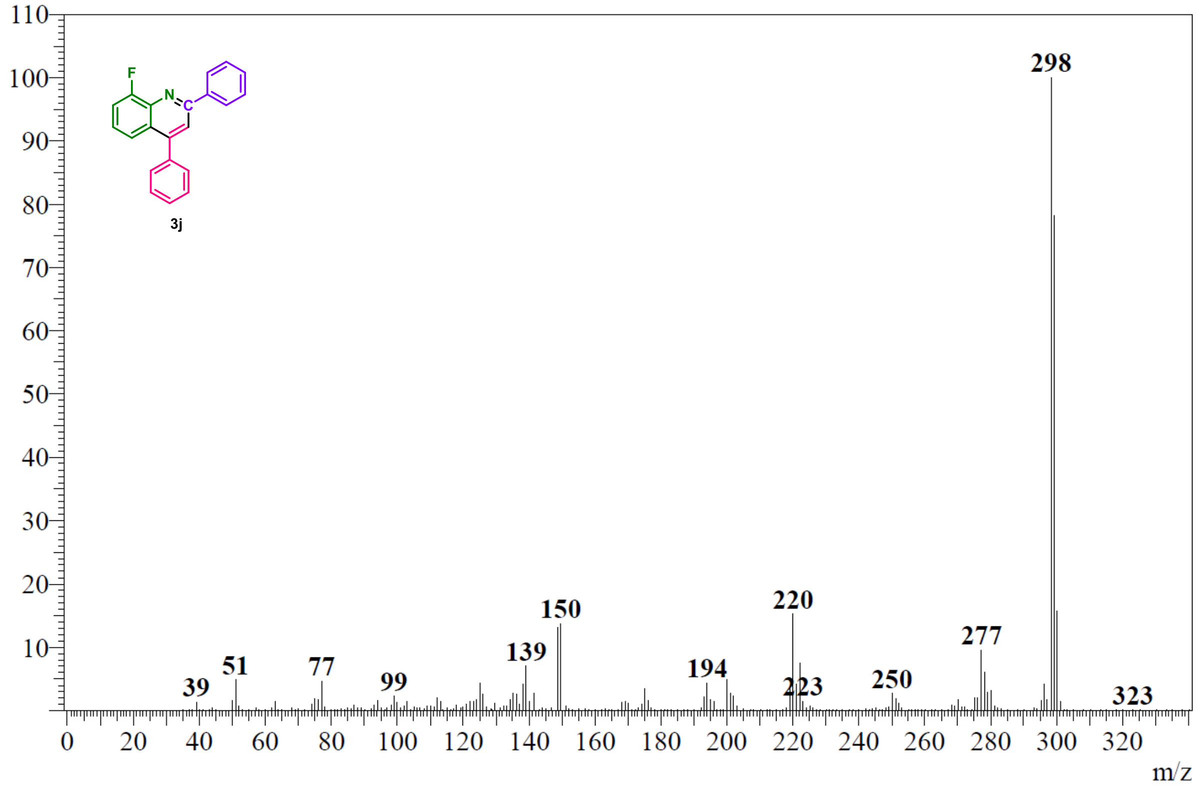

GC–MS spectrum of 8-fluoro-4-phenylquinoline (3j’)

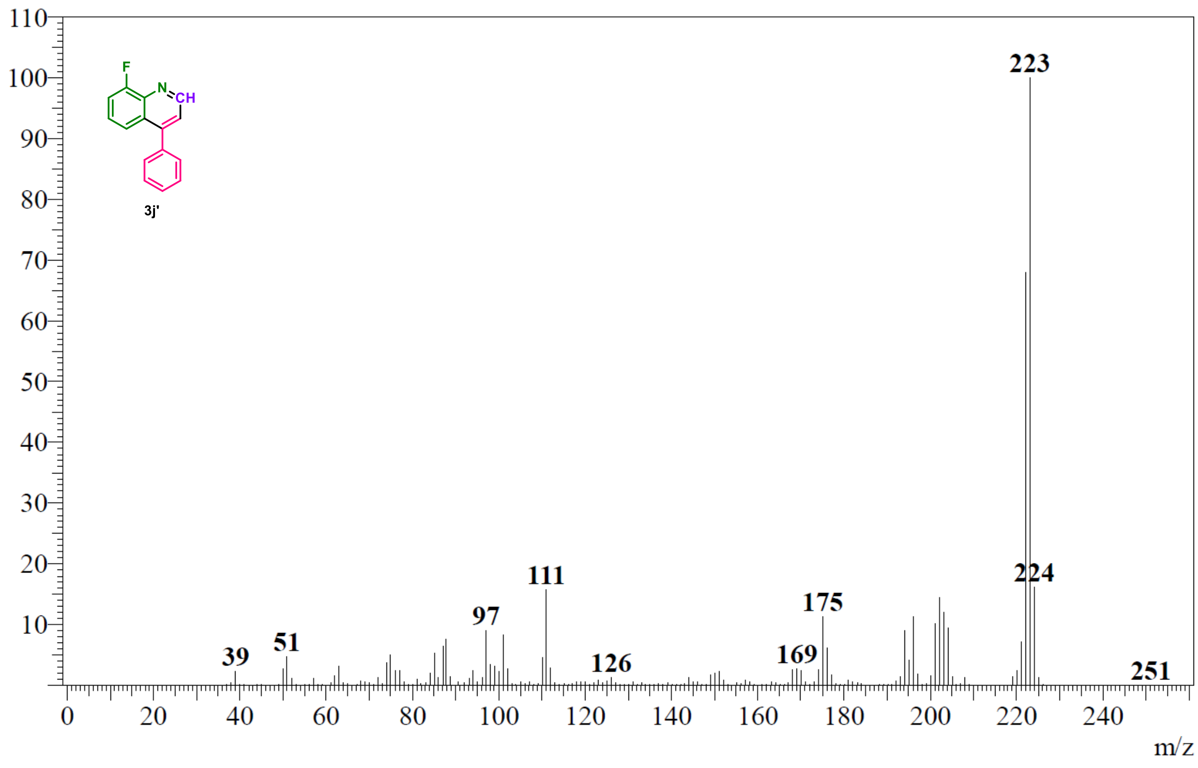

GC–MS spectrum of 7-methyl-2,4-diphenylquinoline (3k) + 5-methyl-2,4-diphenylquinoline (3kk)

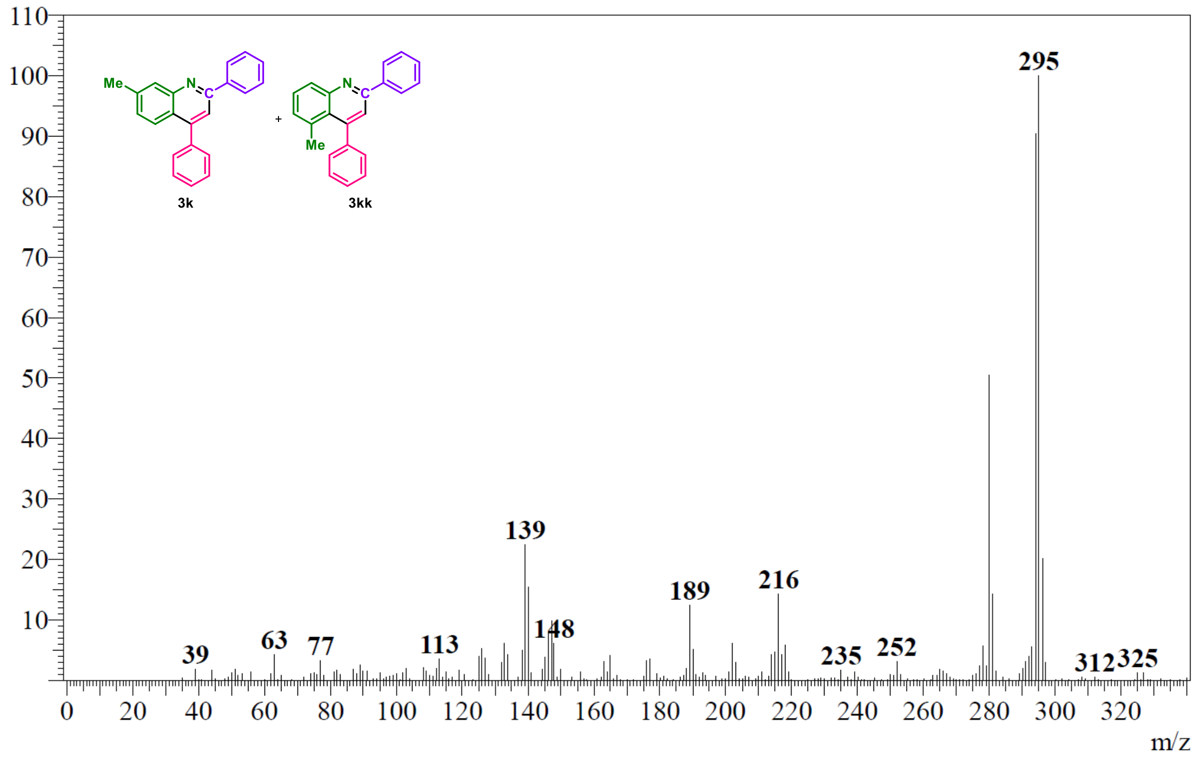

GC–MS spectrum of 7-methyl-4-phenylquinoline (3k’) + 5-methyl-4-phenylquinoline (3kk’)

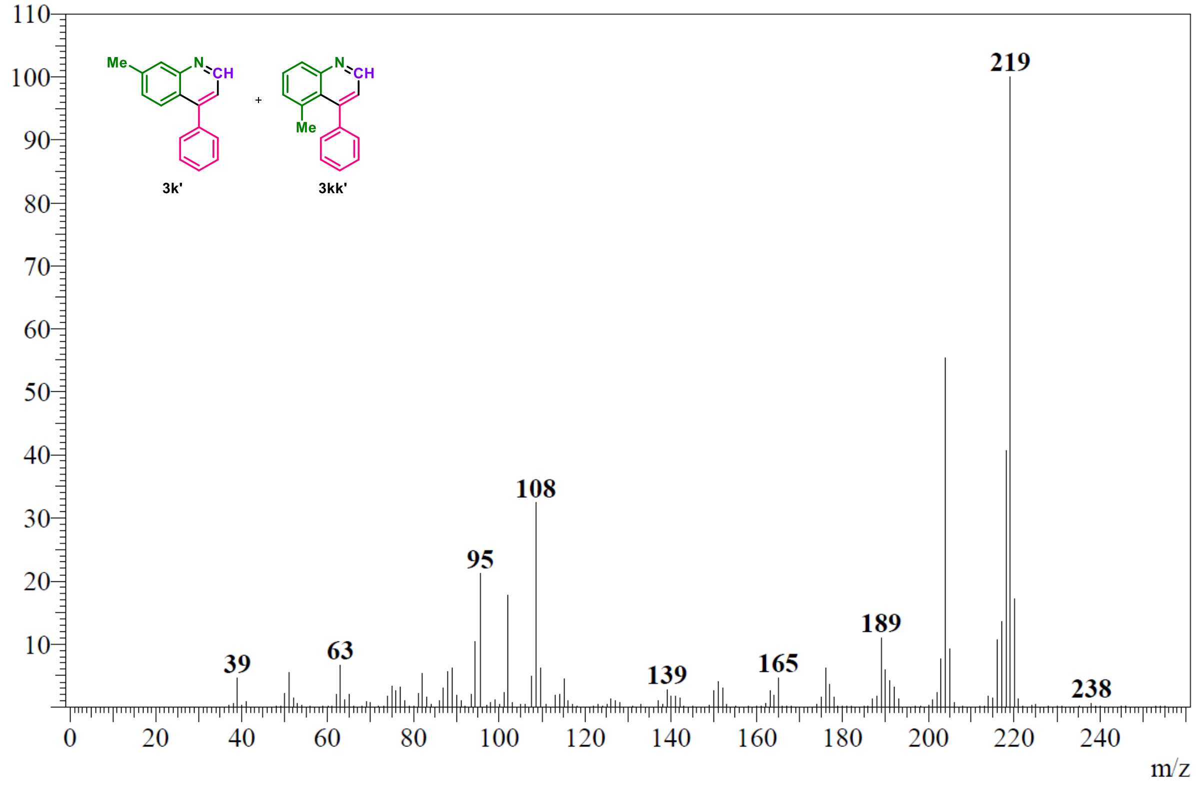

GC–MS spectrum of 6,7-dimethyl-2,4-diphenylquinoline (3l) + 5,6-dimethyl-2,4-diphenylquinoline (3ll)

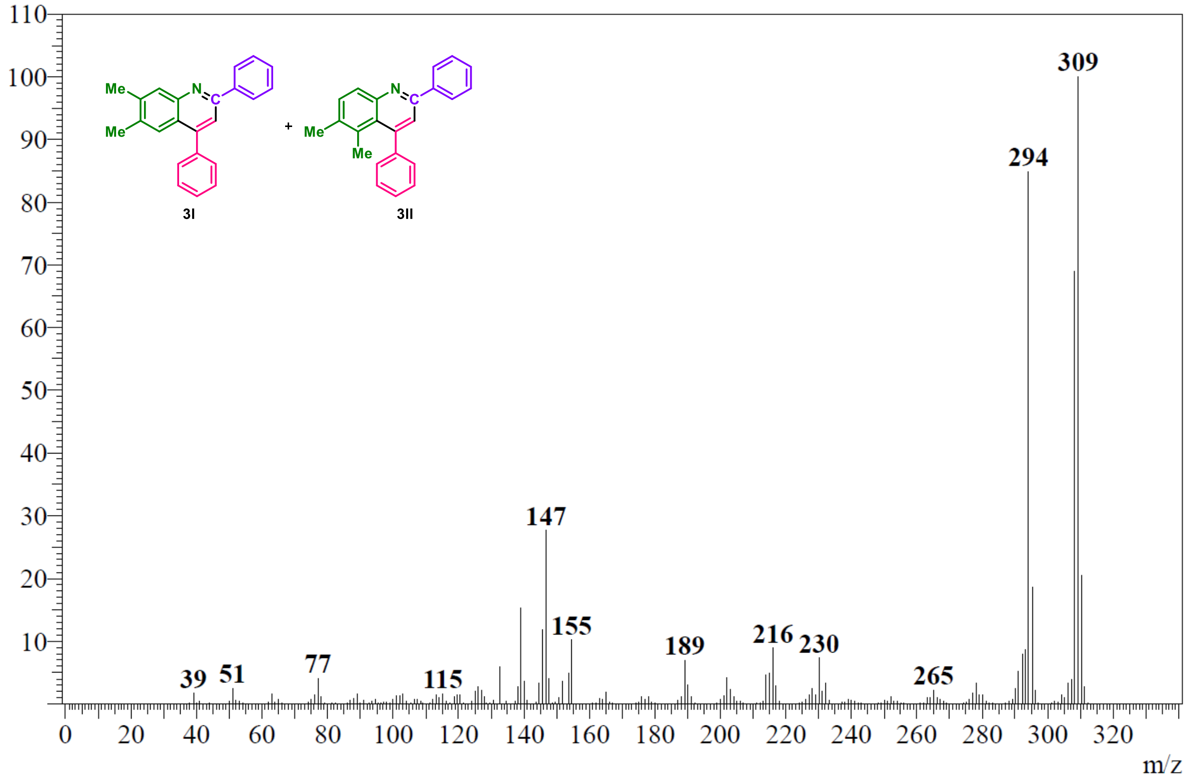

GC–MS spectrum of 6,7-dimethyl-4-phenylquinoline (3l’) + 5,6-dimethyl-4-phenylquinoline (3ll’)

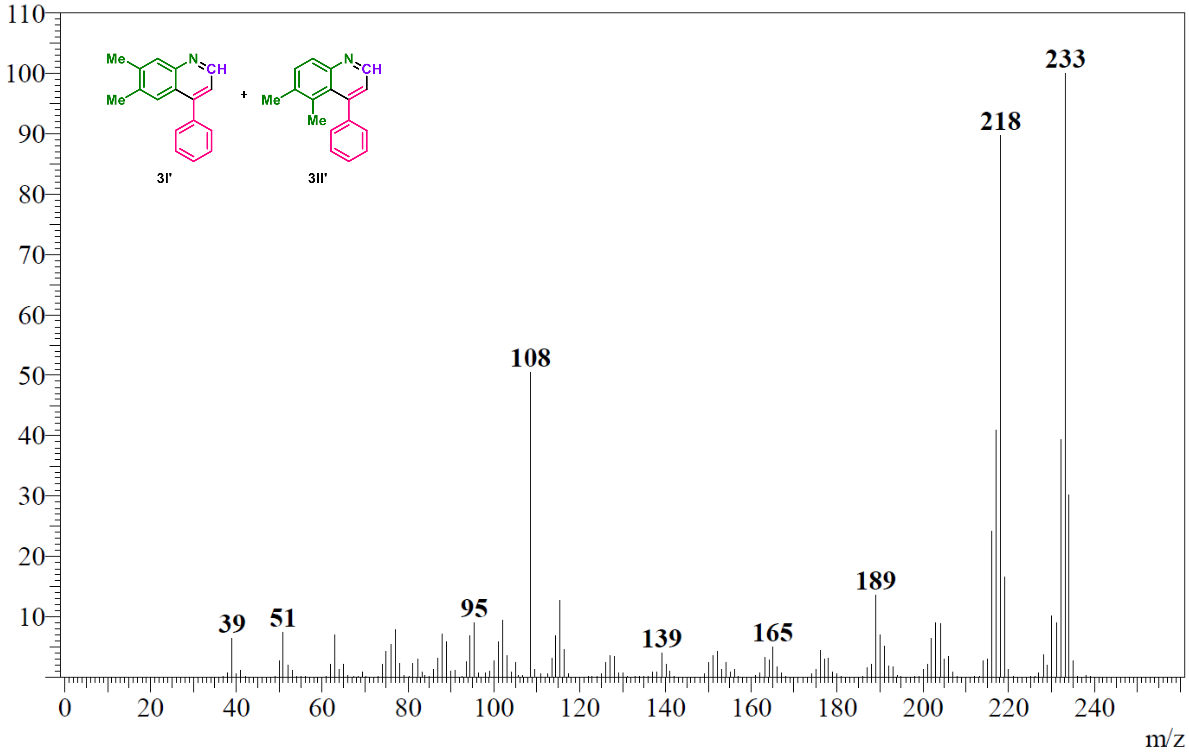

GC–MS spectrum of 4-(4-fluorophenyl)-6-methylquinoline (3q')

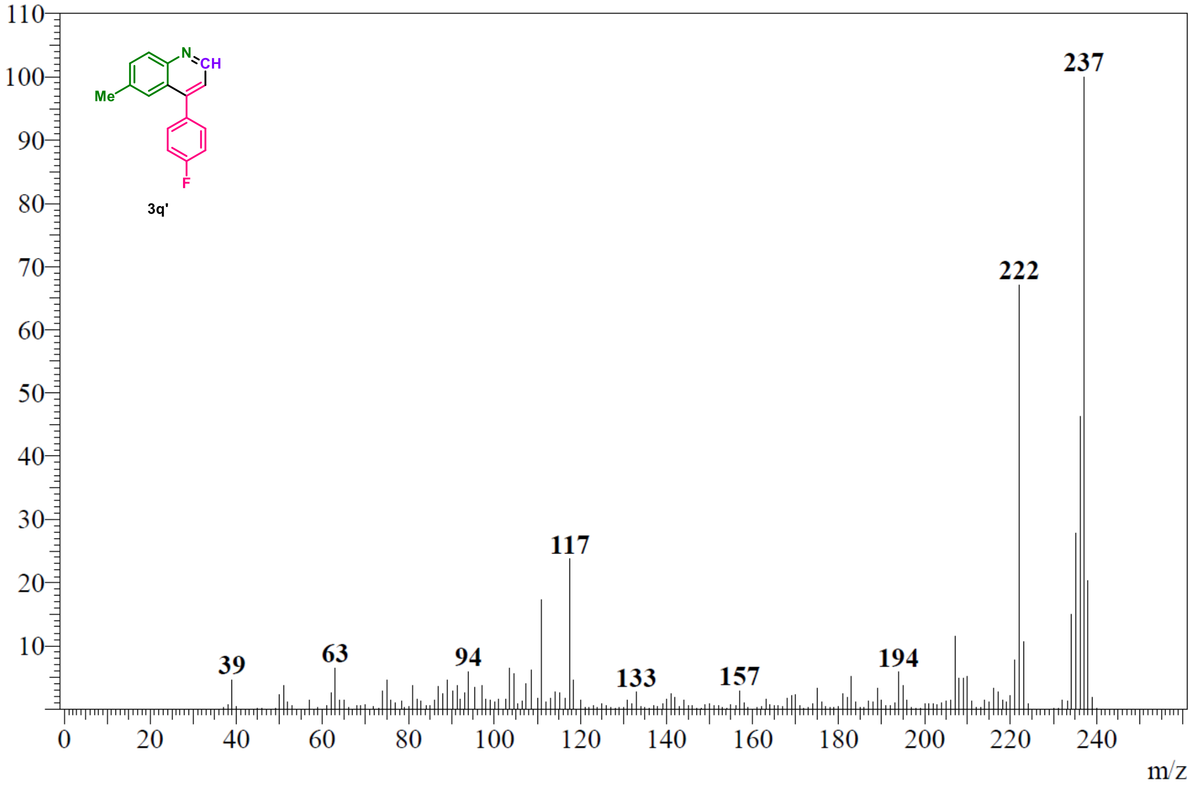

References:

(1) Lee, S. B.; Chun, S.; Choi, S. H.; Hong, J.; Oh, D.-C.; Hong, S. *J. Org. Chem.* **2023**, *88*, 8099–8113. doi:10.1021/acs.joc.3c00095

(2) Chandra, D.; Dhiman, A. K.; Kumar, R.; Sharma, U. *European J. Org. Chem.* **2019**, *2019*, 2753–2758. doi:10.1002/ejoc.201900325

(3) Phanindrudu, M.; Wakade, S. B.; Tiwari, D. K.; Likhar, P. R.; Tiwari, D. K. *J. Org. Chem.* **2018**, *83*, 9137–9143. doi:10.1021/acs.joc.8b01204

(4) Sathyendran, S.; Muthu, K.; Govindan, K.; Chen, N.-Q.; Lin, W.-Y.; Senadi, G. C. *Org. Lett.* **2023**, *25*, 4086–4091. doi:10.1021/acs.orglett.3c01306

(5) Liu, J.-Y.; Wang, Z.-Y.; Li, Q.; Li, D.; Dou, J. *New J. Chem.* **2023**, *47*, 5155–5159. doi:10.1039/D2NJ04119G

(6) Purkait, A.; Saha, S.; Ghosh, S.; Jana, C. K. *Chem. Commun.* **2020**, *56*, 15032–15035. doi:10.1039/D0CC02650F

(7) Jiang, T.-S.; Wang, X.; Zhang, X. *Tetrahedron Lett.* **2018**, *59*, 2979–2982. doi:10.1016/j.tetlet.2018.06.054

(8) Jagtap, P. A.; Sawant, V. R.; Bhanage, B. M. *ChemCatChem* **2024**, *16*. doi:10.1002/cctc.202400979

(9) Faraz, S.; Khan, A. T. *Org. Biomol. Chem.* **2023**, *21*, 7553–7560. doi:10.1039/D3OB00993A

(10) Cheng, D.; Yan, X.; Shen, J.; Pu, Y.; Xu, X.; Yan, J. *Synthesis (Stuttg).* **2020**, *52*, 1833–1840. doi:10.1055/s-0039-1691740
